# Ultrasonic investigation of strongly weathered mudstone subjected to coupled drying-wetting cycles and vibration loading

**DOI:** 10.1371/journal.pone.0361133

**Published:** 2026-10-29

**Authors:** Liqun Zhang, Hang Bai, Honghuan Cui, Song Zhang, Yuan Li, Chunguo Liu, Pengshuai Xu

**Affiliations:** Hebei University of Architecture, Zhangjiakou, Heibei, China; Southwest Jiaotong University, CHINA

## Abstract

In cut slopes composed of strongly weathered mudstone, coupled wetting-drying cycles and traffic-induced vibration readily trigger progressive structural damage and slumping. Conventional single tests—such as isolated dynamic triaxial tests or independent wetting-drying simulations—cannot accurately capture the underlying microstructural evolution. As demonstrated by recent research, such as the experimental investigation of acidic dry-wet cycles on coal-rock combinations, relying on uncoupled testing fails to reproduce synergistic damage mechanisms, highlighting a critical need for multi-field experimental frameworks. Therefore, here we report a new experimental framework in which ultrasonic responses measured during wetting-drying cycling alone and during coupled wetting-drying-vibration loading were compared, including wave velocity, first-arrival amplitude, time-domain features, frequency-domain features and spectral centroid, to identify the deterioration state of remoulded strongly weathered mudstone. Uniaxial compressive strength and elastic modulus were evaluated concurrently to clarify the evolution of hydraulic damage caused by repeated swelling and shrinkage of hydrophilic clay minerals and the associated transition of macroscopic mechanical behaviour towards plastic deformation. The arrival time of the received wave lagged markedly, and under coupled loading the waveform changed from a steep, prominent peak to a right-shifted attenuated response. During wetting-drying cycling alone, the decrease in first-arrival amplitude could be divided into an initial sharp-drop stage and a later slow-accumulation stage. When high-frequency vibration was superimposed, microcracks coalesced more rapidly, and both wave velocity and strength decayed faster. The migration of the frequency spectrum and its centroid towards lower frequencies can serve as a rapid and convenient indicator of different damage states in strongly weathered mudstone. Accelerated microcrack coalescence driven by high-frequency resonance and frequent displacement cycling is the main reason why acoustic attenuation under superimposed vibration is stronger than that under wetting-drying cycling alone, with frequency exerting a greater influence than amplitude. Using wave velocity, first-arrival amplitude and spectral centroid as ultrasonic parameters, we further constructed a dimensionless vibration-disturbance coefficient (I_fA_) based on the entropy-weight method to quantify vibration disturbance under different working conditions, and we suggest that it can be incorporated into a three-dimensional strength-surface model. By bridging non-destructive acoustic monitoring and macroscopic mechanical degradation under coupled environmental-dynamic loading, this research establishes a rapid, non-destructive, and cost-effective quantitative tool for real-time tracking of internal micro-fatigue damage and estimating residual peak strength in weathered rock masses. Ultimately, these findings provide essential theoretical insight and data-driven decision support for long-term stability assessment, risk mitigation, and early warning of transportation corridor slopes subjected to seasonal weathering and vehicle traffic.

## Introduction

### Background

Strongly weathered mudstone is widely distributed across northwestern Hebei and the northern Hebei mountainous region. Due to its pronounced moisture-induced swelling and shrinkage, slopes formed in this material are inherently unstable and highly susceptible to progressive failures, posing severe threats to infrastructure safety and regional environments. Taking Zhangjiakou as a representative case (Figs 1–3), the regional climate is characterized by highly concentrated seasonal rainfall, intense evaporation, and strong wind action. These harsh wetting–drying cycles repeatedly induce swelling and shrinkage of internal hydrophilic minerals, triggering surface cracking, spalling, and progressive degradation of the rock mass. Concurrently, dynamic disturbances from heavy traffic along the Jingxin Expressway (G7) logistics corridor have escalated rapidly in recent years. Specifically, the annual average daily traffic (AADT) grew from approximately 23,500 vehicles/day in 2021 to over 34,500 vehicles/day in 2025, with total annual traffic volume surging from roughly 8.6 million to nearly 12.6 million vehicles (Fig 4). This relentless heavy-vehicle loading exerts a persistent, cumulative dynamic vibration effect on the adjacent slopes.

**Fig 1:**
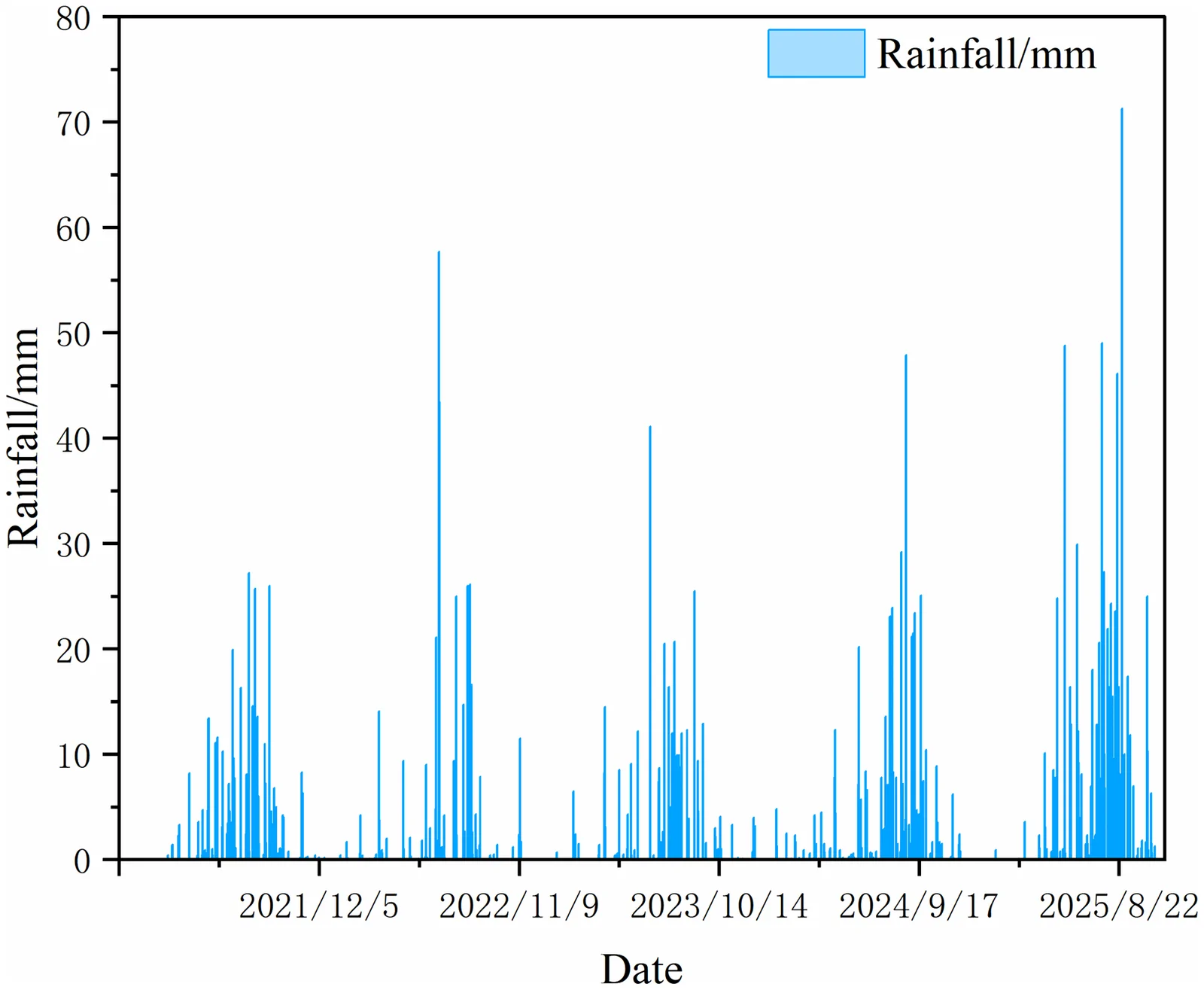
Rainfall.

**Fig 2:**
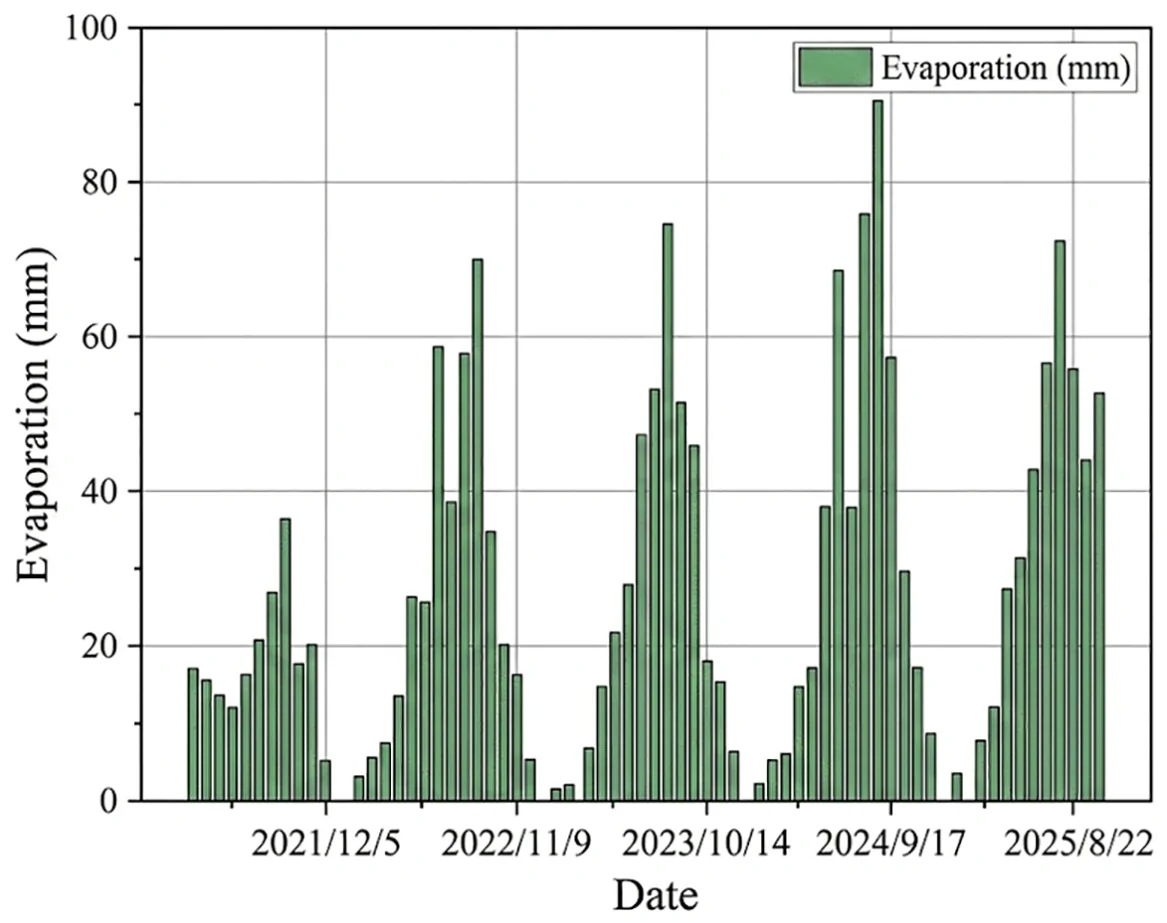
Evaporation.

**Fig 3:**
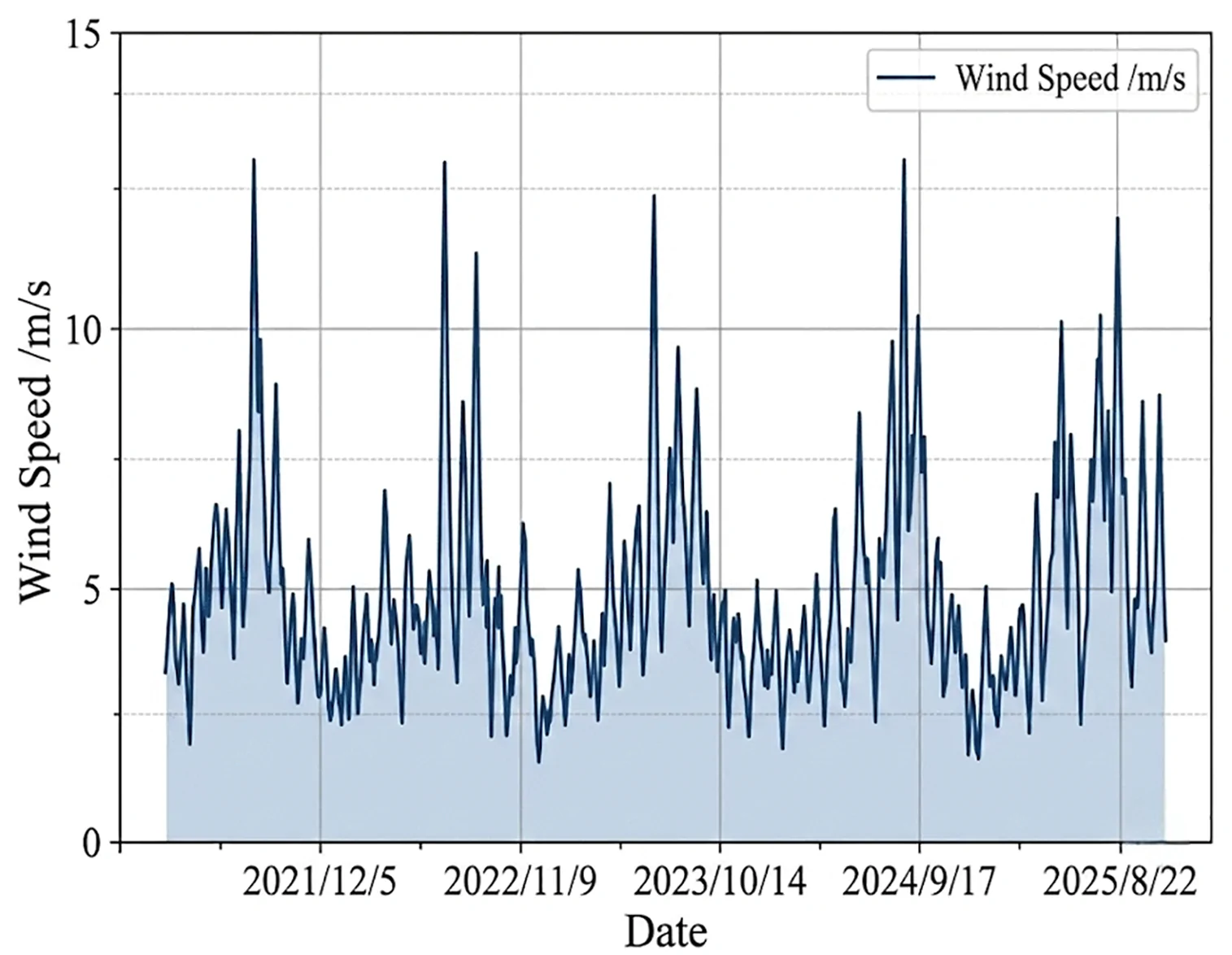
Wind speed.

**Fig 4:**
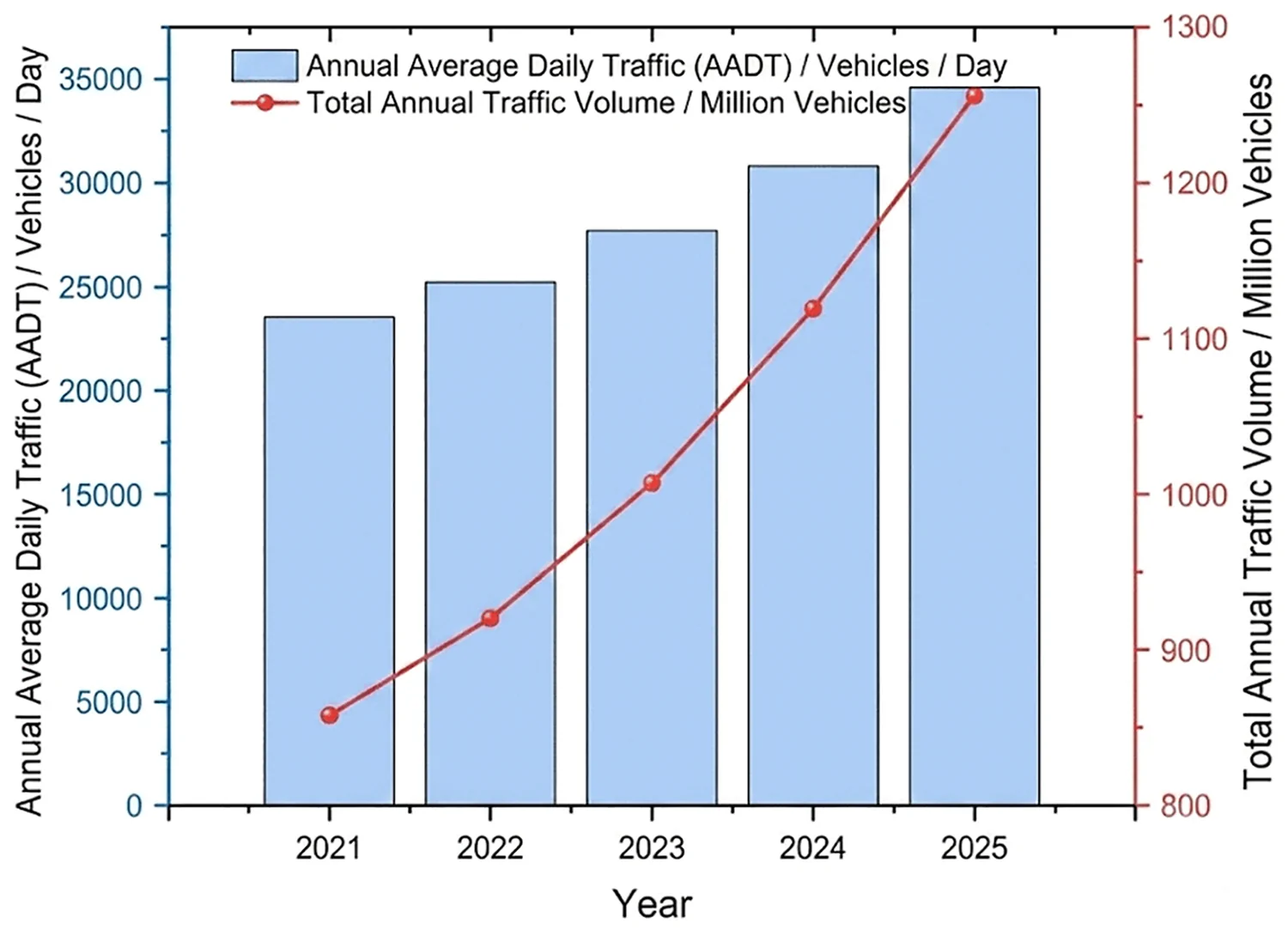
Traffic volume.

As illustrated in Fig 5, atmospheric weathering (driven by evaporation and wind speed) acts synchronously with traffic vibration, creating a complex coupled environmental–dynamic loading environment. Slope instability in strongly weathered mudstone cut slopes is fundamentally governed by the expansion and coalescence of internal microcracks, transitioning from quantitative damage accumulation to qualitative macroscopic failure under this dual disturbance. Crucially, the structural loosening induced by wetting–drying cycles creates a highly vulnerable matrix that amplifies dynamic disturbance, while ongoing traffic vibration further accelerates microcrack propagation and interconnectivity. By clarifying this synergistic deterioration mechanism, the present study not only realistically simulates the damage evolution of mudstone under real-field conditions, but also provides essential data support and a rigorous theoretical foundation for stability assessment and hazard early warning of highway rock slopes in northwestern Hebei.

**Fig 5:**
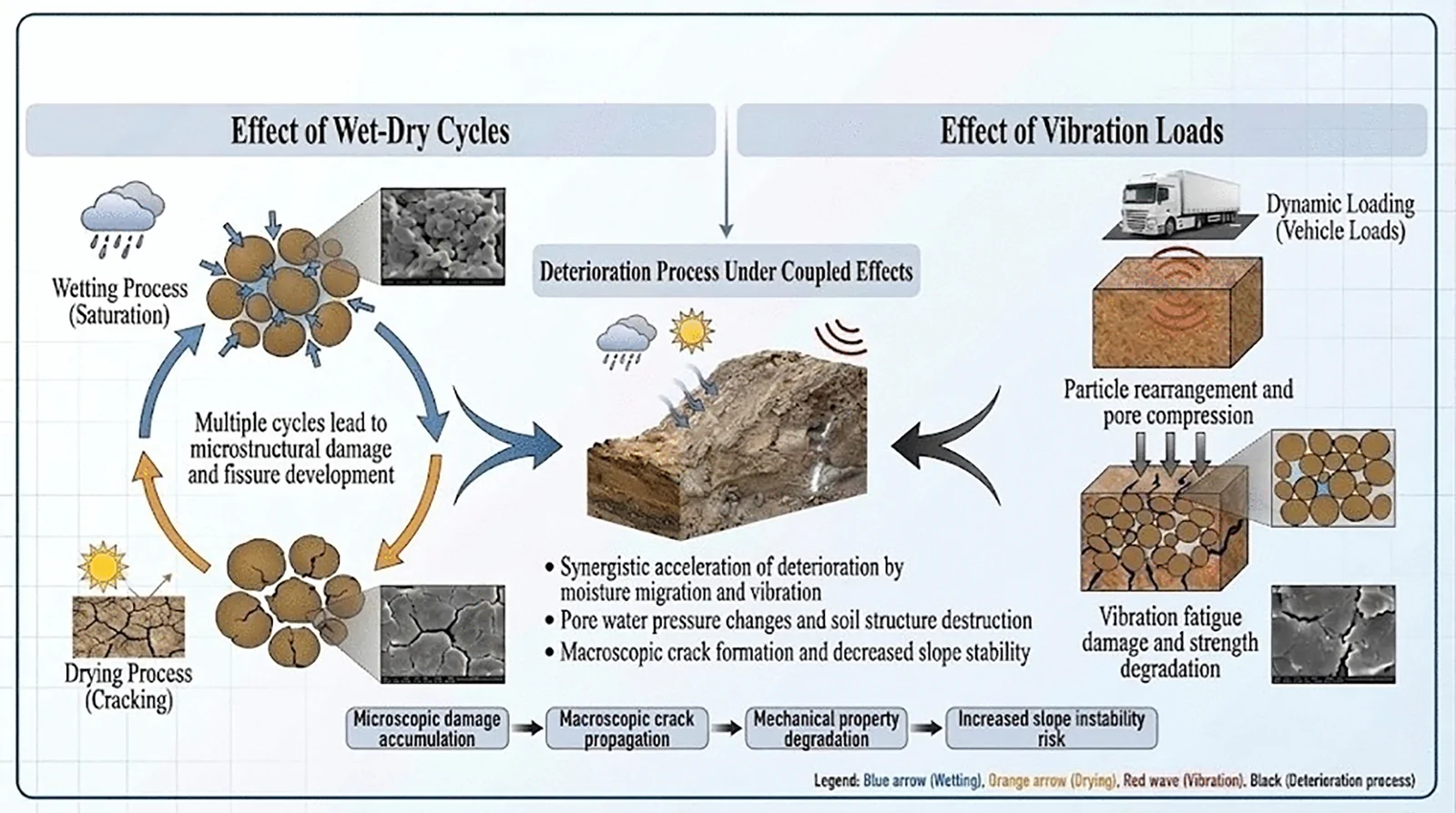
Simulated scenario of coupled wetting-drying cycles and traffic-vibration loading.

### State of the art

The long-term stability issue of highly weathered mudstone cut slopes in the northwestern Hebei region has become increasingly prominent. In the actual service environment, the highly weathered mudstone of the slope is subjected to the long-term coupled effects of seasonal rainfall (wet-dry cycles) and traffic vibration (vibration disturbance), which easily induces progressive structural damage and shallow landslide disasters. In recent years, relevant research by scholars at home and abroad has broken through the limitation of single factors and mainly focused on the following three aspects:

(1) Regarding the impact of wet-dry cycles on the macroscopic mechanical properties of mudstone and rock-soil masses, the frequent migration of moisture leads to repeated volume changes of hydrophilic minerals inside the mass. Studies by Aldaood et al. [1], Wassermann et al. [2], Basma et al. [3], and Doostmohammadi et al. [4] have all confirmed the irreversible weakening effect of wet-dry cycles on its swelling pressure and mechanical strength. Zhou and Liu [5] focused on the evolution law of the swelling pressure of weathered mudstone under cyclic wetting and drying. Erguler and Shakoor [6] quantitatively evaluated the weakening effect of weathering on the mechanical properties of clay-bearing rocks. Cantón et al. [7] and Khan et al. [8] detailed the physical and strength degradation characteristics caused by wet-dry alternation in semi-arid and sedimentary rock environments. Furthermore, Xu et al. [9] and Wang et al. [10] investigated the swelling pressure attenuation and particle breakage laws of mudstone under complex alternating environmental effects. Zeng et al. [11] evaluated the engineering behavior of highly weathered swelling mudstone under seasonal wet-dry alternations, and Zhao et al. [12] conducted a laboratory simulation of the environmental degradation of weak rocks along transport corridors. In China, Zhang Zhizhen [13] systematically explored the mechanical property degradation and energy evolution laws of rocks under wet-dry cycles. Liu Quansheng [14] and Ma Mengtian [15] respectively revealed the meso-mechanisms of hydration swelling, microcrack initiation, and structural loosening caused by wet-dry cycles through laboratory tests. Deng Huafeng et al. [16], Lai Zengwei et al. [17], and Li Shengnan et al. [18] provided a basis for the environmental weathering evaluation of highly weathered mudstone by constructing damage constitutive models or conducting laboratory degradation simulations. Meanwhile, Liu et al. [19] analyzed the degradation characteristics of mudstone strength under the combined action of wet-dry cycles and low-frequency vibration. In recent years, extensive investigations have been conducted on the physical-mechanical property evolution, damage models, and non-destructive evaluation of rock-like and cementitious materials subjected to severe environmental exposures and thermal/mechanical stresses [20–23]. Furthermore, the confinement stress evolution, residual axial performance, and dynamic stress transfer of structural media have been systematically explored to better understand damage accumulation mechanisms under complex loading conditions [24,25]. However, despite these valuable insights into static environmental weathering and low-frequency effects, existing comparative evaluations remain predominantly focused on uncoupled wet–dry exposures. A systematic comparative framework contrasting the accelerated strength and elastic modulus deterioration under dynamic traffic vibrations against traditional static wet–dry weathering remains insufficiently established.
(2) To explore the underlying causes of macroscopic degradation, some studies have delved into the evolutionary mechanisms of microscopic damage. Based on SEM (Scanning Electron Microscopy) technology, Ma et al. [26] and Ghafoori et al. [27] intuitively revealed the microstructural loosening and cementation failure mechanisms of various rocks under wet-dry alternations. Li et al. [28] systematically characterized the evolution of the microscopic pore structure of weathered mudstone combining micro-CT and ultrasonic methods. Furthermore, beyond pure physical degradation, the role of fluid-solid chemical interactions cannot be ignored. Recent research investigating the aging deterioration of mechanical properties on coal-rock combinations [29] has highlighted that hydro-chemical corrosion significantly accelerates microcrack propagation by dissolving internal cements. For strongly weathered mudstone, this implies that chemical dissolution during wet-dry cycles acts synergistically with the physical swelling and shrinkage of hydrophilic minerals. This coupled physical and hydro-chemical degradation further loosens the matrix, rendering the highly weathered mudstone even more susceptible to the fatigue effects of subsequent traffic vibration. Zhang et al. [30] and Wu et al. [31] focused on mudstone composite materials, revealing the evolution laws of their damage constitutive relations and internal permeability characteristics under complex environments. In terms of multi-field coupling and dynamic response, cyclic dynamic loading has a significant catalytic effect on the expansion of microcracks. Zhao et al. [32] explored the initiation of micromechanical damage and the evolution of fatigue recovery of rocks under cyclic loading. Li et al. [33] and Momeni et al. [34] quantified the significant weakening of rock fatigue damage and mechanical strength caused by alternating stress and cyclic loading. Li Ping et al. [35] investigated the dynamic characteristics of solidified sludge under multi-field coupling such as wet-dry cycles, pointing out that dynamic stress accelerates the coalescence of microscopic cracks. Regarding the dynamic disturbance caused by traffic loads, numerous scholars have pointed out that its catalytic effect on the expansion of internal microcracks in slopes is highly frequency-dependent. Yang et al. [36] and Gratchev et al. [37] elucidated the instability mechanisms of highly weathered mudstone landslides and slope degradation triggered by cumulative vibrations from the reciprocal driving of heavy vehicles. Cerfontaine et al. [38] and Heap et al. [39] further quantified the fatigue accumulation and acoustic emission response laws during cyclic degradation. Hashim et al. [40] and Song et al. [41] systematically explored the control mechanisms of frequency and amplitude elements of dynamic disturbances on the physical fatigue loss within weak rocks, pointing out that high-frequency cycling is the core driving force for the fatigue degradation of microscopic contact surfaces.
(3) In terms of non-destructive testing and microscopic damage evaluation, ultrasonic testing technology can indirectly reflect the physical and mechanical parameters and internal evolution characteristics of media by measuring the acoustic signal changes after ultrasonic transmission through rocks and soils. It is widely used due to its high sensitivity, non-destructiveness, and rapidness. While passive acoustic emission (AE) techniques are widely applied to dynamically monitor real-time microcrack propagation and energy release during rock fracturing [42], active ultrasonic testing provides a non-destructive evaluation of full-domain elastic wave attenuation, velocity variation, and microstructural deterioration induced by environmental and dynamic loading.To achieve dynamic tracking of damage, ZHANG et al. [43] and MENG et al. [44] deeply combined destructive compression with ultrasonic testing, verifying the close correlation between acoustic parameters and internal initial damage in rocks. GU et al. [45] and Zhao Juan et al. [46] quantitatively revealed the acoustic transit time lag and wave velocity degradation mechanisms caused by pore development under wet-dry cycles. Sun et al. [47], Martínez-Martínez et al. [48], and Gomez et al. [49] effectively evaluated the physical and mechanical damage of rocks caused by cyclic loading and natural weathering using ultrasonic testing methods. On this basis, extracting and analyzing the time-frequency characteristics of ultrasonic waves can more deeply reveal the micro-evolution mechanisms of geological materials under multi-field coupled disturbances. For example, Zhang et al. successfully evaluated the physical states and internal interface evolution of fine sand, sandstone, and loess during freezing processes using ultrasonic wave velocity, amplitude, and time-frequency characteristics [50–52]. Wang Kun et al. explored the mechanical properties and pore degradation laws of frozen rock masses under confining pressure [53]. Wang Peng et al. deeply analyzed the ultrasonic time-frequency characteristics of heat-damaged sandstone based on wavelet transform, quantitatively characterizing the internal damage degree through spectral characteristic parameters [54]. Wang et al. [55] established a time-frequency feature analysis method for ultrasonic signals in damaged sedimentary rocks under environment-load coupling. Zhang et al. [56] proposed using the spectral centroid features of received ultrasonic signals as a convenient and rapid method to identify different damage states of microcrack initiation, propagation, and accelerated coalescence within highly weathered mudstone. However, existing research on ultrasonic time-frequency characteristics and degradation mechanisms mostly focuses on sand, loess, and general rocks. Studies on the ultrasonic response laws and microscopic damage mechanisms of highly weathered mudstone under the severe coupled conditions of wet-dry cycles and traffic vibration remain relatively scarce.

Because the coupled action of wetting-drying cycles and traffic-induced dynamic loading is the central trigger of instability in strongly weathered mudstone slopes, a comprehensive testing strategy is essential. However, conventional single tests—such as isolated dynamic triaxial tests, standalone uniaxial compression tests, or independent wetting-drying simulations under static conditions—cannot accurately capture the underlying microstructural evolution. These uncoupled testing approaches isolate physical or environmental factors and fail to reproduce the synergistic damage mechanisms occurring under real-field coupled disturbances. As highlighted by recent investigations into complex environmental impacts on rock degradation, such as the experimental study of acidic dry–wet cycles on coal-rock combinations [57], coupled testing frameworks are indispensable for revealing the true mechanical performance and microstructural deterioration. To address this clear research gap, we propose a damage-mechanics framework that tightly integrates vibration-table tests, mechanical experiments and ultrasonic non-destructive testing. Remoulded strongly weathered mudstone specimens were prepared, natural wetting-drying cycles were simulated using forced-air drying and vacuum saturation, and traffic disturbance with different frequencies and amplitudes was accurately applied using a four-degree-of-freedom electromagnetic vibration table to reproduce the slope service environment. At different stages of damage evolution under coupled loading, ultrasonic testing was introduced to systematically extract time-domain and frequency-domain features together with wave velocity, first-arrival amplitude and spectral centroid. On this basis, we establish for the first time a quantitative relationship between non-destructive ultrasonic indicators and macroscopic mechanical damage under coupled wetting-drying and traffic-vibration loading, enabling non-destructive, real-time and quantitative assessment of slope damage. This framework is then used to infer the initiation, propagation and coalescence of internal microcracks in mudstone, thereby providing theoretical and technical support for damage evaluation, stability prediction and hazard prevention in slope engineering. Furthermore, by directly benchmarking our experimental findings against classic uncoupled degradation models reported in existing literature, this framework clarifies the distinct synergistic damage mechanism induced by coupled environmental-vibration conditions.

### Research significance and novelty

Executing this study requires key experimental prerequisites: controlled wetting–drying cycling, multi-frequency vibration loading, and active full-waveform ultrasonic acquisition. Compared to traditional static or uncoupled testing, the primary novelties of this work include: (1) capturing the synergistic deterioration mechanism where wetting–drying structural loosening amplifies traffic dynamic disturbances; (2) establishing a highly sensitive, non-destructive bridge between spectral centroid shifts and micro-defect evolution; and (3) integrating an entropy-weight vibration-disturbance coefficient (I_fA_) into a 3D strength-surface model for slope safety early warning.

## Materials and methods

### Specimen material

#### Source and mineral composition of the rock samples.

Most cut slopes in northwestern Hebei are composed of strongly weathered mudstone. This material occupies a transitional rock-soil state: it is not fully weathered, yet it is close to complete weathering. Under dry conditions it remains relatively hard and rock-like, whereas upon contact with water it rapidly disintegrates and softens into soil. Because the mudstone occurs at shallow depth and is disturbed by wetting-drying cycles, traffic loading and overburden loading, it typically exhibits a semi-rock, semi-soil character.

The rock samples used in this study were collected from a cut slope along the Jingxin Expressway, K196 + 324-K196 + 414, in the Nihewan area of Zhangjiakou, Hebei Province, as shown in Fig 6 and Fig 7. The geological setting of this area is complex and weathered mudstone is widely distributed. Fig 8 shows the particle size distribution curve of the mudstone sample. X-ray diffraction patterns (Fig 9) indicate that the specimens contain abundant illite and montmorillonite. These clay minerals are strongly hydrophilic: they swell and soften readily upon wetting and shrink and crack upon drying, which is the principal mineralogical reason for the poor disintegration resistance and strong water sensitivity of this rock type.

**Fig 6:**
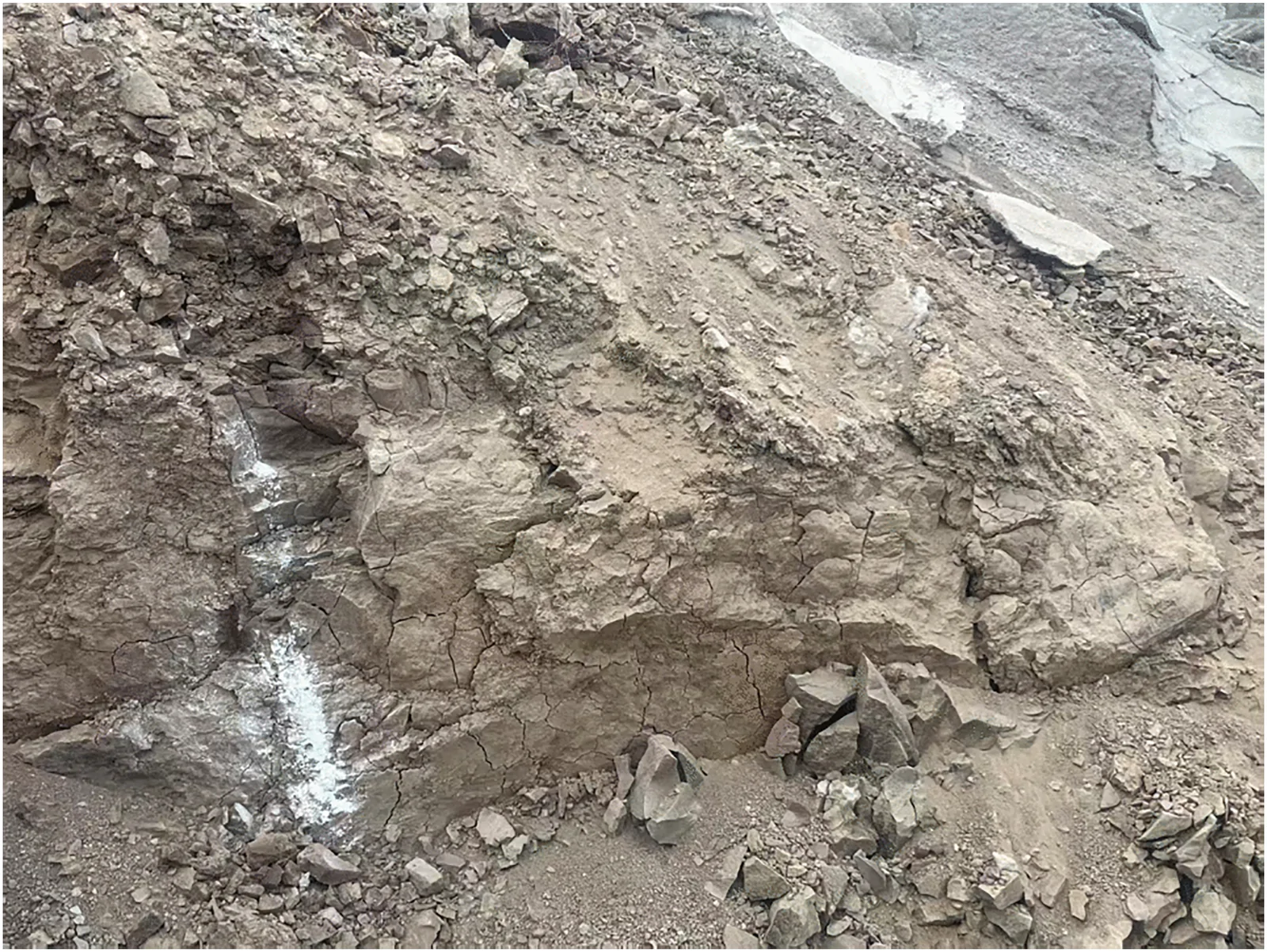
Cut slope.

**Fig 7:**
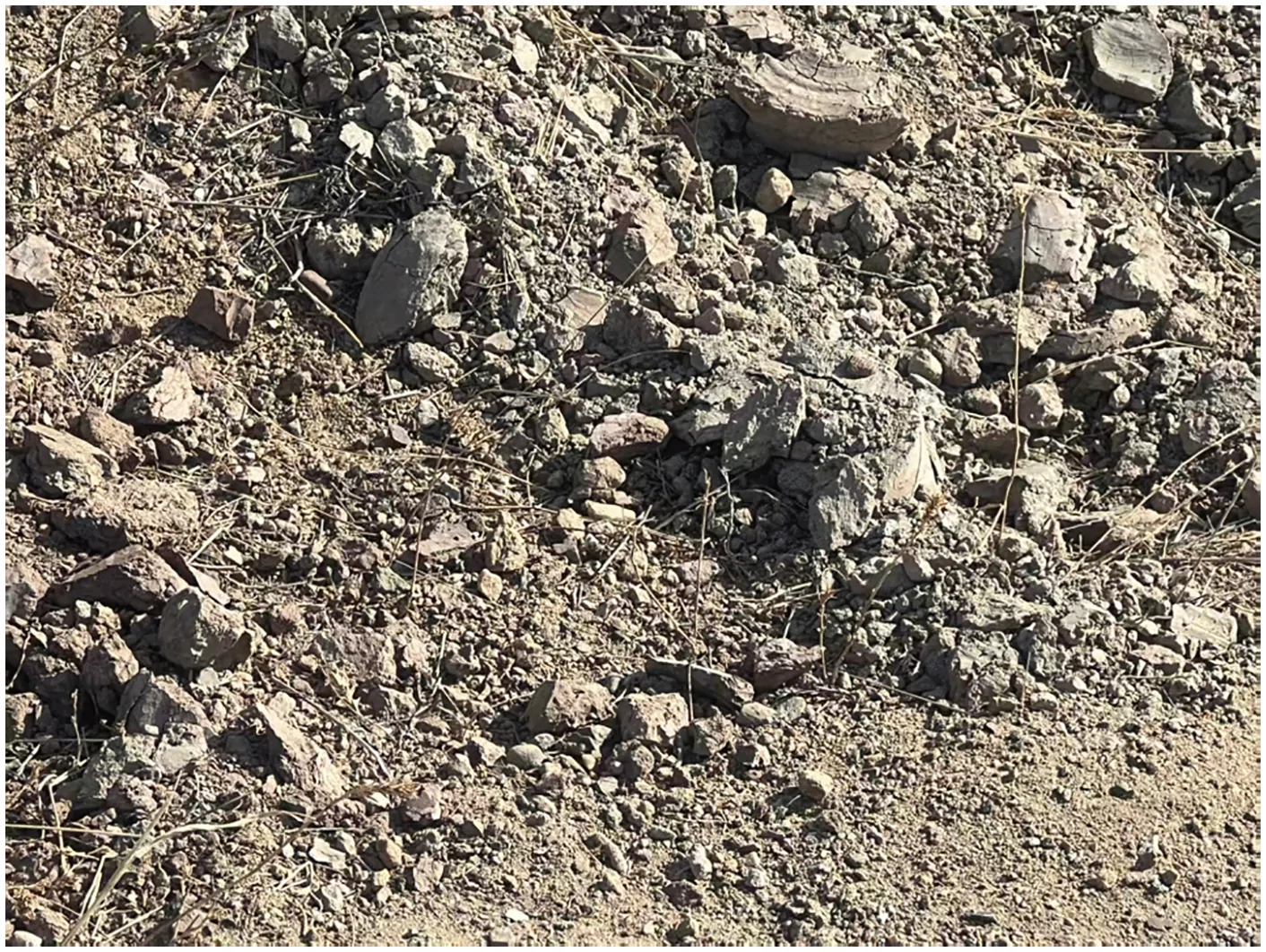
Blocky strongly weathered mudstone.

**Fig 8:**
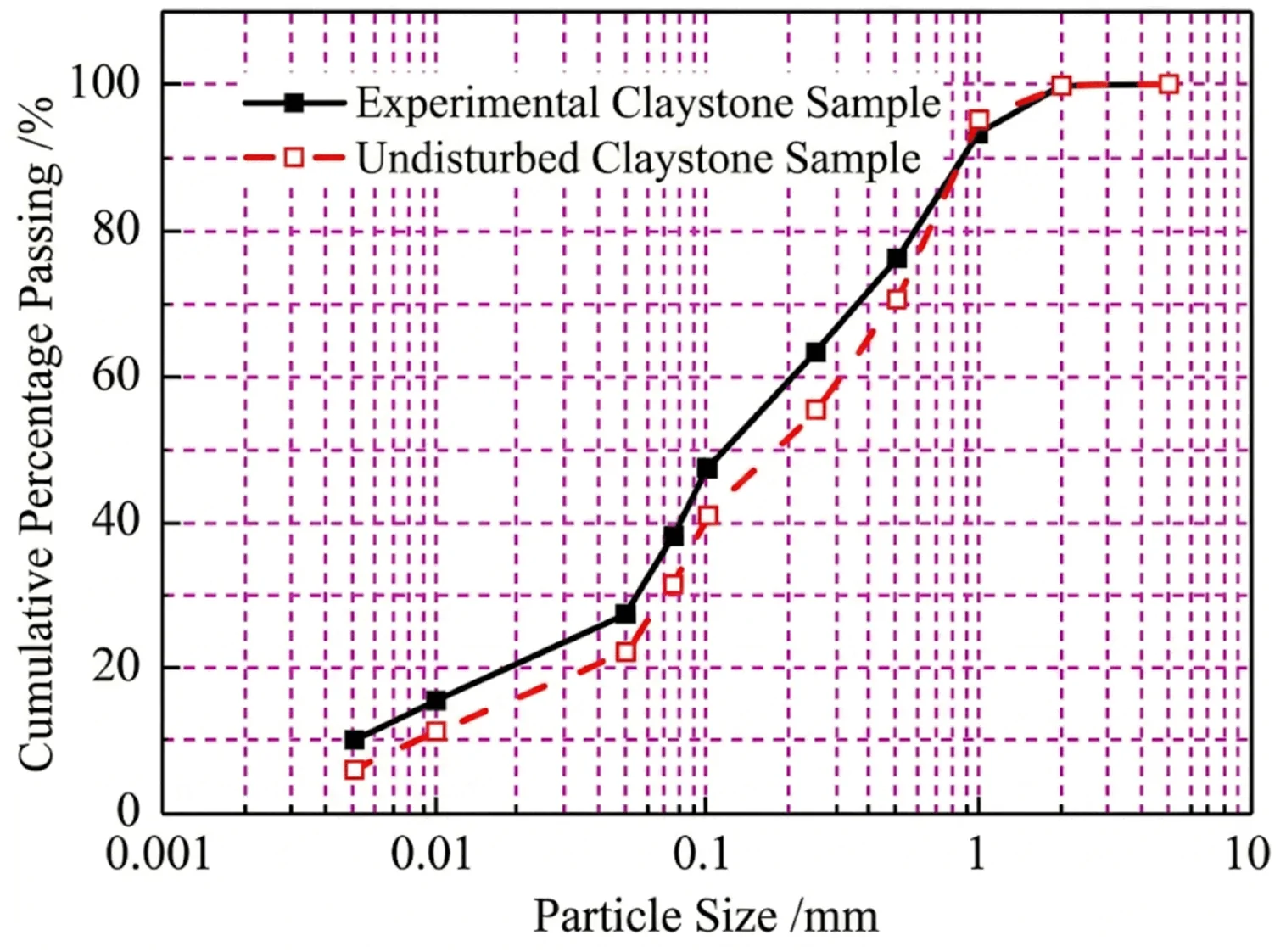
Particle gradation curve.

**Fig 9:**
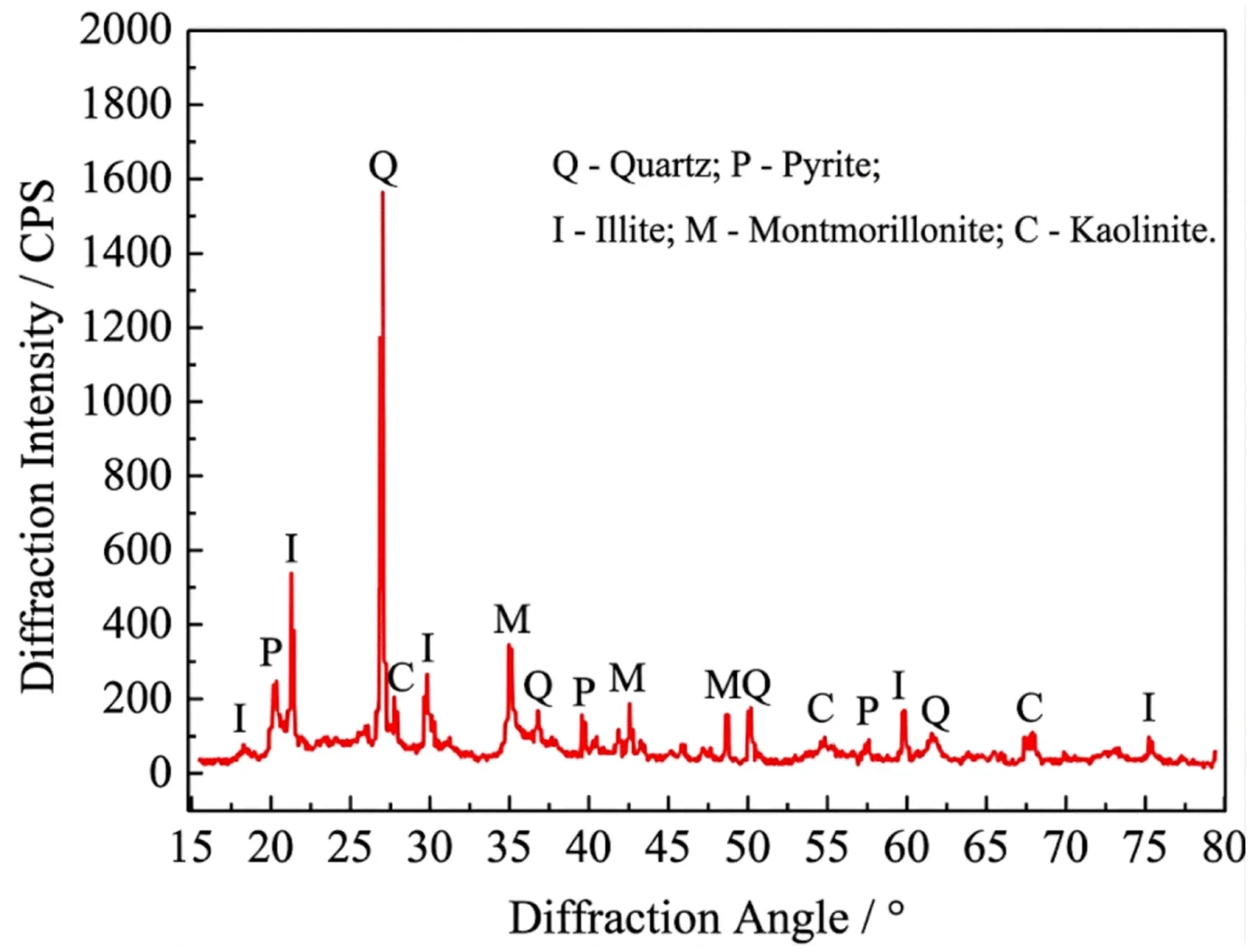
X.ray diffraction pattern.

### Physical properties

The collected blocky strongly weathered mudstone was crushed and passed through a 2 mm sieve, and its basic physical properties were measured, as listed in Table 1. The liquid limit is 38.9%, the plastic limit is 24.7%, the plasticity index is 14.2, and the free swelling ratio reaches 54.5%, indicating a highly expansive soil. The optimum water content is 18.5%, and the maximum dry density is 2.06 g/cm3.

**Table 1:** Basic physical properties of the strongly weathered mudstone specimens.

Liquid limit/%	Plastic limit/%	Relative density of strongly weathered mudstone	Free swelling ratio/%	Natural water content/%	Optimum water content/%	Maximum dry density/g/cm3	Plasticity index
38.9	24.7	2.74	54.5	22.5	18.5	2.06	14.2

### Specimen preparation

Because the random distribution of natural fissures in undisturbed strongly weathered mudstone can produce substantial scatter in experimental results, the rock was crushed and passed through a 2 mm sieve to prepare remoulded specimens with improved homogeneity. The specimens were 50 mm in diameter, 80 mm in height and compacted to 95% of maximum compaction for the mechanistic study. The preparation procedure is shown in Fig 10.

**Fig 10:**
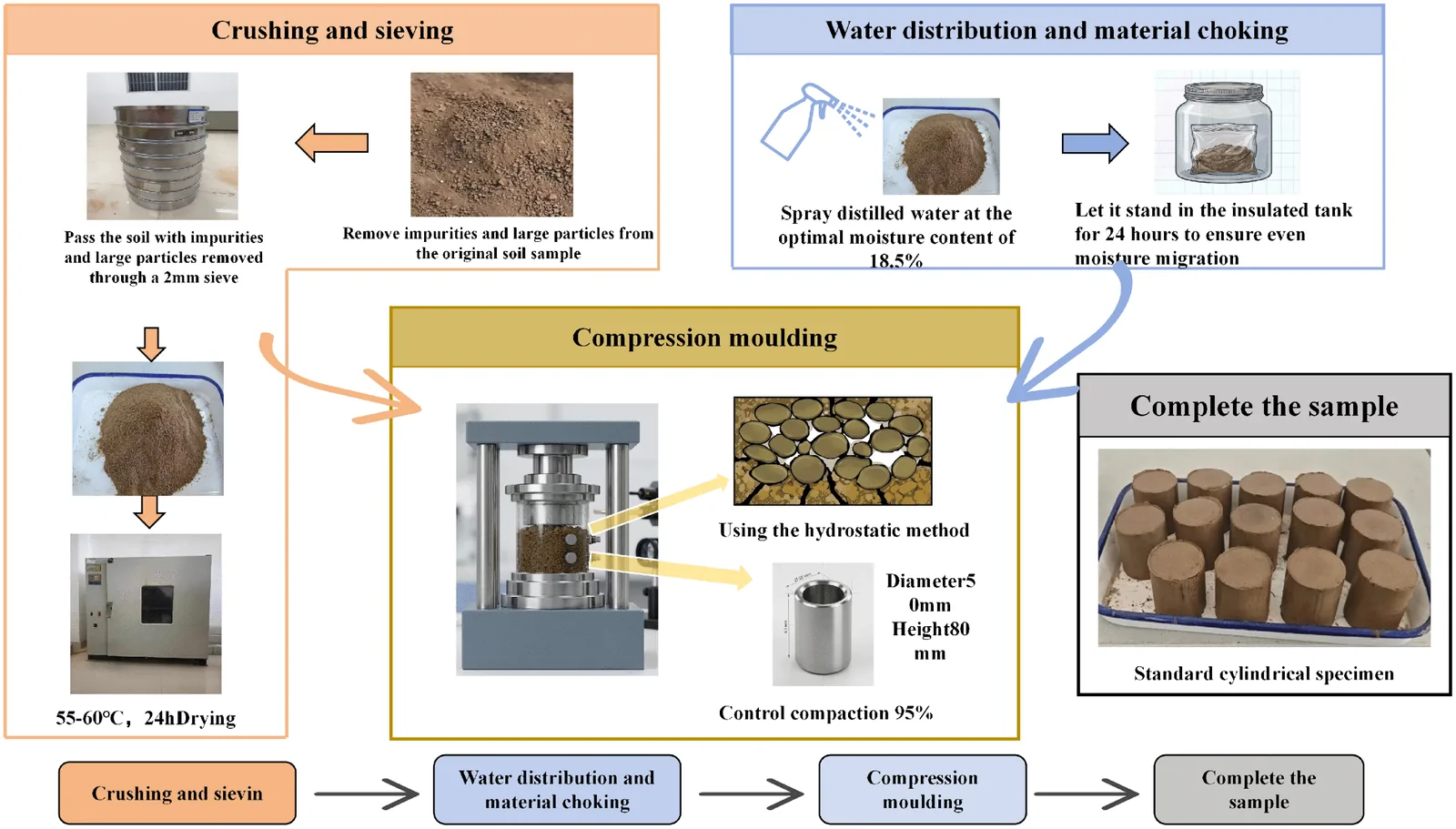
Preparation procedure for strongly weathered mudstone specimens.

### Experimental equipment

(1) Wetting-drying cycle simulation system:

Drying subsystem: a precision constant-temperature forced-air drying oven was used, with the temperature set to 55–60 °C. This specific temperature range was selected to reflect actual field conditions; while ambient summer air temperatures in northwestern Hebei are lower, direct and prolonged solar radiation on exposed cut slopes significantly elevates the surface temperature of the heat-absorbing mudstone to approximately 55–60 ℃. This setting effectively simulates the intense moisture evaporation driven by the local hot and dry environment. Furthermore, establishing precise thermal boundary conditions is essential for accurately capturing the temperature field distribution and its physical effects on geomaterials, a methodological necessity that has been similarly emphasized in recent numerical simulations of temperature fields in loose and soft strata [58]. Therefore, this simulated environment accurately captures the thermal dynamics driving the shrinkage of hydrophilic minerals.

Wetting subsystem: a vacuum saturation device combined with spray humidification was used to ensure uniform water uptake and saturation from the specimen surface to the core, as shown in Fig 11 and Fig 12.

**Fig 11:**
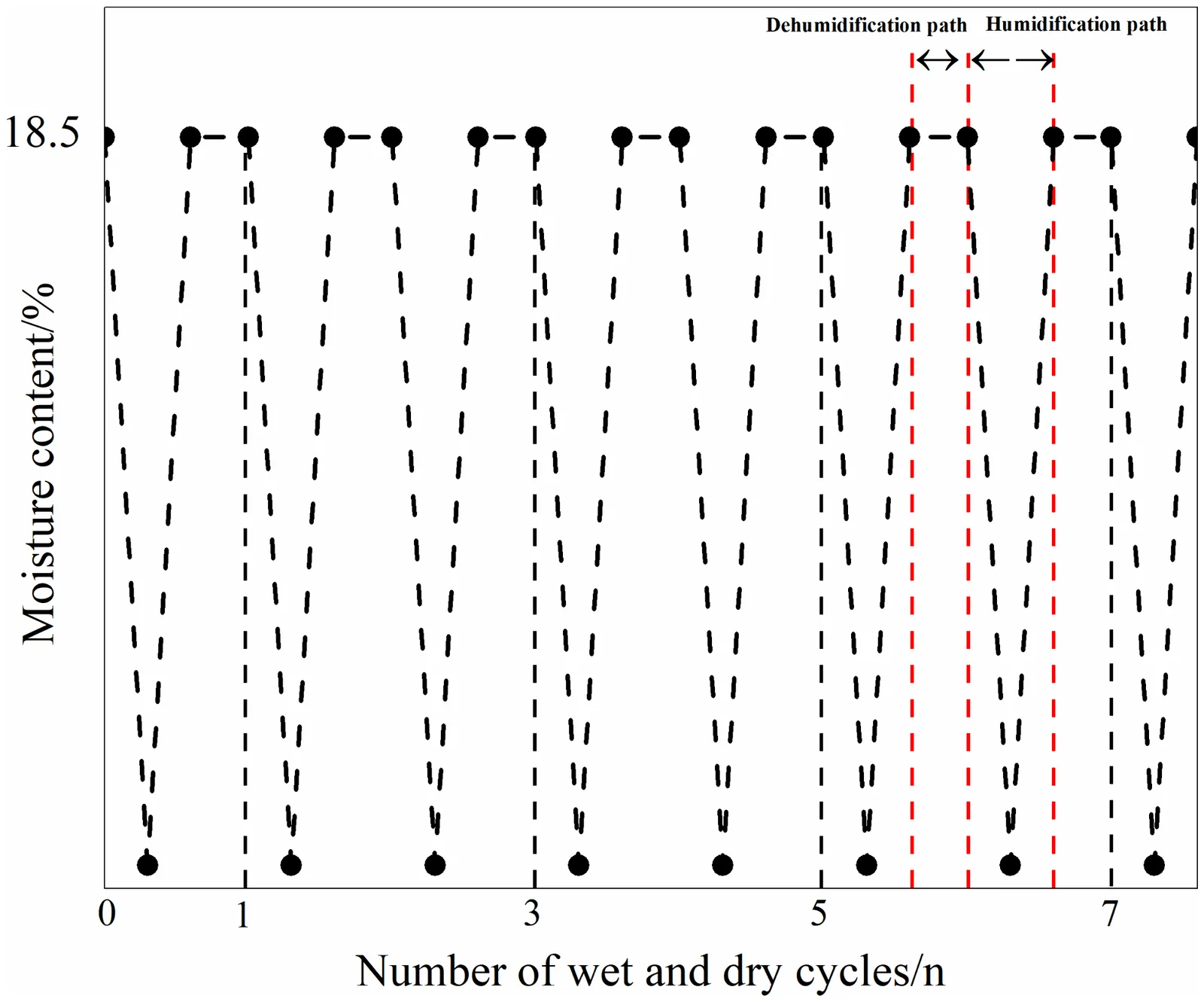
Dry-Wet Cycle Path Flowchart.

**Fig 12:**
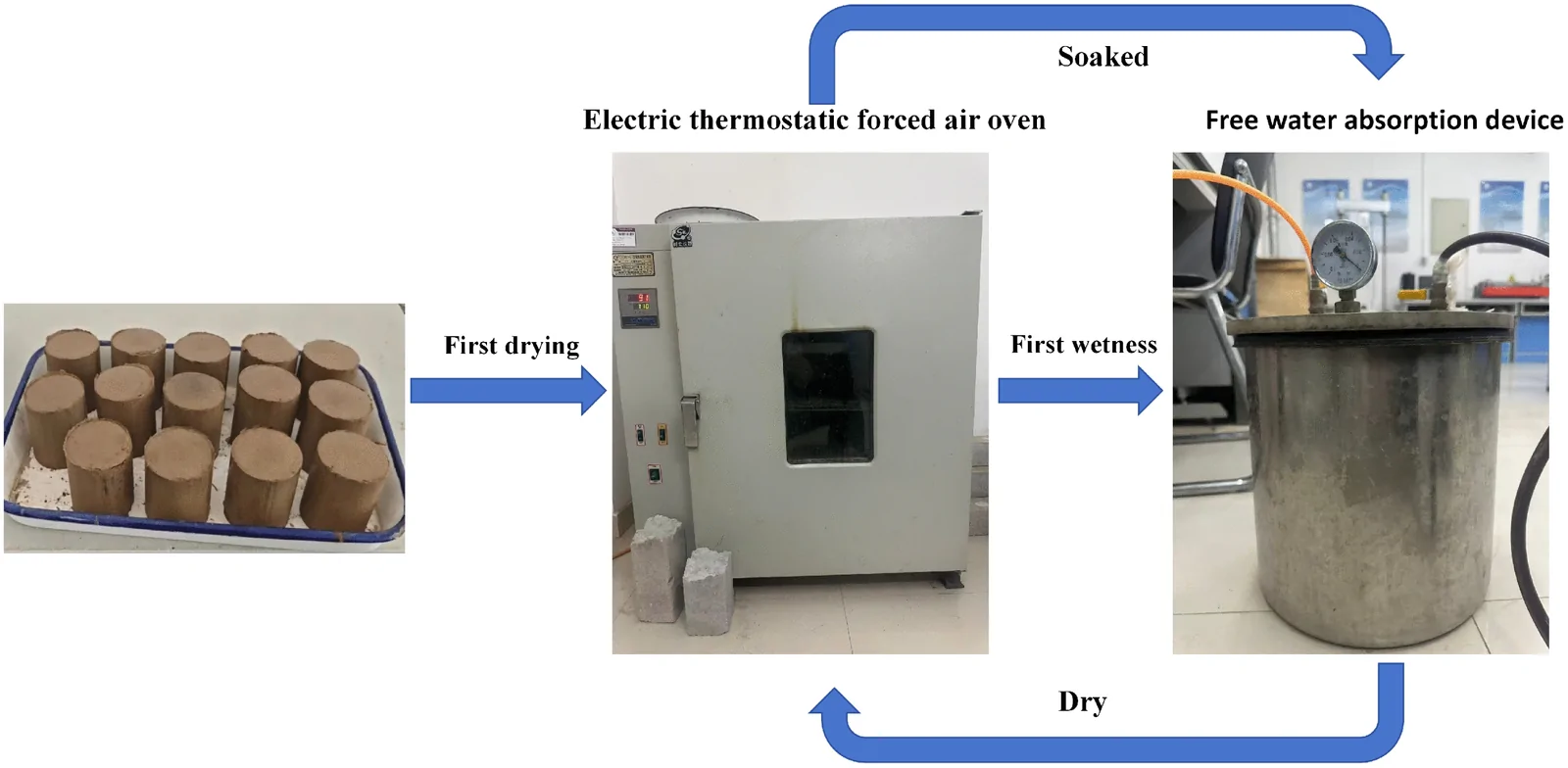
Dry-Wet Cycle Path Entity Diagram.

(2) Vibration loading system: a four-degree-of-freedom electromagnetic vibration table manufactured by Suzhou Shuozhou Technology Co., Ltd. was used to apply vertical vibration loading. The system consists of a vibration controller, vibration table and vibration model box. The model box measures 250 × 145 × 125 mm and was rigidly fixed to the table surface with bolts to ensure loss-free transmission of vibration energy to the specimens.To simulate traffic-induced dynamic stress, the vibration loading system was operated under controlled frequencies and amplitudes, with detailed experimental parameters summarized in Table 2. The test frequencies (f = 9–22 Hz) and dynamic amplitudes (A = 0.5–2 mm) were deliberately selected to represent typical dynamic stress waves induced by subgrade traffic loads. In field conditions, traffic vibrations transmitted to subgrade slopes attenuate rapidly, with dominant frequencies typically falling within the range of 9–22 Hz depending on vehicle speed, axle load, and depth. Therefore, the parameter combination in Table 2 realistically captures the cyclic fatigue conditions experienced by strongly weathered mudstone cut slopes under passing vehicle loads.”
(3) Mechanical testing system: uniaxial compression was carried out using a YAW-100 small pressure-testing machine manufactured by Jilin Guanteng Automation Technology Co, Ltd. The apparatus comprises the loading frame, control system, software, oil pump and computer. The test process was computer controlled, and the uniaxial loading rate was set to 1 mm/min.
(4) Ultrasonic testing was performed using a ZT802 non-metallic ultrasonic detector and analyser in direct transmission mode across both planar end faces of the cylindrical specimens (L = 80 mm). This intelligent instrument applies ultrasonic pulse testing to non-metallic materials and components such as concrete, rock, ceramics, graphite and plastics. It integrates ultrasonic emission, dual-channel synchronous reception, high-speed digital signal acquisition, automatic acoustic-parameter detection, data processing, real-time result display, data storage and output, and was used for subsequent spectral analyses. The instrument was configured with a high-voltage impulse excitation of 1000 V, a sampling interval of 0.8μs (sampling frequency of 1.25 MHz), and a record length of 512 points (4096μs). The system zero-time delay (T_0_ = 1.61μs) was calibrated using a standard steel block.Operationally, the time-series ultrasonic signals were continuously monitored and recorded by the device during testing. The recorded waveform data were exported to a PC via a USB storage drive and subsequently processed using specialized waveform analysis software to extract t0 and the corresponding acoustic parameters. Specifically, the first-arrival amplitude (A_1_) is defined as the maximum peak-to-peak amplitude captured within the first full (or half) cycle time window, starting immediately from the initial arrival time (t_0_) of the waveform.

**Table 2:** Experimental programme.

Test group	Wetting-drying cycles	Vibration frequency/Hz	Vibration amplitude/mm	Vibration duration/s
Wetting-drying-only group	0.1.3.5.7	0	0	0
Variable-frequency vibration-coupled group	0.1.3.5.7	9.12.18.22	1.0 (fixed)	500
Variable-amplitude vibration-coupled group	0.1.3.5.7	18 (fixed)	0.5.1.0.1.5.2.0	500

### Experimental programme and procedure

According ton the Technical Standard of Highway Engineering (JTG B01), local climatic conditions of northwestern Hebei, and actual slope-soil conditions, the experimental program was divided into three test groups to evaluate deterioration induced by standalone wetting–drying cycling versus coupled vibration factors (frequency and amplitude), as summarized in Table 2. The complete dynamic testing workflow—spanning environmental preconditioning, active acoustic monitoring, dynamic vibration loading, and final strength evaluation—is schematically shown in Fig 13.

**Fig 13:**
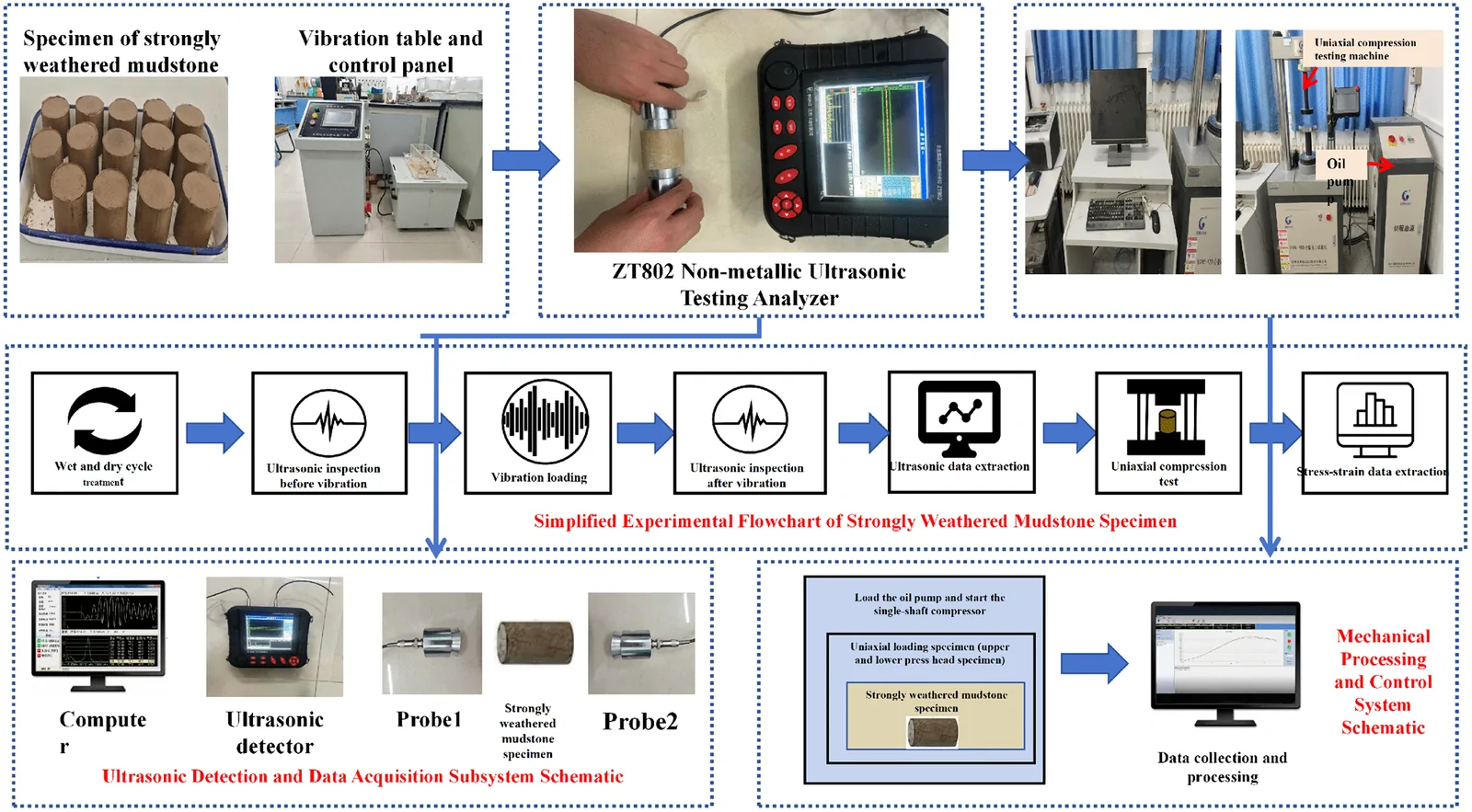
Experimental flow chart.

Specifically, the wetting-drying-only group was designed to clarify the damage effect of wetting-drying cycles alone on strongly weathered mudstone; the variable-frequency vibration-coupled group was designed to reveal the coupled deterioration induced by different vibration frequencies on top of wetting-drying cycling; and the variable-amplitude vibration-coupled group was designed to evaluate the coupled deterioration induced by different vibration amplitudes on top of wetting-drying cycling.

### Mechanical behaviour analysis

#### Effect of wetting-drying cycles on the stress-strain curves.

When the specimens were subjected only to wetting-drying cycles, the stress-strain curves shown in Fig 14 indicate a pronounced deterioration in the mechanical behaviour of strongly weathered mudstone. The peak stresses after 0, 1, 3, 5 and 7 cycles were 27.00, 22.49, 18.49, 11.54 and 10.51 MPa, respectively. Relative to the uncycled specimens, the corresponding reductions in peak stress were 16.7%, 31.53%, 57.29% and 61.07%. Without wetting-drying cycling, the specimens exhibited the highest peak stress and strongest compressive resistance, together with a steep stress-strain curve indicative of high stiffness. After one cycle, the peak stress declined to 22.49 MPa and the curve became slightly flatter, suggesting the onset of microstructural damage. After three cycles, the peak stress fell further to about 18.49 MPa and the flatter curve reflected enhanced strain capacity and a transition from brittle to more plastic behaviour. After five cycles, the peak stress dropped sharply to about 11.54 MPa; the curve was the flattest and strain capacity was strongest, indicating clear structural softening and enhanced plastic deformation. After seven cycles, the peak stress decreased further to 10.51 MPa, and the continued flattening of the curve indicates even more pronounced plastic deformation and deeper structural softening.

**Fig 14:**
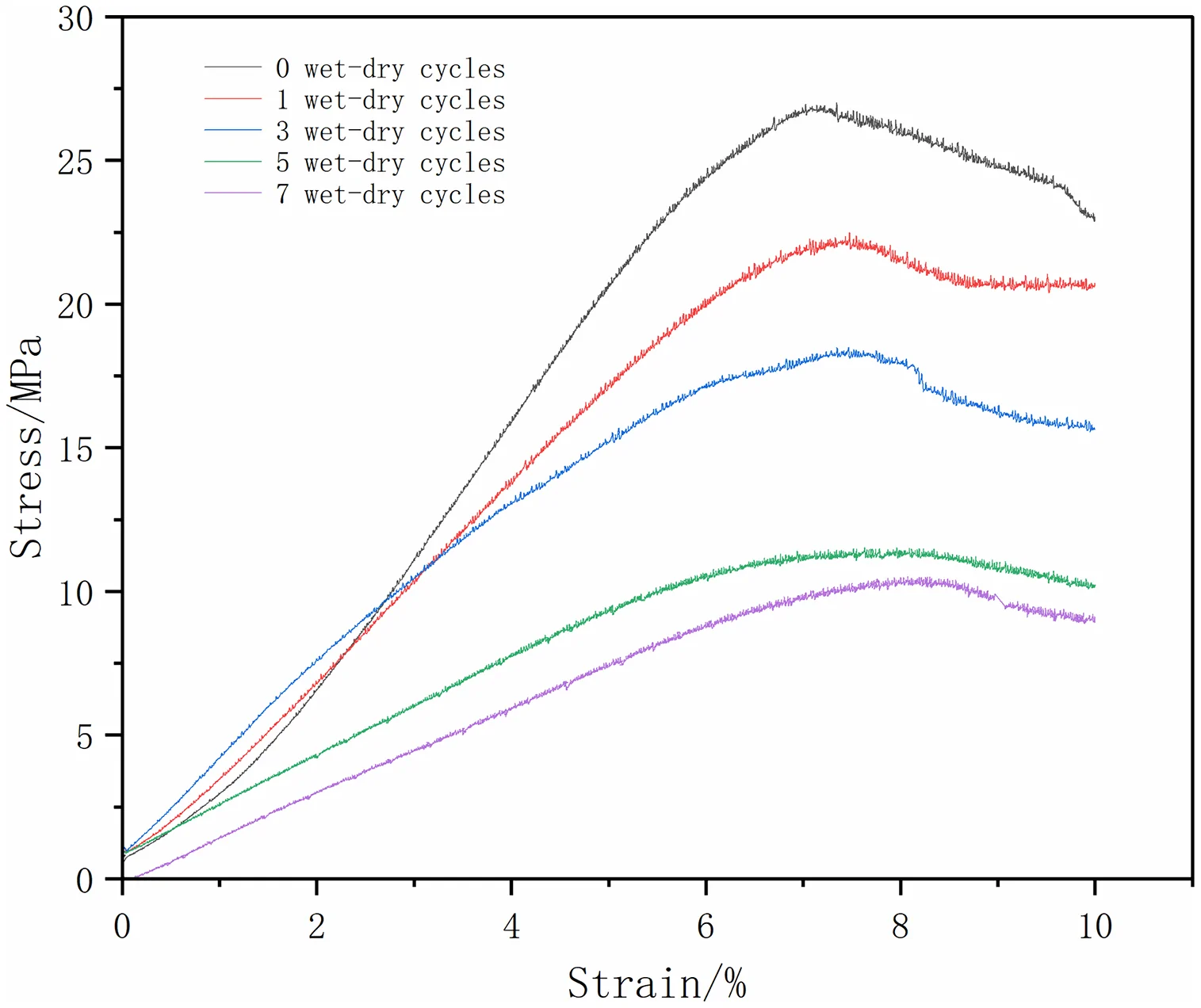
Peak stress under different wetting-drying cycles.

### Stress-strain curves under wetting-drying cycling plus vibration disturbance

Figs 15–19 shows the stress-strain curves under combined wetting-drying cycling and vibration disturbance. In the legend, 9−1 denotes a vibration frequency of 9 Hz and a vibration amplitude of 1 mm; the remaining labels follow the same convention.

**Fig 15:**
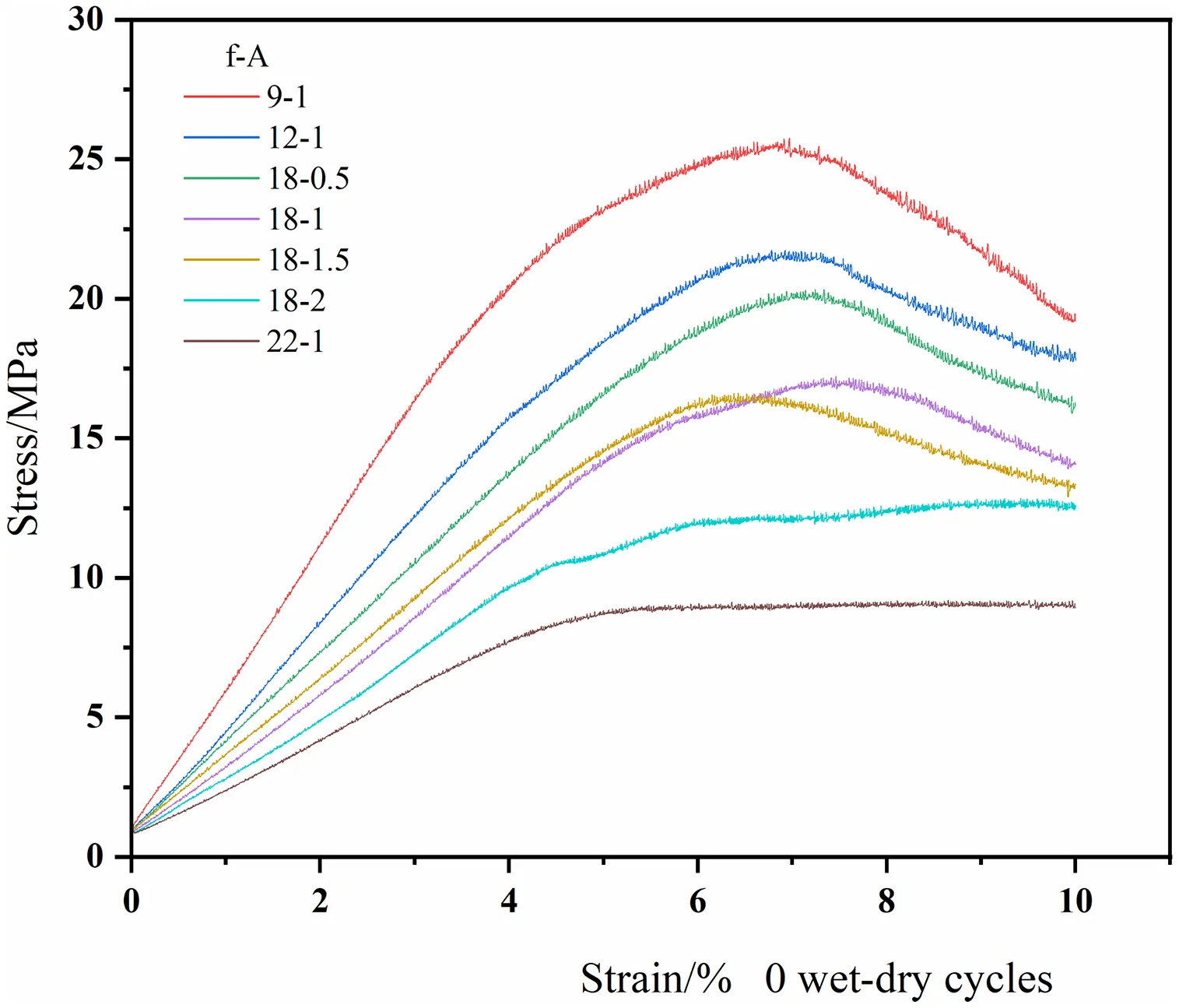
0 wetting-drying cycles.

**Fig 16:**
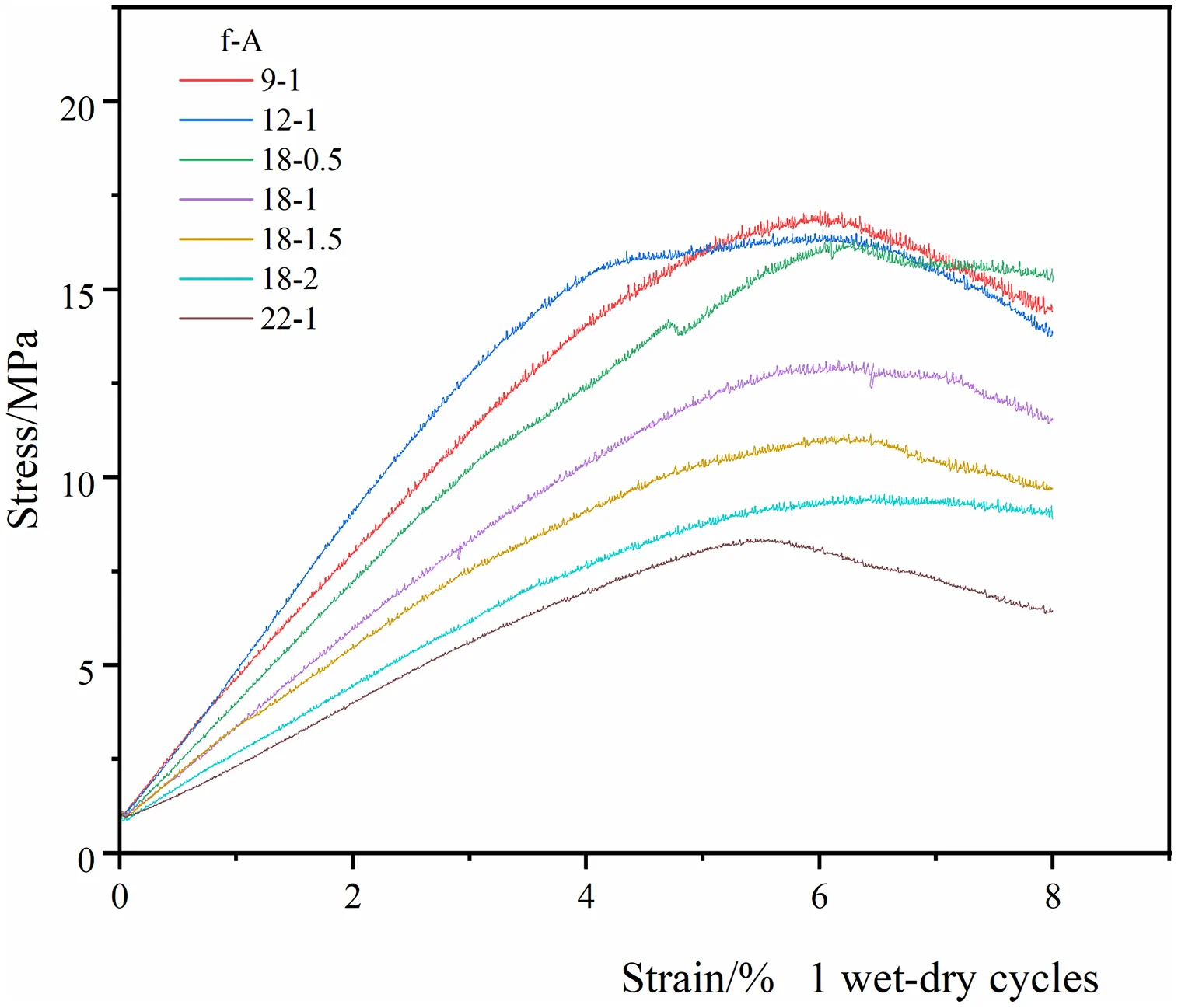
1 wetting-drying cycle.

**Fig 17:**
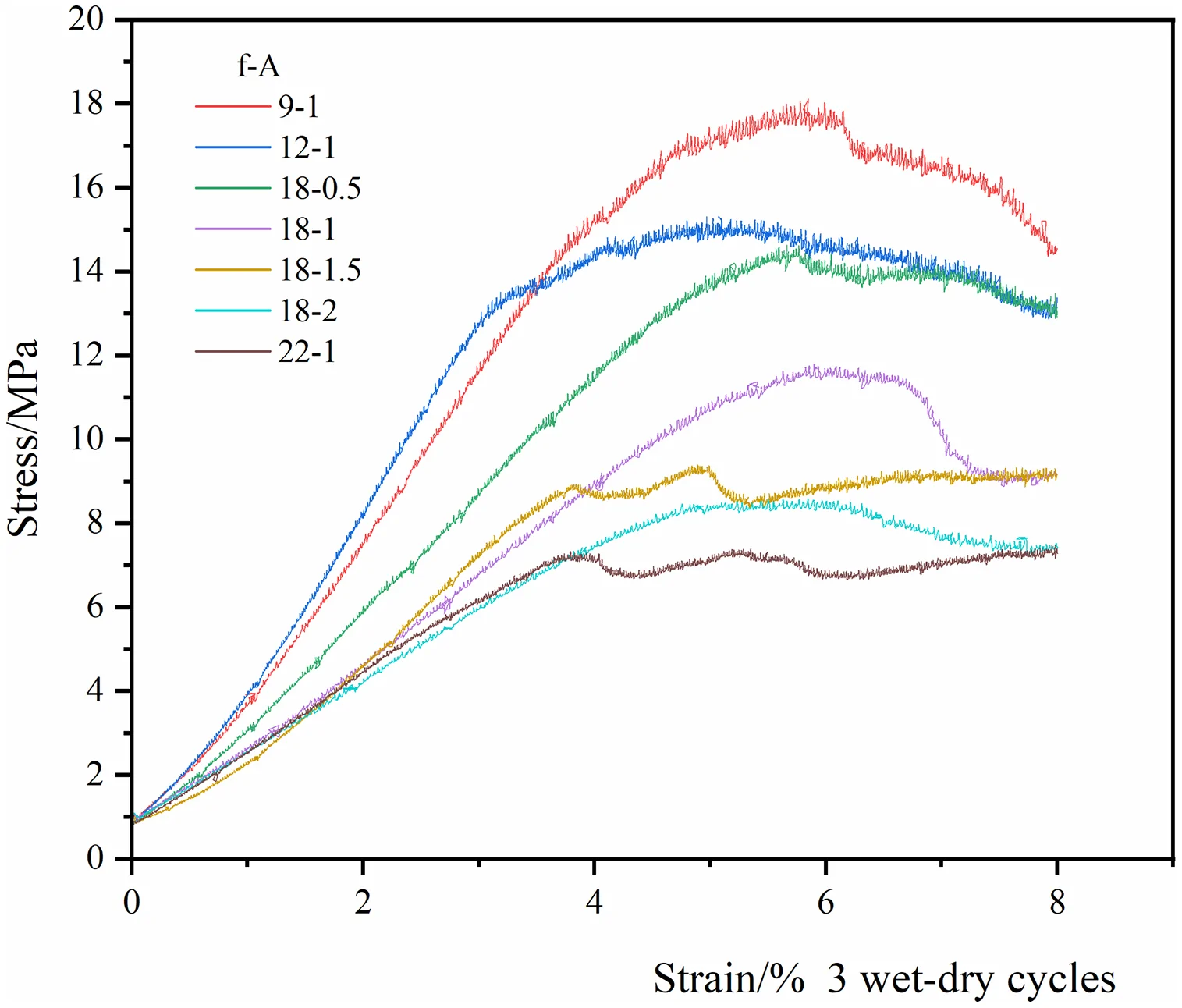
3 wetting-drying cycles.

**Fig 18:**
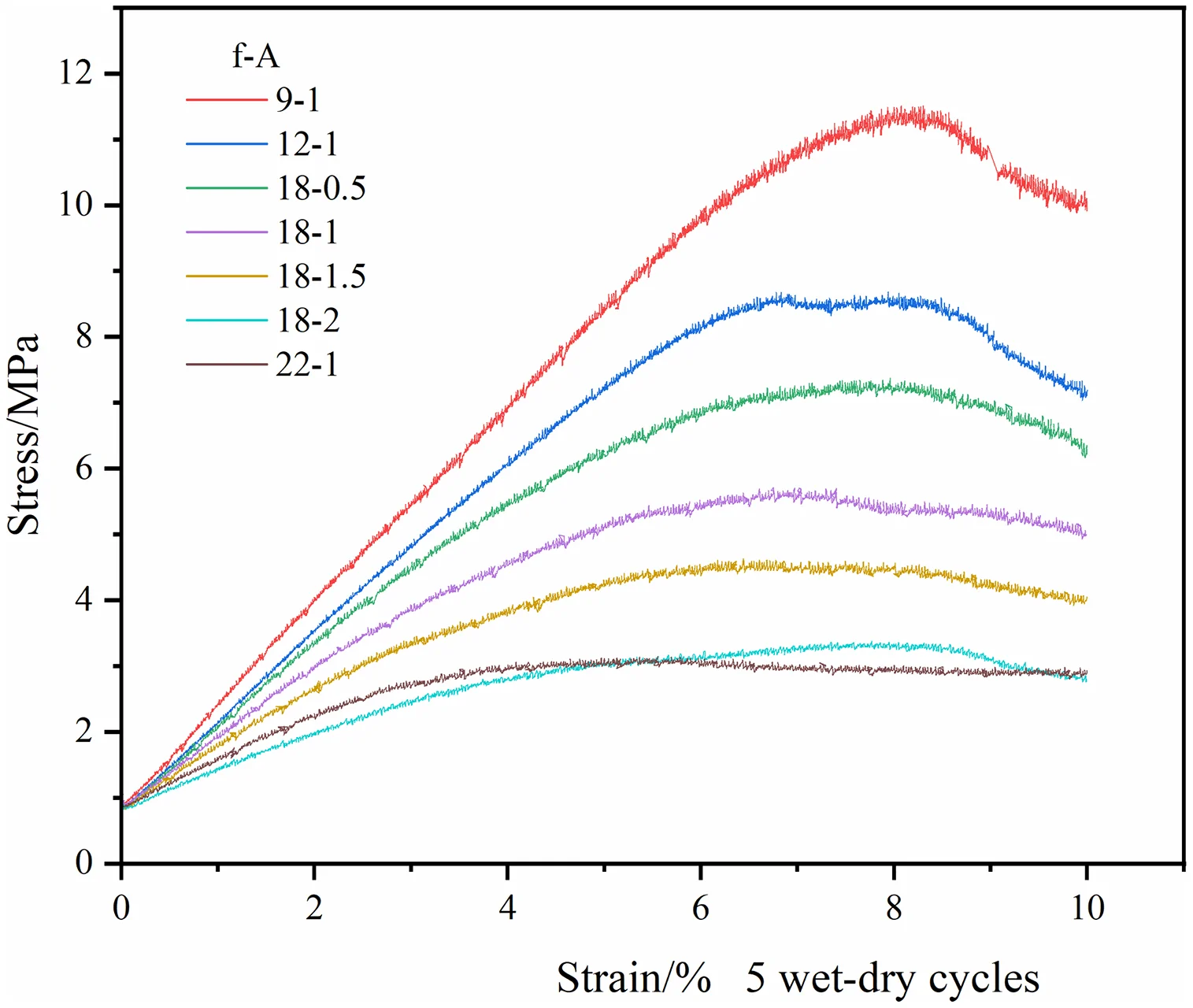
5 wetting-drying cycles.

**Fig 19:**
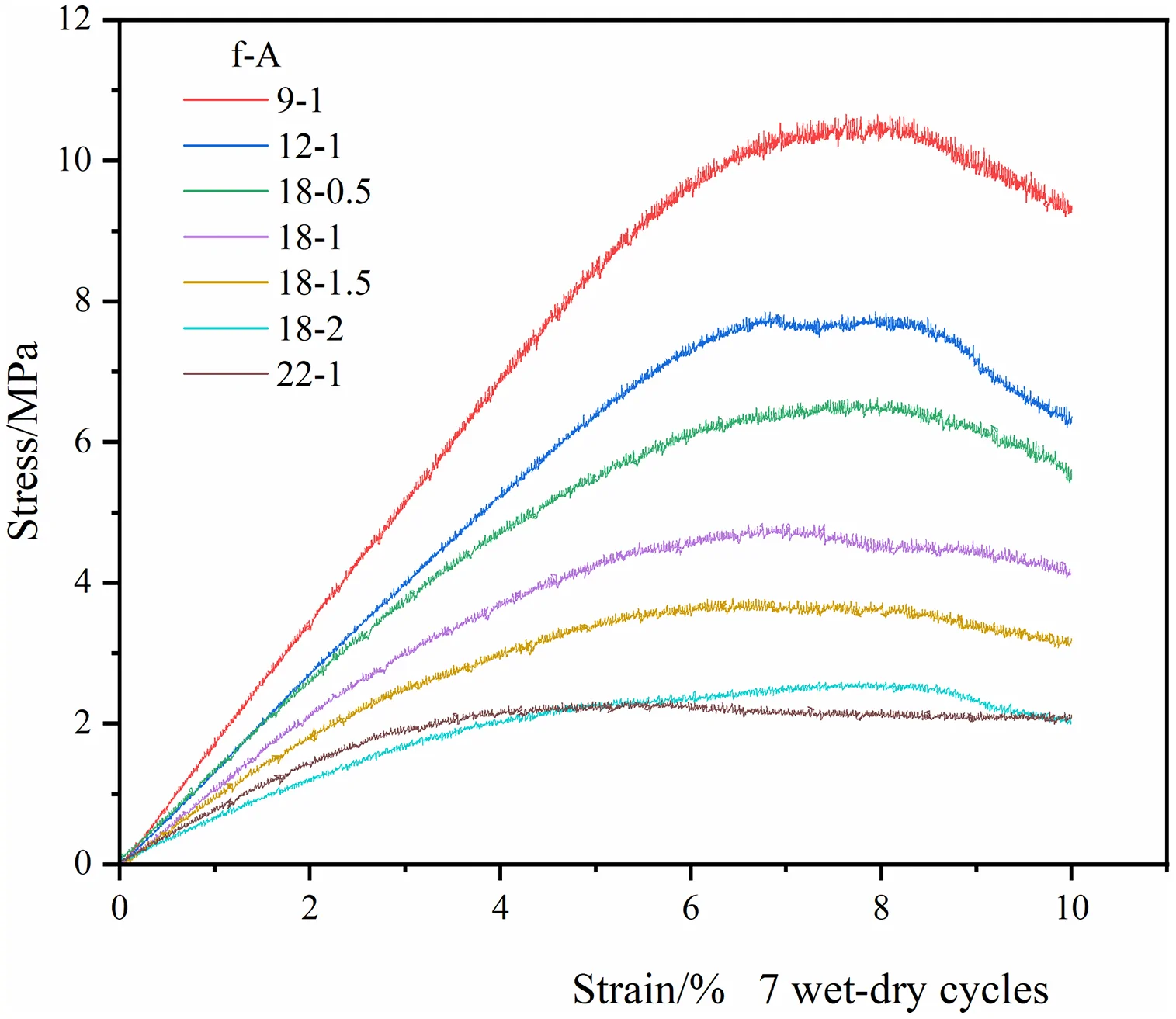
7 wetting-drying cycles.

Under the synergistic action of wetting-drying cycling and vibration loading, the peak strength of strongly weathered mudstone exhibits nonlinear degradation and cumulative damage that closely track the loss of deformation stiffness. In the overall evolution trajectory, the number of wetting-drying cycles defines the primary baseline for strength decay, while the introduction of vibration frequency and amplitude further catalyses deterioration. Along the frequency dimension, the peak strength decreases monotonically as the vibration frequency increases from 9 to 22 Hz at the same wetting-drying stage. This trend is especially pronounced after repeated wetting-drying alternation, such as 5 or 7 cycles, when frequent swelling and shrinkage of hydrophilic minerals has already generated abundant microcracks. Under these conditions, superimposed high-frequency vibration at 20–22 Hz produces a strong cumulative damage effect on load-bearing capacity, driving the peak strength rapidly to a minimum, for example to only 2.18 MPa after 7 cycles combined with 22 Hz loading.

Along the amplitude dimension, the response of peak strength to dynamic loading amplitude also shows clear stage dependence and damage dependence. At the early stage of wetting-drying cycling (0–1 cycles), the internal matrix skeleton remains relatively intact, and the strength reductions induced by amplitudes of 0.5–2.0 mm are comparatively uniform and gentle. However, as the number of cycles accumulates, the internal microstructure of the mudstone progressively loosens and softens, making the specimens increasingly sensitive to large amplitudes of 1.5–2.0 mm. As a result, macroscopic compressive strength collapses much faster under large-amplitude conditions at the middle and later stages. This coupled deterioration trend clearly shows that the initial micro-damage caused by alternating wetting and drying creates physical space for vibration-energy dissipation, whereas persistent high-frequency or large-amplitude disturbance directly accelerates the transition of internal cracks from accumulation to through-going coalescence, ultimately driving a comprehensive decline in mudstone mechanical performance.

### Three-dimensional deterioration trend of elastic modulus

Under the synergistic action of wetting-drying cycling and vibration loading, the mechanical behaviour of strongly weathered mudstone shows marked nonlinear degradation. Three-dimensional visualisation of key mechanical parameters, especially elastic modulus, enables a deeper analysis of the damage pattern induced by these environmental loads.

The three-dimensional surface in Fig 20 reveals the coupled deterioration of elastic modulus induced by vibration frequency and the number of wetting-drying cycles. At the same wetting-drying stage, the elastic modulus decreases monotonically as the vibration frequency rises from 9 to 22 Hz. This effect becomes especially clear after multiple wetting-drying alternations, when the surface in the high-frequency region (20–22 Hz) sinks markedly and the cool-coloured low-modulus area expands substantially. The result indicates that increasing frequency weakens rock stiffness cumulatively, and that this frequency sensitivity becomes more pronounced as matrix damage deepens, eventually leading to the minimum modulus under the combined condition of many cycles and high frequency.

**Fig 20:**
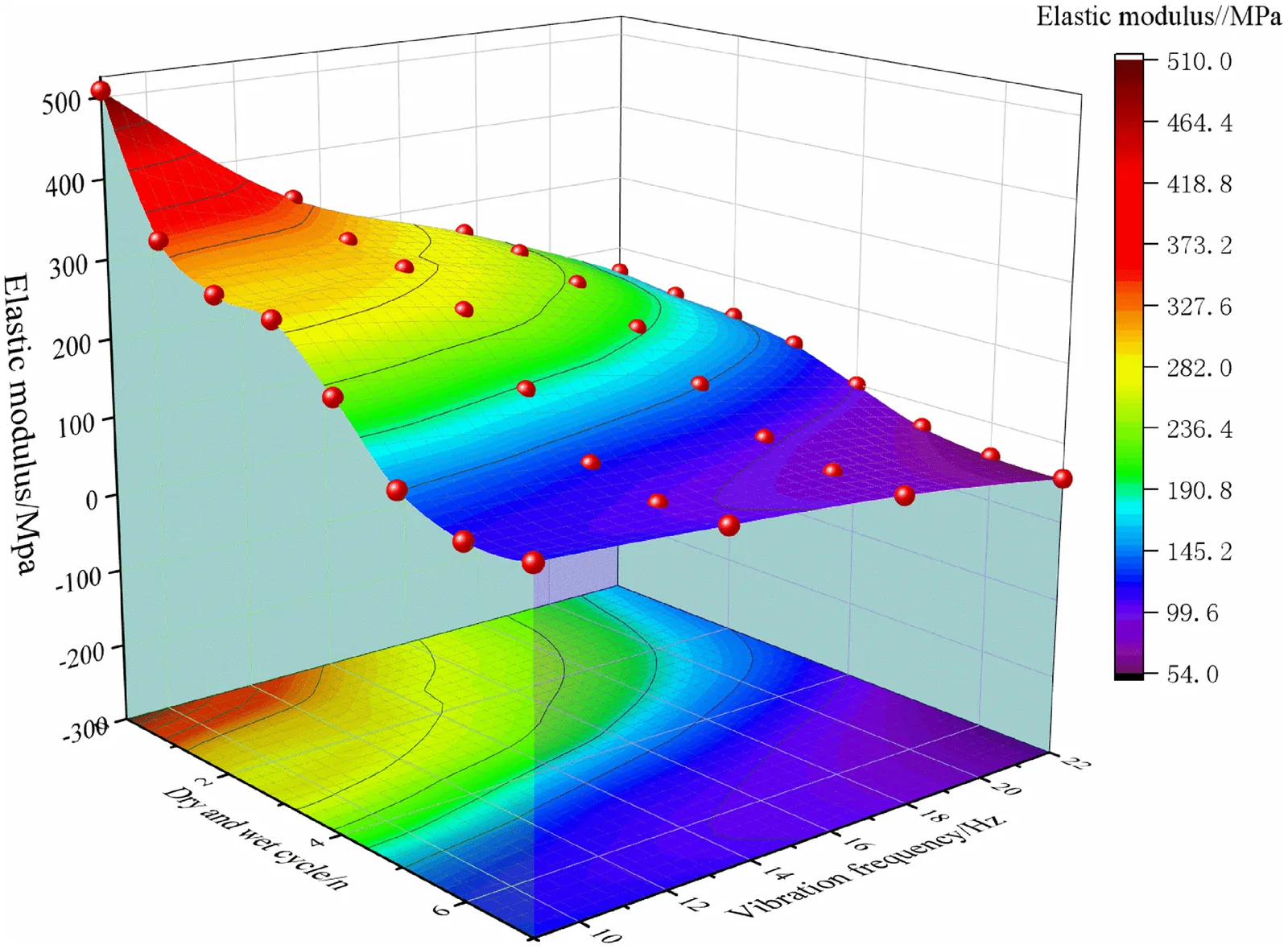
Number of wetting-drying cycles – vibration frequency – elastic modulus.

Fig 21 shows the nonlinear attenuation trajectory of elastic modulus with increasing wetting-drying cycles and vibration amplitude. Overall, the number of wetting-drying cycles is the dominant variable controlling modulus loss, and the surface drops steeply along the cycle axis. Along the vibration-amplitude dimension, the response of elastic modulus is strongly stage dependent. At the early stage, stiffness varies only mildly with amplitude. As the number of cycles increases, however, the internal structure of the mudstone becomes progressively looser, and the surface displays a clear downward collapse under large amplitudes of 1.5–2.0 mm. During the middle and late stages of physical damage evolution, the specimens become highly sensitive to large-amplitude dynamic disturbance, and stiffness loss is further intensified.

**Fig 21:**
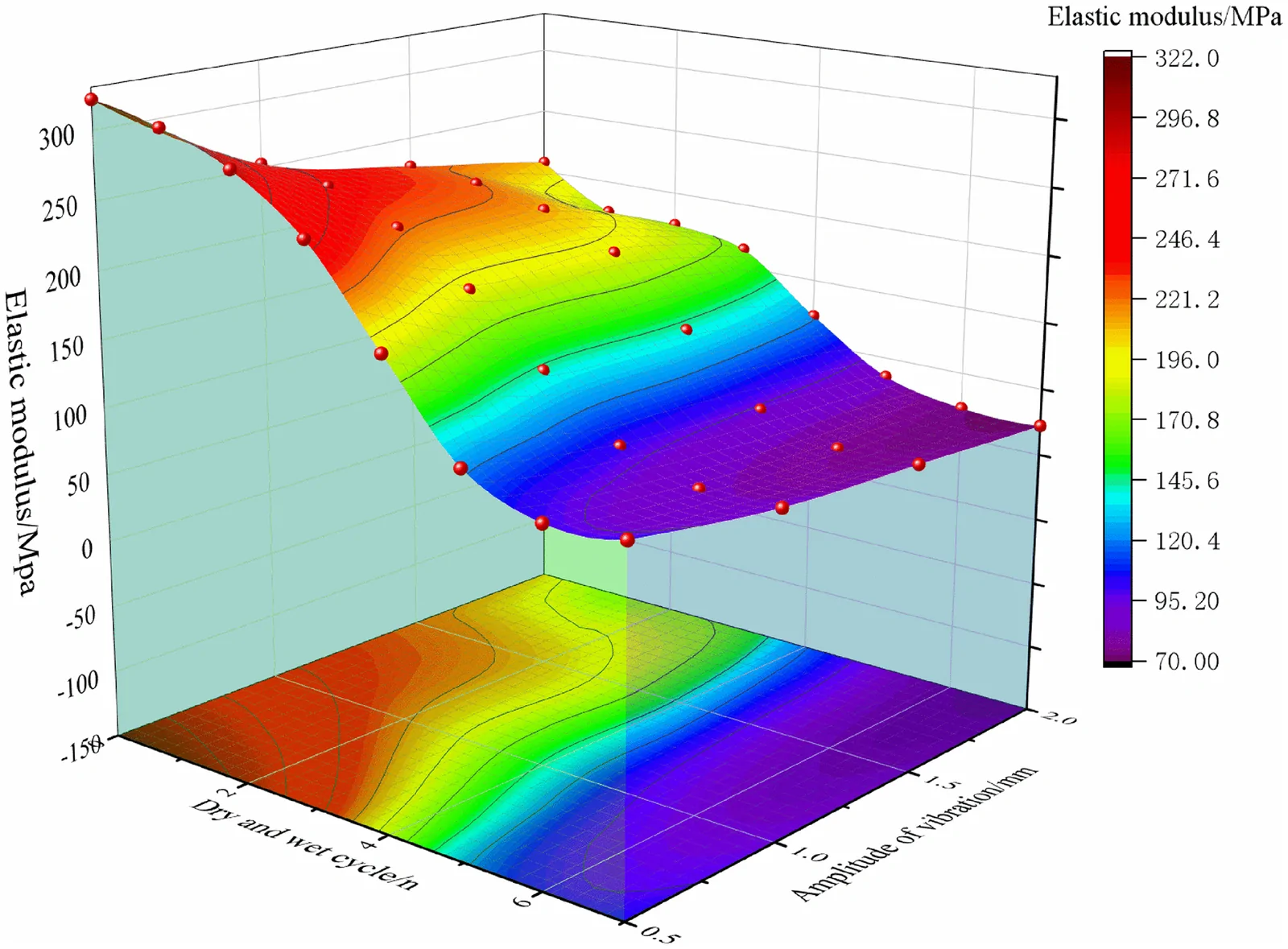
Number of wetting-drying cycles – vibration amplitude – elastic modulus.

### Analysis of ultrasonic propagation characteristics

The propagation of ultrasonic waves in rock and soil media is primarily controlled by internal structure, including the distribution and connectivity of pores, the development of fractures and the contact state between particles. To better understand the development of internal mudstone damage in complex environments and to evaluate mudstone-slope stability using non-destructive methods, this section focuses on conditions involving coupled dry, wet and vibration effects, and systematically analyses the evolution and underlying mechanisms of ultrasonic parameters, namely wave velocity, first-arrival amplitude and spectral centroid, under coupled loading. Table 3 summarises the acoustic data measured under different control conditions for subsequent analysis and comparison.

**Table 3:** Acoustic experimental data under different working conditions.

Condition No.	Number of wetting-drying cycles/n	Vibration frequency/f	Vibration amplitude/A	Strength/MPa	Elastic modulus/MPa	Wave velocity/km/s	First-arrival amplitude/dB	Spectral centroid/kHz
1	0 cycles	0 Hz	0 mm	27	510.13	1.696	142.53	50.29
2	0 cycles	9 Hz	1 mm	25.0398	508.35	1.689	138.53	49.75
3	0 cycles	12 Hz	1 mm	21.66544	374.63	1.603	136.58	49.52
4	0 cycles	18 Hz	0.5 mm	20.33109	321.49	1.563	130.98	46.98
5	0 cycles	18 Hz	1 mm	17.22438	252.85	1.420	128.30	45.61
6	0 cycles	18 Hz	1.5 mm	15.62342	227.56	1.389	125.37	45.47
7	0 cycles	18 Hz	2 mm	12.81388	207.43	1.359	123.42	44.53
8	0 cycles	22 Hz	1 mm	9.19788	165.39	1.330	120.96	44.4
9	1 cycle	0 Hz	0 mm	22.49	351.64	1.532	122.41	49.56
10	1 cycle	9 Hz	1 mm	19.90215	343.94	1.524	117.41	49.28
11	1 cycle	12 Hz	1 mm	16.47063	318.44	1.453	115.29	48.93
12	1 cycle	18 Hz	0.5 mm	16.31784	309.97	1.453	108.85	44.54
13	1 cycle	18 Hz	1 mm	13.10927	245.17	1.420	106.69	44.07
14	1 cycle	18 Hz	1.5 mm	11.16376	222.81	1.276	102.96	43.82
15	1 cycle	18 Hz	2 mm	9.55439	175.44	1.250	101.35	43.26
16	1 cycle	22 Hz	1 mm	8.36264	153.24	1.202	95.62	43.05
17	3 cycles	0 Hz	0 mm	18.49	300.64	1.210	116.14	47.94
18	3 cycles	9 Hz	1 mm	17.12075	292.35	1.157	115.14	47.97
19	3 cycles	12 Hz	1 mm	15.31299	270.67	1.136	110.91	45.26
20	3 cycles	18 Hz	0.5 mm	13.62698	252.7	1.116	106.82	43.46
21	3 cycles	18 Hz	1 mm	11.79545	189.5	1.042	105.37	41.16
22	3 cycles	18 Hz	1.5 mm	9.39141	188.89	1.025	100.96	40.90
23	3 cycles	18 Hz	2 mm	8.58673	164.42	1.016	99.98	40.70
24	3 cycles	22 Hz	1 mm	7.3746	130.25	0.992	90.94	40.46
25	5 cycles	0 Hz	0 mm	11.54	152.31	1.059	115.34	46.76
26	5 cycles	9 Hz	1 mm	11.33046	146.04	1.042	113.34	45.93
27	5 cycles	12 Hz	1 mm	8.6682	133.43	1.025	108.7	43.58
28	5 cycles	18 Hz	0.5 mm	7.19064	123.08	1.016	105	43.17
29	5 cycles	18 Hz	1 mm	5.70411	101.77	0.992	99.39	40.39
30	5 cycles	18 Hz	1.5 mm	4.52	93.34	0.977	98.67	40.15
31	5 cycles	18 Hz	2 mm	3.36135	85.45	0.868	94.5	39.79
32	5 cycles	22 Hz	1 mm	3.05962	68.32	0.791	86.67	39.26
33	7 cycles	0 Hz	0 mm	10.51	129.35	1.013	105.35	44.07
34	7 cycles	9 Hz	1 mm	10.45	124.13	0.919	99.35	43.57
35	7 cycles	12 Hz	1 mm	7.83	113.42	0.856	95.21	43.22
36	7 cycles	18 Hz	0.5 mm	6.57	104.62	0.833	90.08	40.73
37	7 cycles	18 Hz	1 mm	4.75	86.5	0.766	86.69	40.07
38	7 cycles	18 Hz	1.5 mm	3.73	79.34	0.653	83.78	39.83
39	7 cycles	18 Hz	2 mm	2.44	72.15	0.586	77.43	39.59
40	7 cycles	22 Hz	1 mm	2.18	55.67	0.538	71.84	38.86

### Time-domain propagation characteristics in strongly weathered mudstone

Time-domain waveforms provide the most direct record of ultrasonic propagation in strongly weathered mudstone and contain rich information on the evolution of internal structure. Fig 22–Fig 25 compares received waveforms under representative conditions to reveal, from the time-domain perspective, how multi-field coupling affects propagation path and energy attenuation. By comparing first-arrival waveforms in the initial state, under wetting-drying cycling alone and under coupled vibration, the Fig clearly shows the arrival-time delay and amplitude attenuation caused by the development of pores and fractures, thereby providing direct waveform evidence for the deterioration of strongly weathered mudstone from a dense to a loose internal state.

**Fig 22:**
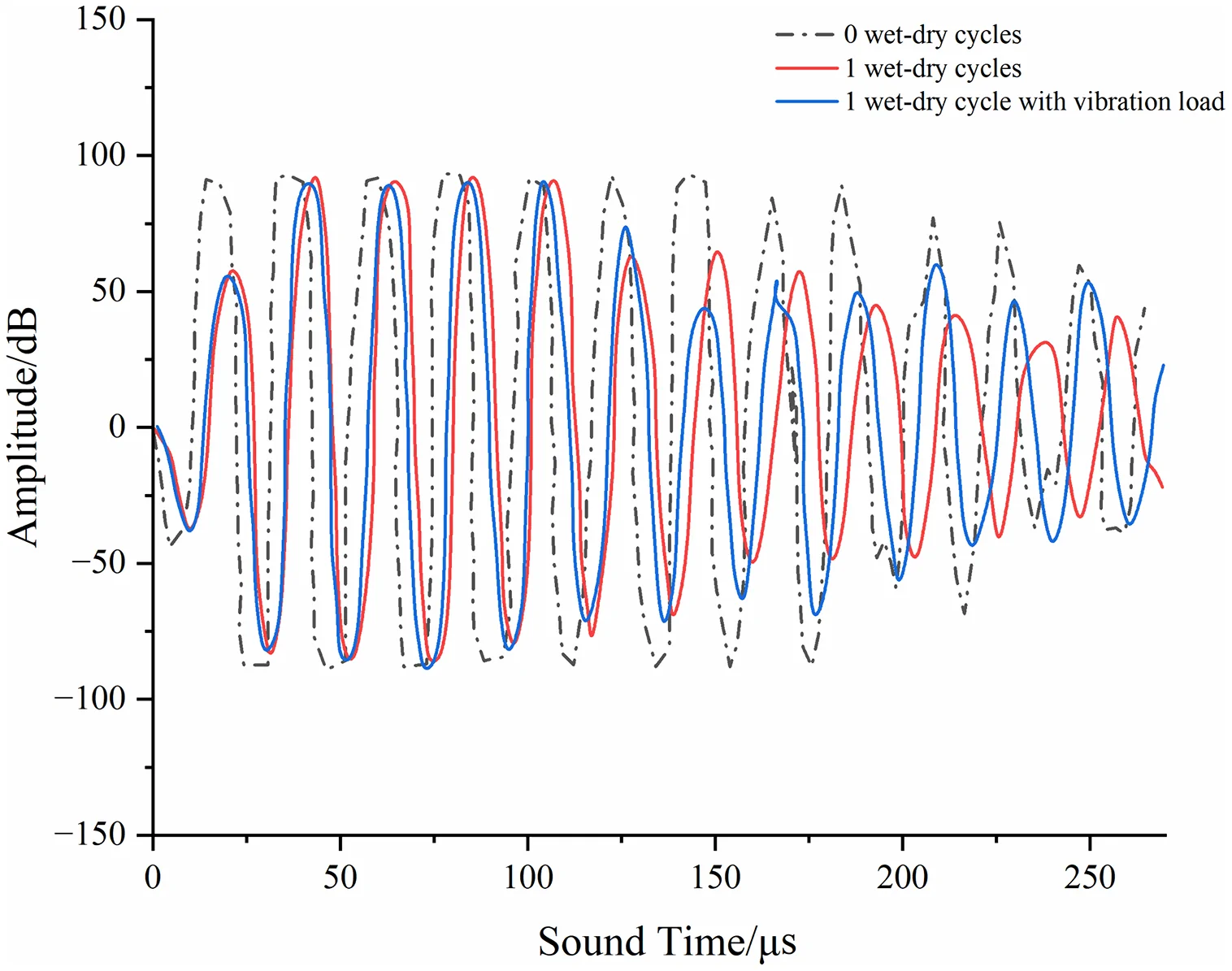
Ultrasonic time-domain waveform response after 1 wetting-drying cycle with and without vibration loading.

**Fig 23:**
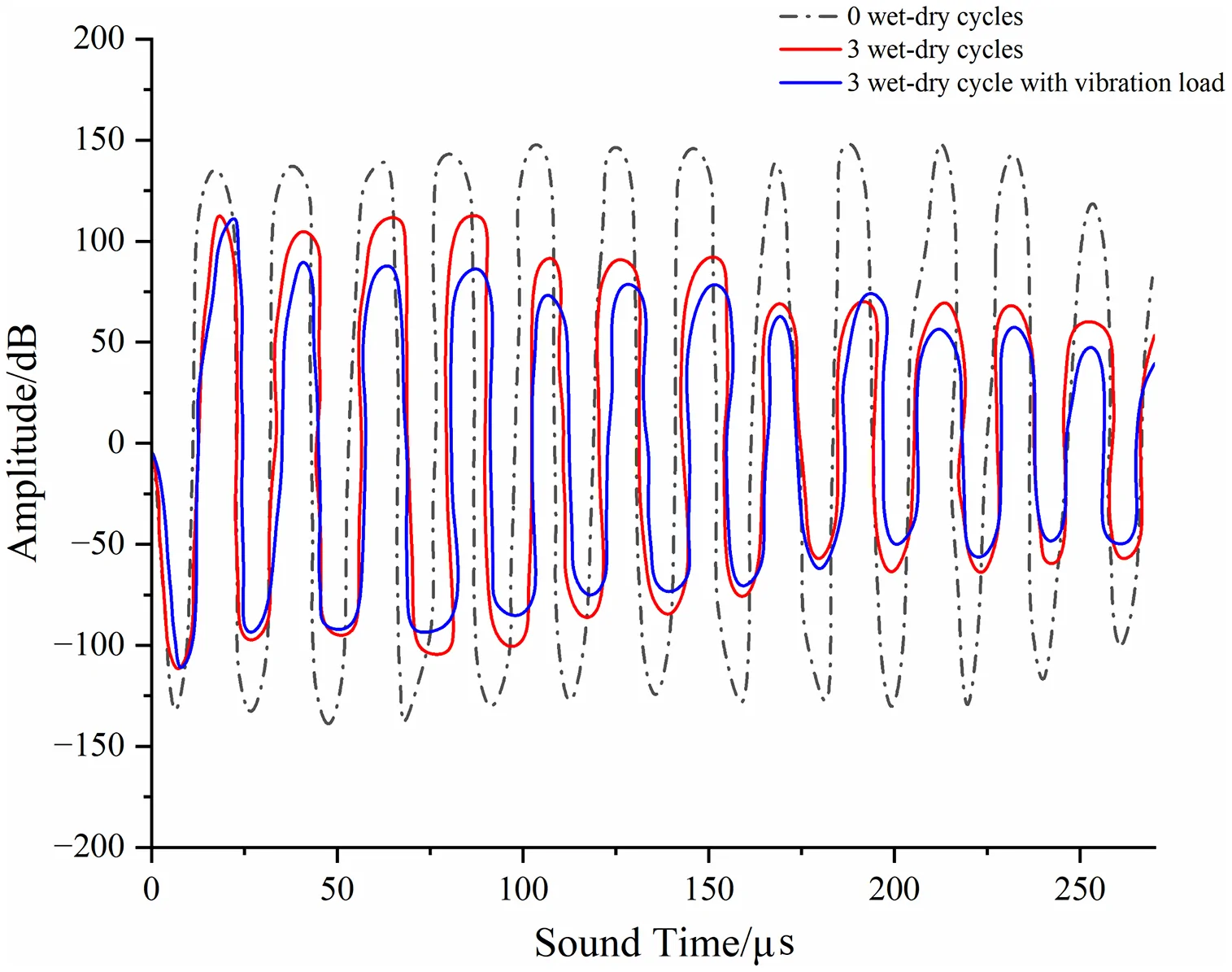
Ultrasonic time-domain waveform response after 3 wetting-drying cycle with and without vibration loading.

**Fig 24:**
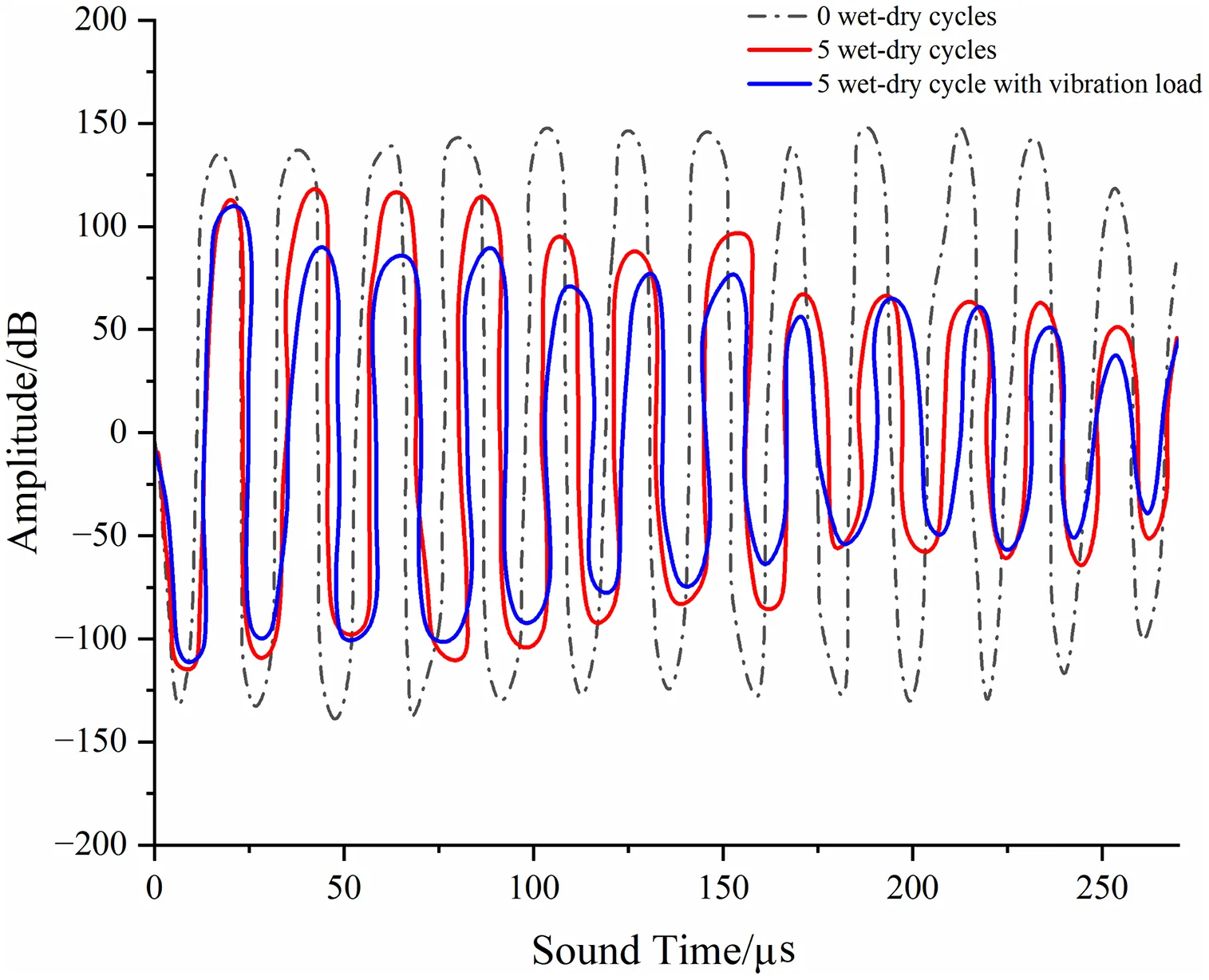
Ultrasonic time-domain waveform response after 5 wetting-drying cycle with and without vibration loading.

**Fig 25:**
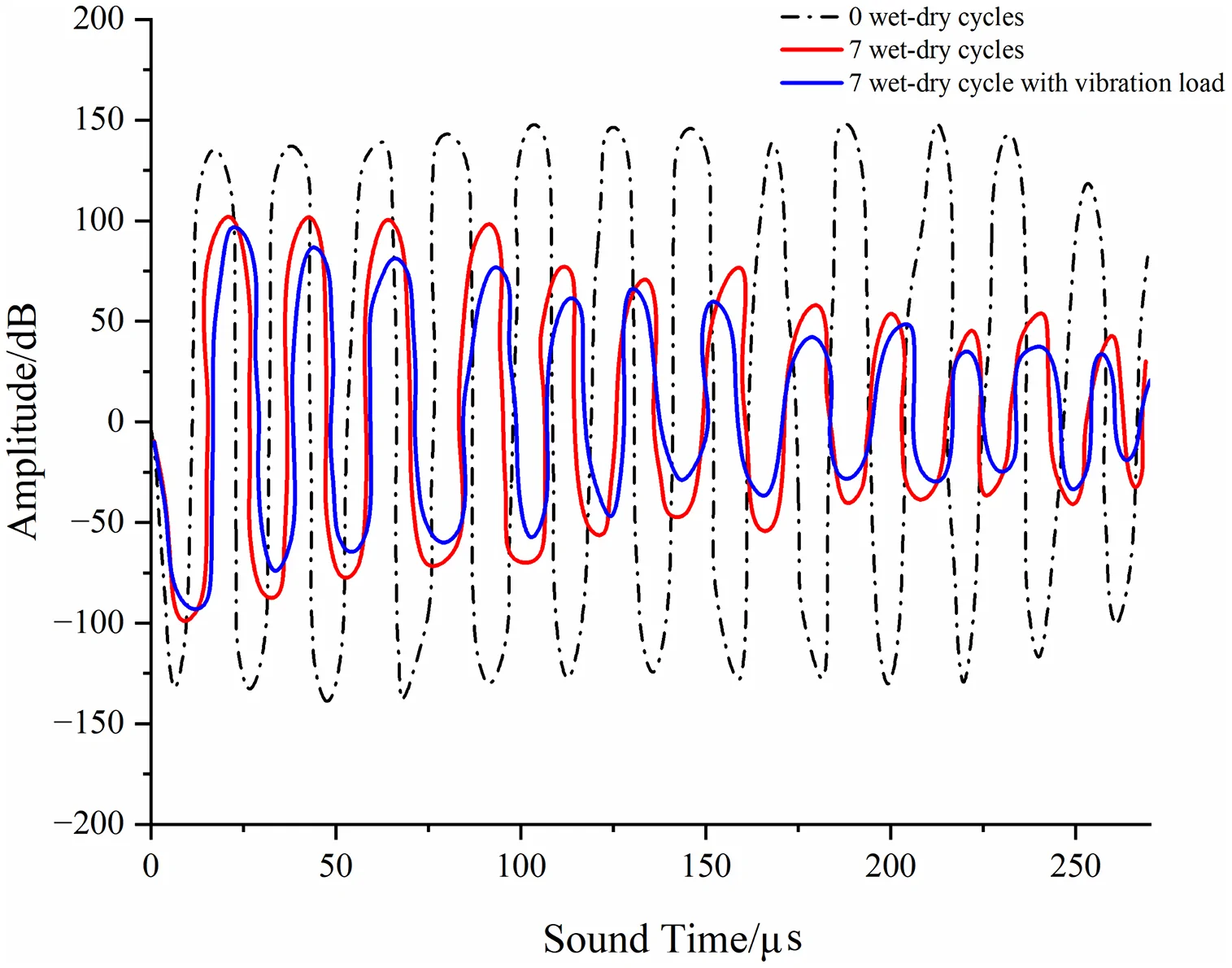
Ultrasonic time-domain waveform response after 7 wetting-drying cycle with and without vibration loading.

Comparison of the baseline waveform at 0 wetting-drying cycles with the ultrasonic responses after 1, 3, 5 and 7 cycles, with and without superimposed vibration loading, shows a clear evolution towards longer arrival times and lower amplitudes. The undamaged specimen at 0 cycles exhibits a tall and steep waveform, the shortest travel time and the highest first-arrival amplitude, indicating tight contact between mineral particles, no hydration swelling of clay minerals, a relatively straight wave path and low energy loss. Once wetting-drying cycling is introduced, the mechanical structure is disrupted. As the number of cycles increases, hydrophilic clay minerals generate cumulative microcracks, the waveform collapses towards the lower right, the first-arrival amplitude decays in a stepwise manner, the onset point shifts rightwards and the arrival time increases markedly. On this basis, vibration loading further catalyses and amplifies the trend of increasing arrival time and decreasing amplitude. At the same wetting-drying stage, the waveform under superimposed vibration is lower than that under wetting-drying cycling alone, and the difference widens as the number of cycles increases.

Equations (1) and (2) are the core expressions used to analyse time-domain attenuation, namely the classical definitions of logarithmic decrement and damping ratio. When ultrasonic waves propagate through strongly weathered mudstone containing internal microcracks, energy dissipation accelerates and waveform amplitude decays rapidly; these two expressions therefore provide a quantitative description of the associated viscoelastic deterioration process.

(1) Logarithmic decrement: used to measure the decay in peak amplitude between adjacent peaks or between peaks separated by n cycles.

$$\begin{array}{c} \delta =\frac{\text{1}}{n}ln\left(\frac{A_{k}}{A_{k+n}}\right) \end{array}$$ (1)

Ak: amplitude of the Kth peak.Ak+n: amplitude of the (K + n)th peak. N: number of cycles between the two peaks.

(2) Damping ratio: used to quantify the viscoelasticity of the material. A larger damping ratio indicates more internal pores and fractures and faster energy dissipation.

$$\begin{array}{c} \xi =\frac{\delta }{\sqrt{4\pi^{\text{2}}+\delta^{\text{2}}}}\approx \frac{\delta }{2\pi }\text{\textbackslash{}hspace\{1em}\}\left(\text{\textbackslash{}hspace\{0.33em}\}\delta \ll 1\text{\textbackslash{}hspace\{0.33em}\}\right) \end{array}$$ (2)

The Table 4 below lists the calculated logarithmic decrement and corresponding damping ratio for each condition.

**Table 4:** Logarithmic decrement and damping ratio.

Condition	Logarithmic decrement:δ	Damping ratio:ξ
0 wetting-drying cycles	0.0094	0.0015
1 wetting-drying cycle	0.0712	0.0113
1 wetting-drying cycle + vibration	0.1161	0.0185
3 wetting-drying cycles	0.1056	0.0168
3 wetting-drying cycles + vibration	0.1521	0.0242
5 wetting-drying cycles	0.1210	0.0193
5 wetting-drying cycles + vibration	0.1670	0.0266
7 wetting-drying cycles	0.1355	0.0216
7 wetting-drying cycles + vibration	0.1857	0.0294

The damping ratio at 0 wetting-drying cycles is denoted by (ξ_0_); the damping ratio under wetting-drying cycling alone without vibration is denoted by (ξ_1_); and that under wetting-drying cycling plus vibration is denoted by (ξ_2_).

These calculations quantitatively reproduce the core theoretical conclusion:ξ_2_ ≥ ξ_1_ ≥ ξ_0_. They demonstrate that the damping characteristics, that is, the viscoelasticity of the material, govern the attenuation rate of ultrasonic waves. Wetting-drying cycling plus vibration loading degrades pore water and cementation within the material, increases damping and accelerates waveform attenuation.

### Frequency-domain propagation characteristics

To further explore the cumulative microstructural damage mechanism of strongly weathered mudstone, the ultrasonic signals under different conditions were processed using the fast Fourier transform (FFT), an efficient algorithm for computing the discrete Fourier transform (DFT), as shown in equation (3). Fig 26 and Fig 27 presents the relationship describing changes in first-arrival amplitude under different numbers of wetting-drying cycles and is intended to reveal the evolution of ultrasonic response under wetting-drying cycling alone.

**Fig 26:**
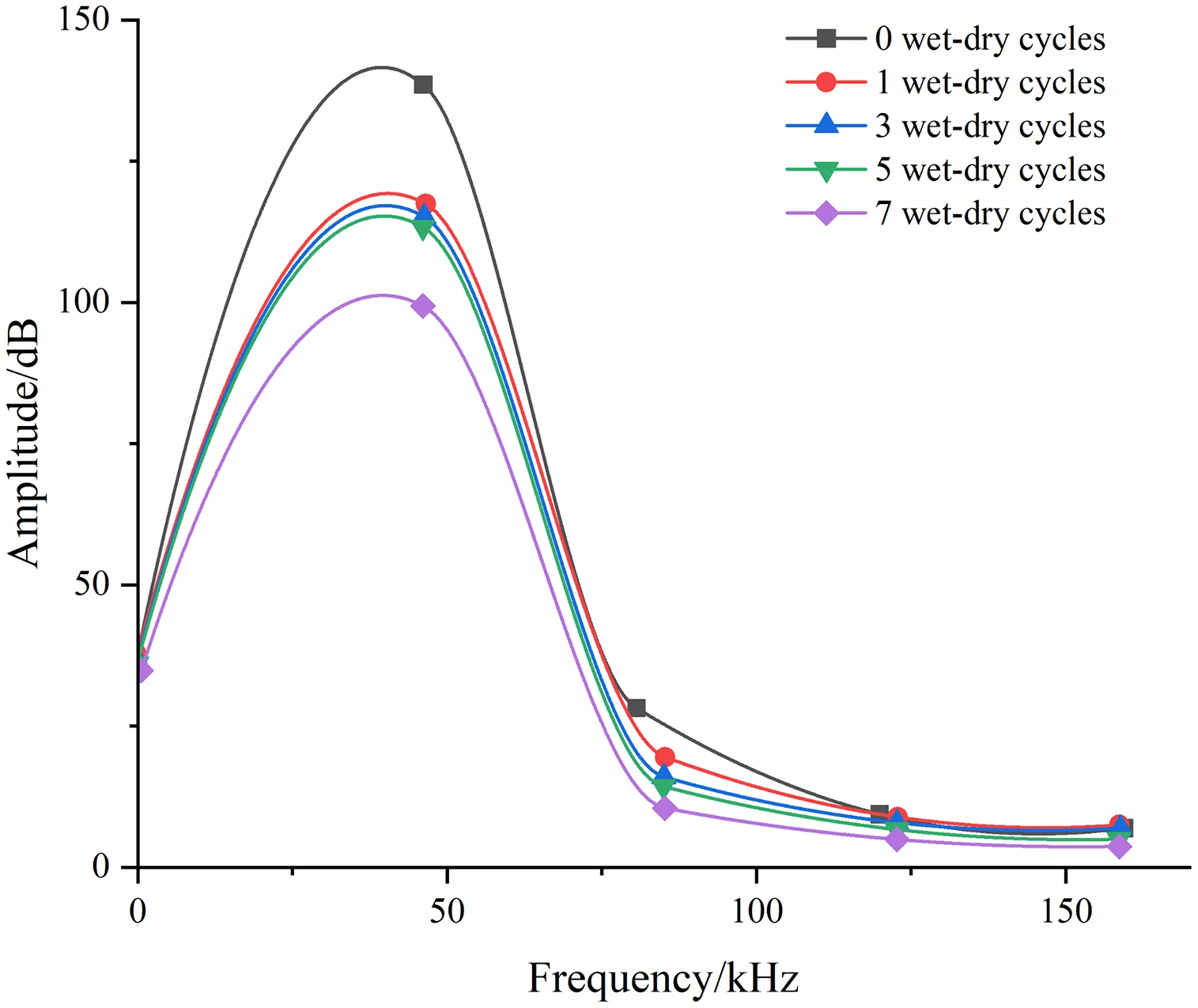
Frequency-amplitude spectra after wetting-drying cycling.

**Fig 27:**
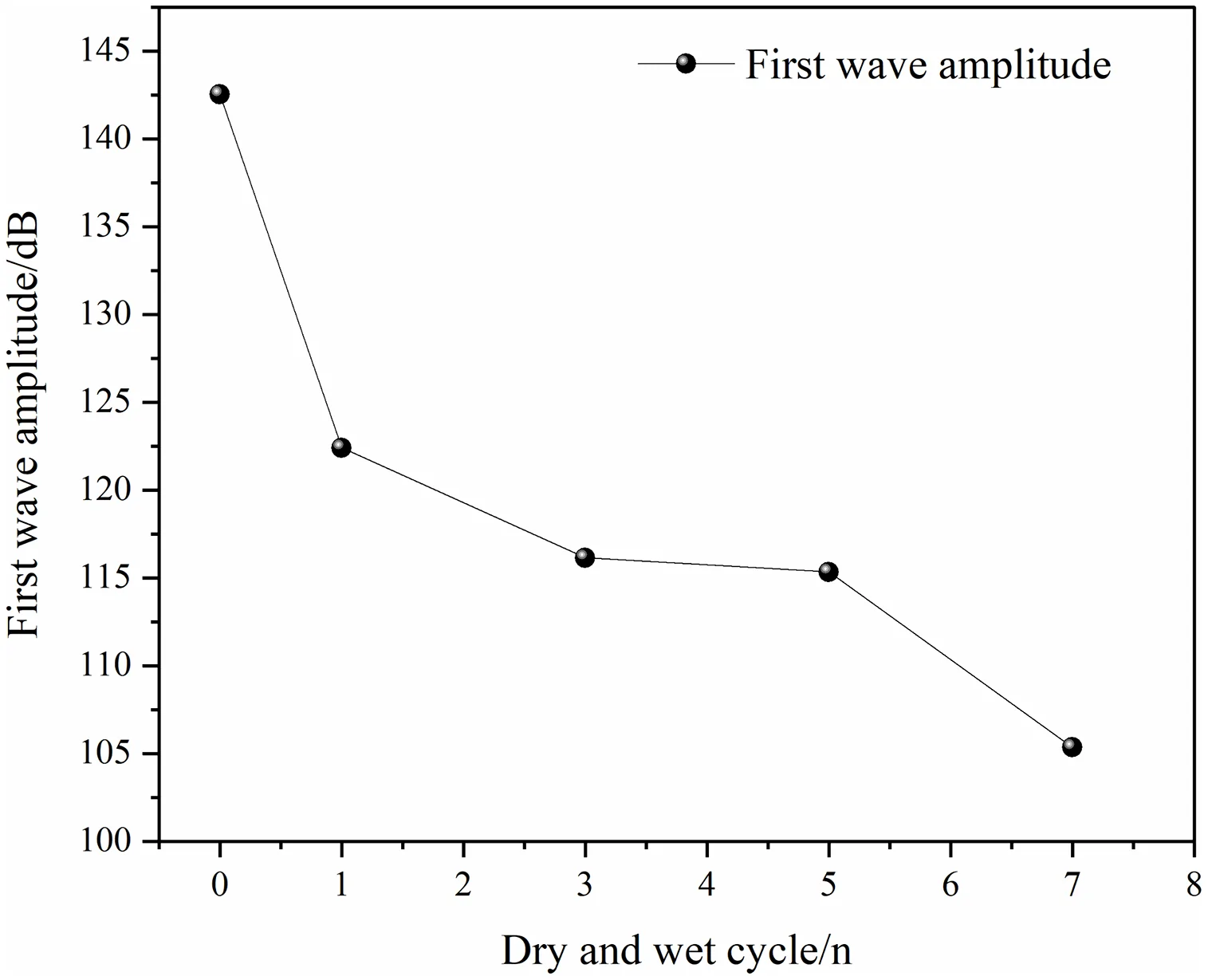
Variation in first-arrival amplitude under wetting-drying cycles.


$$\begin{array}{c} X\left[k\right]=\sum_{n=0}^{N-1}x\left[n\right]\cdot W_{N}^{kn},\text{\textbackslash{}hspace\{1em}\}k=0,1,2,\ldots ,N-1 \end{array}$$ (3)

Here, X_[k]_ is the transformed frequency-domain signal, k is the discrete frequency-domain index, x_[n]_ is the discrete time-domain waveform acquired by the ultrasonic instrument, W_n_ is the rotation factor, standard definition: Wn = e^-j2π/N^, N is the total number of sampling points, and j is the imaginary unit.

For specimens subjected only to wetting-drying cycling, the frequency-first-arrival-amplitude data are as follows: the first-arrival amplitudes after 0, 1, 3, 5 and 7 cycles were 142.53, 122.41, 116.14, 115.34 and 105.35 dB, respectively. Relative to the specimen without wetting-drying cycling, the corresponding reductions in first-arrival amplitude were 14.1%, 18.5%, 19.1% and 26.1%.

### Coupled effect of vibration loading and wetting-drying cycling

Figs 28–32 presents the frequency-domain curves obtained after superimposing vibration loading at different wetting-drying stages. The spectra directly record the frequency-domain response of ultrasonic signals after transmission through damaged mudstone, and mainly reflect the filtering effect of internal crack growth on high-frequency components and the dissipation of dominant-frequency energy under fatigue-type dynamic loading.

**Fig 28:**
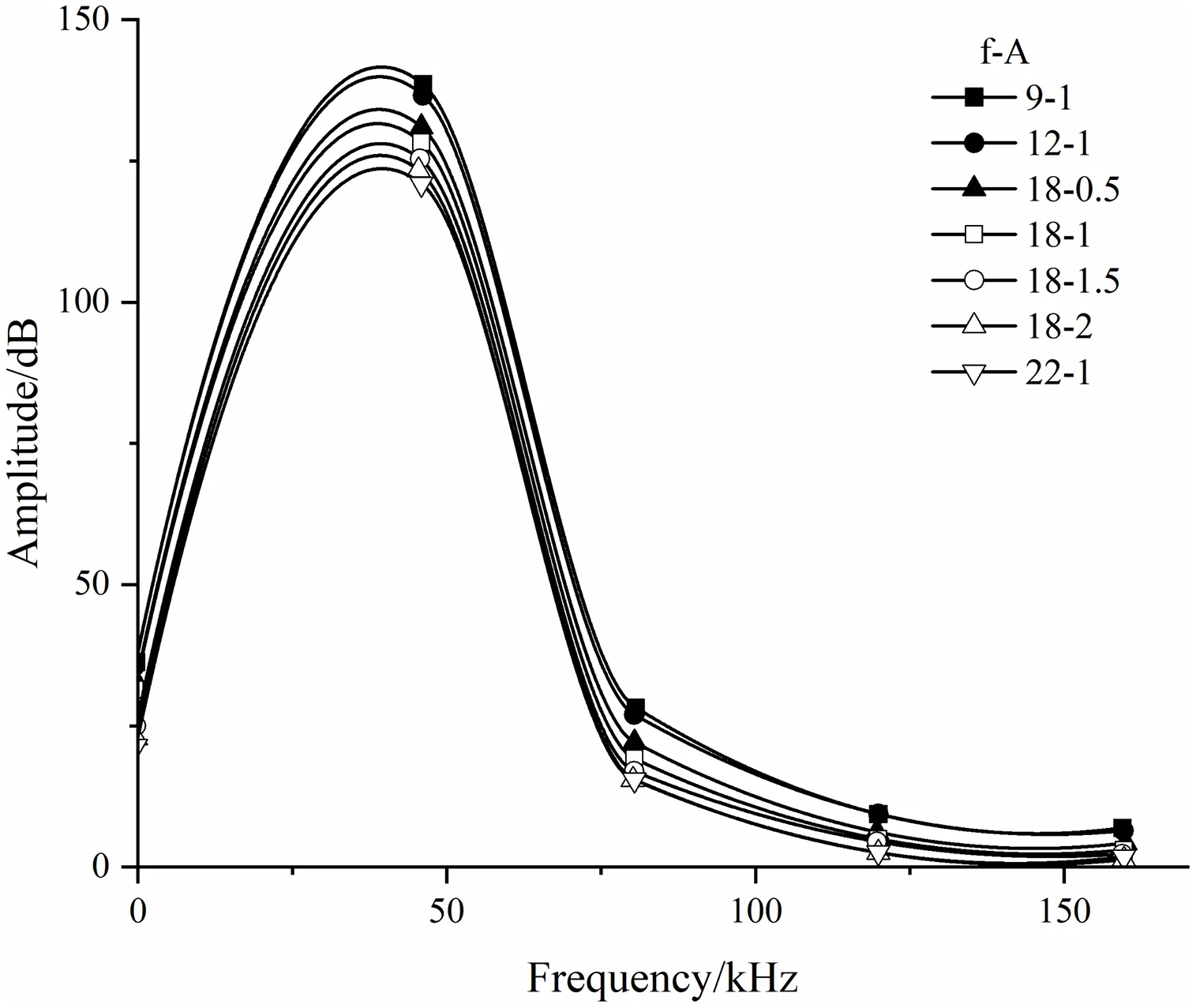
Frequency-amplitude spectra for different conditions after 0 wetting-drying cycles.

**Fig 29:**
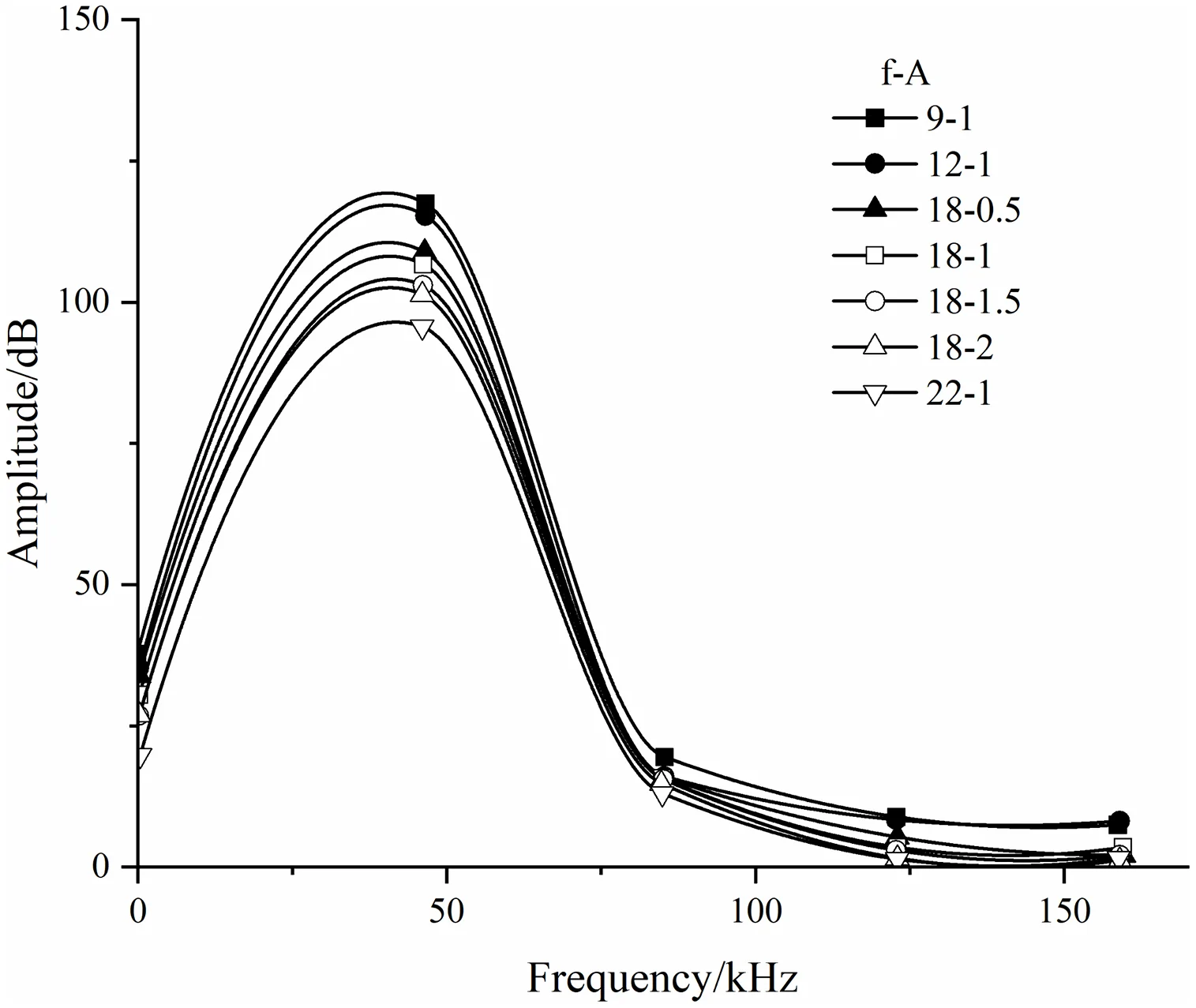
Frequency-amplitude spectra for different conditions after 1 wetting-drying cycle.

**Fig 30:**
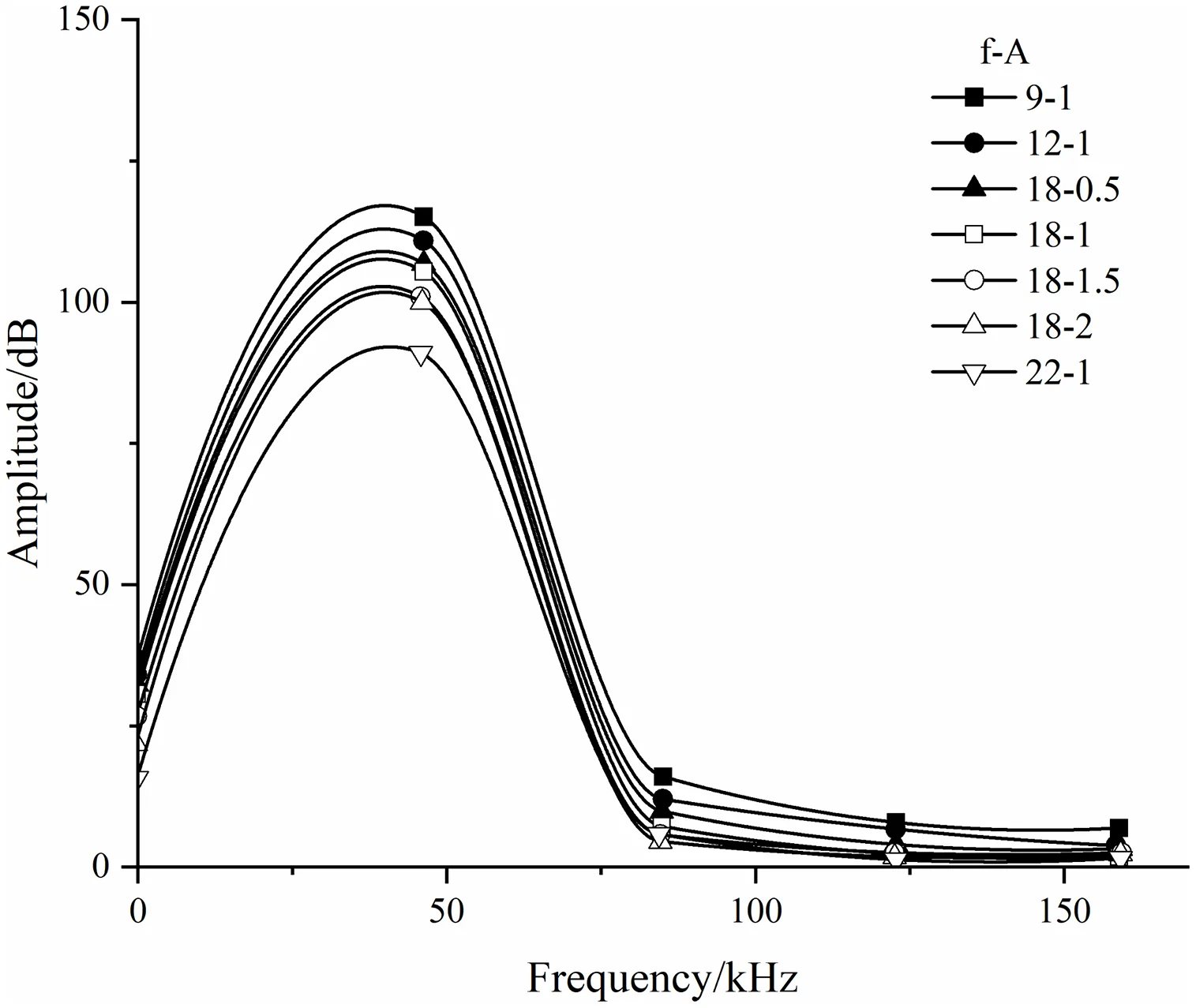
Frequency-amplitude spectra for different conditions after 3 wetting-drying cycles.

**Fig 31:**
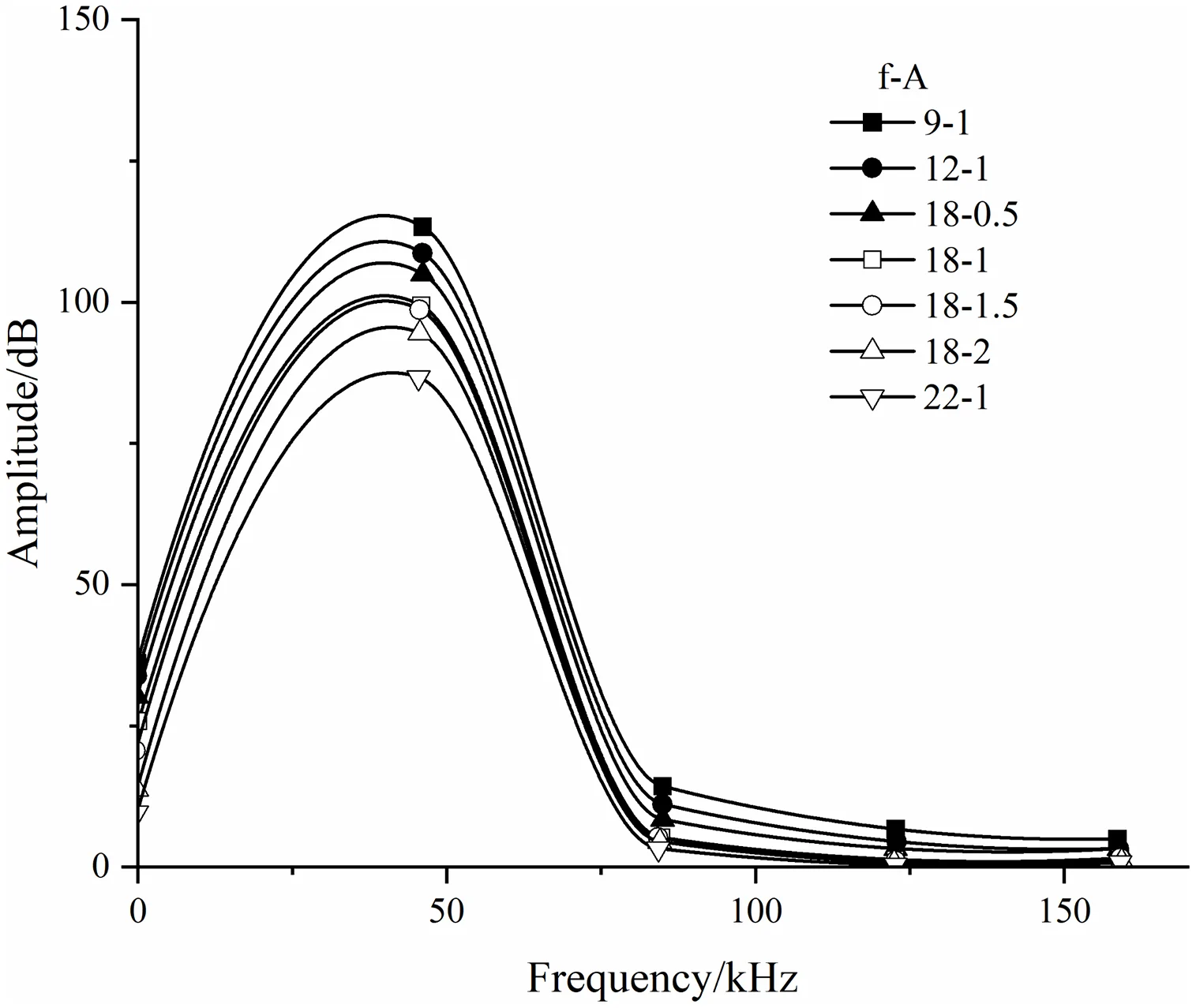
Frequency-amplitude spectra for different conditions after 5 wetting-drying cycles.

**Fig 32:**
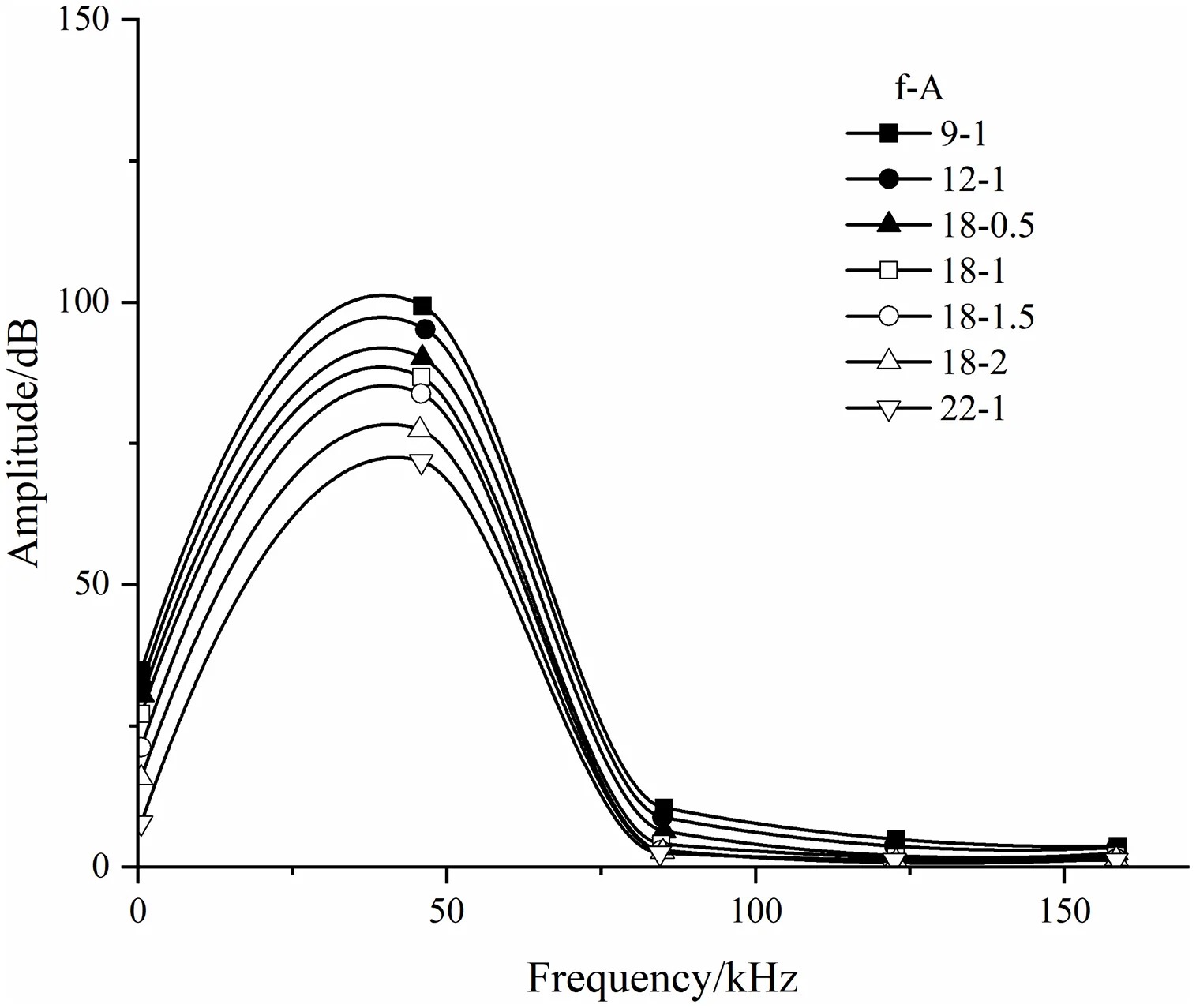
Frequency-amplitude spectra for different conditions after 7 wetting-drying cycles.

Compared with the results for wetting-drying cycling alone, superimposed vibration under the same number of cycles causes a further marked decline in the frequency-amplitude response of the ultrasonic signal. Taking the one-cycle condition as an example, the peak first-arrival amplitude under wetting-drying cycling alone was 122.41 dB, whereas it decayed further under superimposed vibration; under the strong disturbance condition of 18 Hz and 2 mm, it dropped to only 101.35 dB, a decrease of 17.2% relative to the wetting-drying-only state. This finding indicates a pronounced synergistic deterioration effect between vibration disturbance and wetting-drying cycling, such that the initiation and propagation of internal microcracks in the mudstone occur much faster than under either factor alone.

### Correlation between spectral centroid and mechanical properties

The spectral centroid can be regarded as the centre of gravity of the frequency spectrum. Its calculation is given in equation (4), and the parameter can map the internal microstructural state of strongly weathered mudstone. When the internal structure is intact, high-frequency components are abundant and the centroid frequency is high. As damage intensifies and high-frequency components attenuate rapidly, the centroid frequency shifts towards lower frequencies. The evolution of spectral centroid can therefore sensitively and quantitatively characterise changes in the mechanical properties of specimens subjected to coupled wetting-drying and vibration loading.


$$\begin{array}{c} f_{c}=\frac{\sum_{i=1}^{n}A_{i}f_{i}}{\sum_{i=1}^{n}A_{i}} \end{array}$$ (4)

In equation (4), is the spectral centroid of the ultrasonic signal, in kHz, and represents the amplitude spectrum corresponding to frequency.

Fig 33 establishes a quantitative mapping between peak strength and ultrasonic spectral centroid for strongly weathered mudstone under multi-field coupled loading. The scatter distribution shows a pronounced and tight positive linear correlation, without obvious dispersion or disorder. The data cluster forms a clear inclined band along the fitted curve, revealing synchronous evolution between the ultimate load-bearing capacity of the mudstone and its acoustic frequency response. As the number of wetting-drying cycles accumulates and the vibration condition becomes more severe, the data points migrate directionally from the upper-right region of high strength and high spectral centroid, corresponding to a relatively intact structure, towards the lower-left region of low strength and low centroid. This pattern indicates that when internal structural deterioration causes progressive loss of high-frequency components in the ultrasonic signal and drives the centroid towards lower frequencies, peak strength decays in an approximately constant proportion. Notably, even in the low-strength residual-damage stage, the spectral centroid still responds sensitively to small strength fluctuations rather than saturating, confirming the effectiveness of this frequency-domain index for back-calculating macroscopic mudstone failure strength.

**Fig 33:**
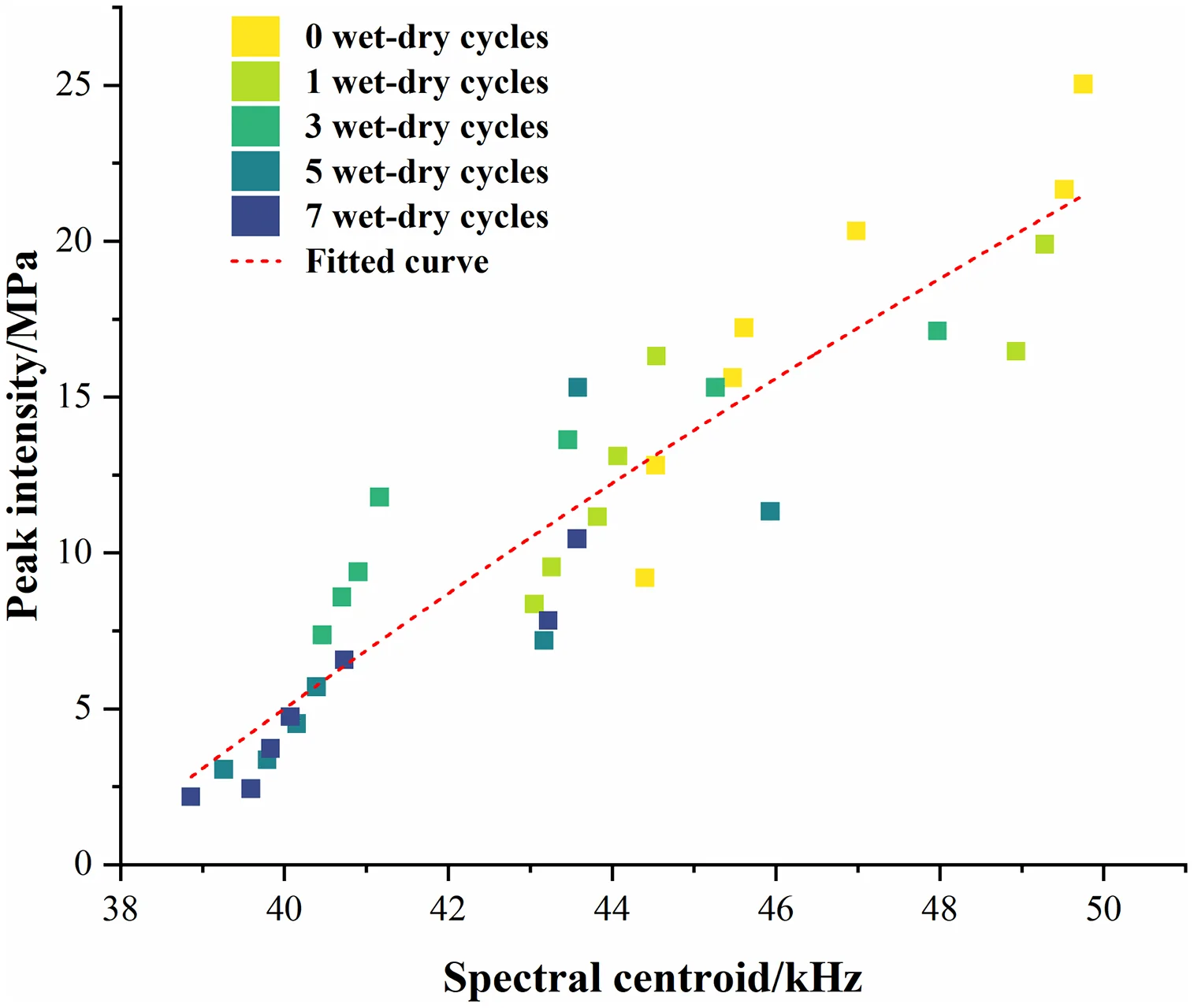
Spectral centroid versus peak strength.

Fig 34 further shows the relationship between elastic modulus and spectral centroid in strongly weathered mudstone, and its evolution closely resembles that of peak strength. The data points lie tightly along the fitted curve, clearly reflecting the intrinsic link between mudstone stiffness and frequency-domain response. Before wetting-drying cycling, the material retains high connective stiffness and the data cluster in the high-modulus, high-frequency region. As the matrix softens and damage accumulates, the elastic modulus drops sharply and the corresponding spectral centroid also decreases markedly.

**Fig 34:**
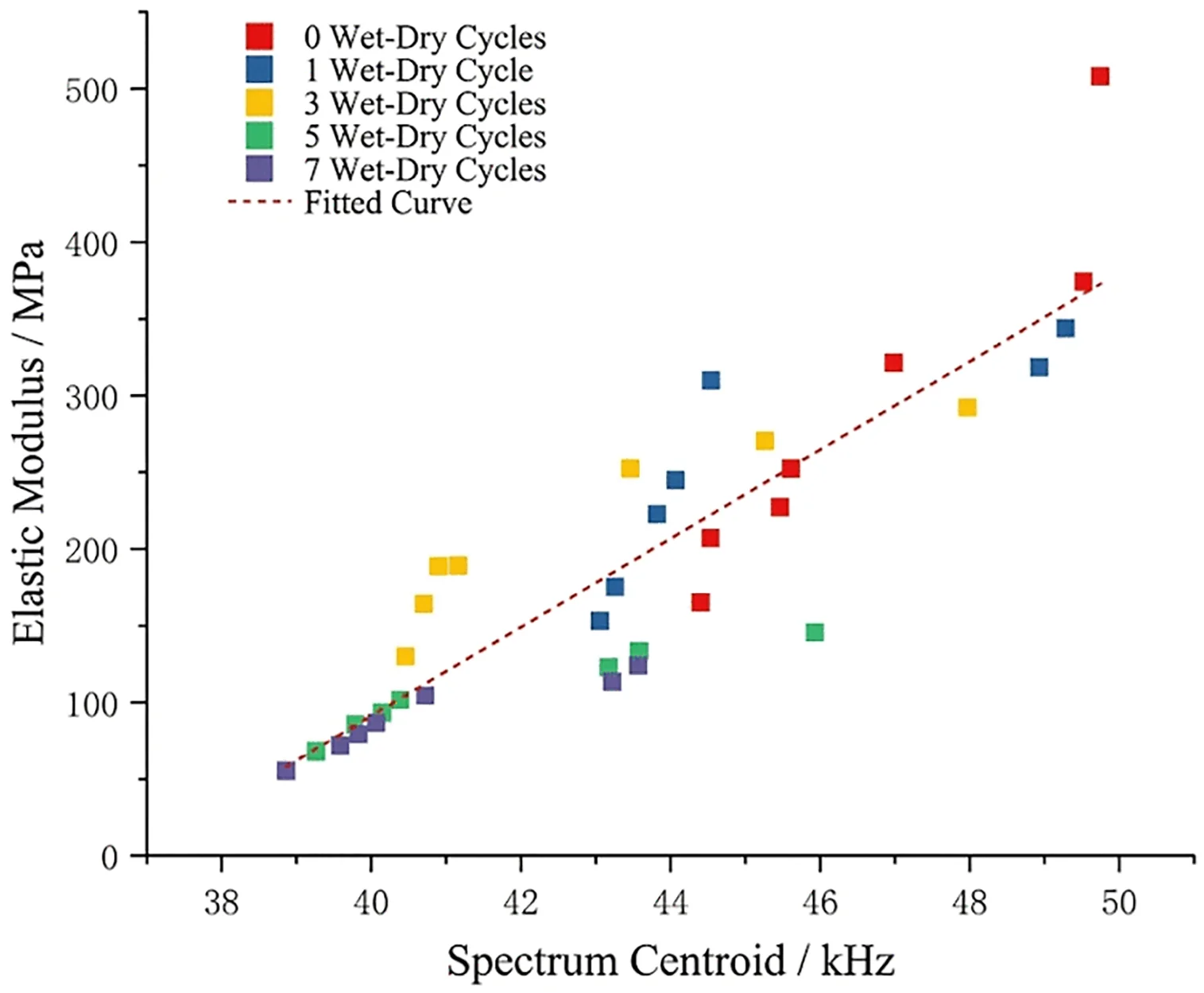
Spectral centroid versus elastic modulus.

### Variation in longitudinal wave velocity

The experimental results show that the longitudinal wave velocity (LWV) of strongly weathered mudstone is highly sensitive to environmental state.

Fig 35, composed of five vertically stacked subplots, clearly shows the evolution of longitudinal wave velocity versus peak strength under different numbers of wetting-drying cycles, ordered from top to bottom as 7, 5, 3, 1 and 0 cycles. Across the full set of panels, the data display a clear hierarchical structure and a positive correlation trend. In the bottom subplot for 0 cycles, the data cluster in the upper-right region, corresponding to the high-velocity range of 1.3–1.7 km/s and the high-strength range of 10–25 MPa, which directly reflects the favourable physical-mechanical state of the mudstone in its initial condition. Moving upwards through the subplots, that is, as the number of wetting-drying cycles increases, the data cloud shifts dramatically towards the lower left. By the top subplot at 7 cycles, velocity has fallen to the low range of 0.5–1.0 km/s, and peak strength has contracted to a residual level of 2–10 MPa. This synchronous attenuation trajectory shows that the reduction in wave velocity is the acoustic manifestation of increasing porosity and weakening cementation in strongly weathered mudstone, and that this microstructural damage inevitably appears at the macroscopic scale as loss of load-bearing capacity.

**Fig 35:**
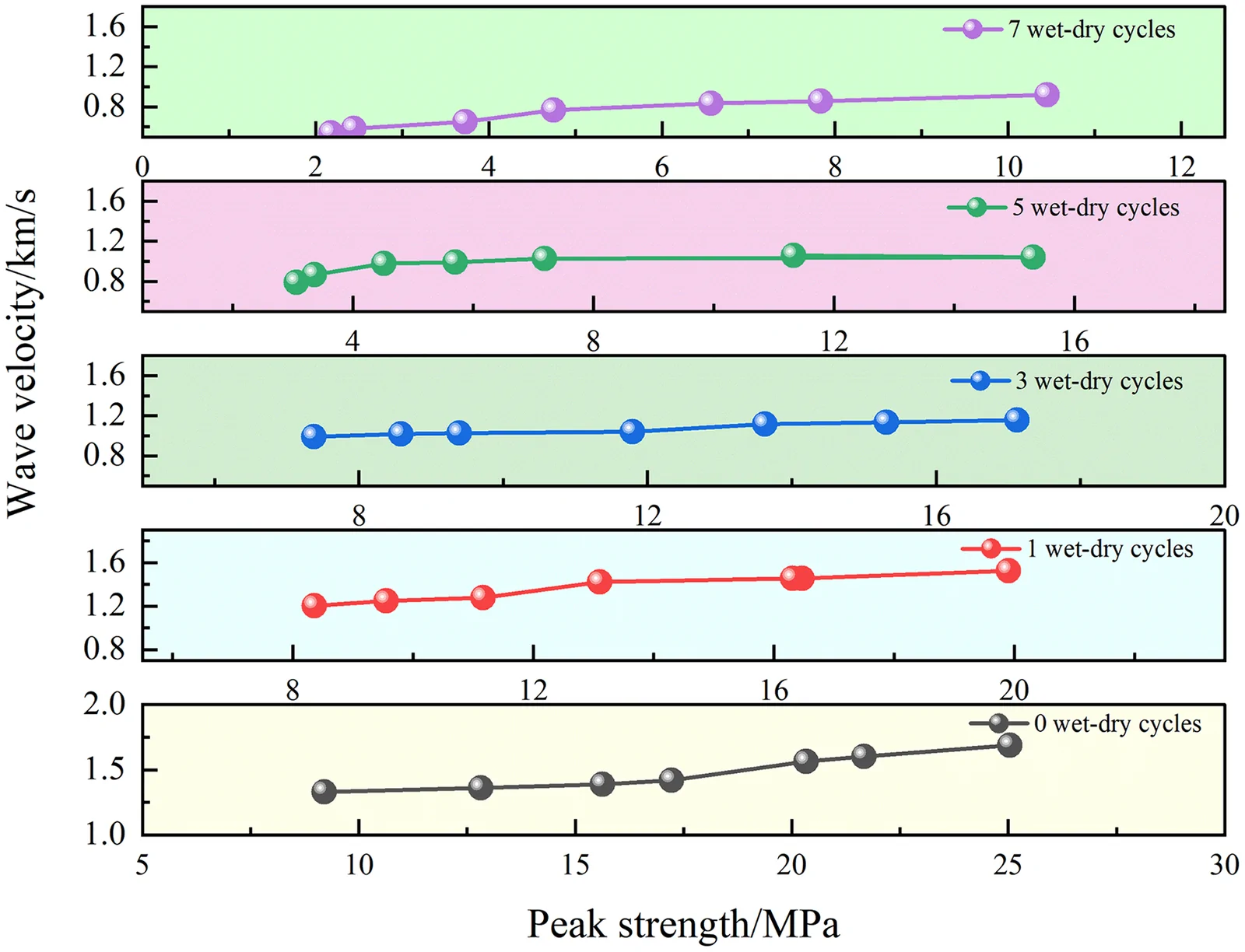
Peak strength versus wave velocity.

Fig 36 describes the relationship between longitudinal wave velocity and elastic modulus. Although the caption suggests a linear relation, the observed shape shows a highly characteristic nonlinear growth trend, which vividly reflects the physical connection between deformation stiffness and acoustic propagation speed in strongly weathered mudstone. The data are not scattered randomly, but instead align tightly along a clear trajectory. In the low-velocity range below 0.8 km/s, the elastic modulus remains low and increases only slowly, mostly staying below 100 MPa. At this stage the mudstone contains abundant weak clay minerals and free water, which strongly damp ultrasonic waves and provide little resistance to deformation. Once the wave velocity exceeds the threshold of 1.0 km/s, however, the elastic modulus rises in an almost exponential manner to 300–500 MPa. This steep segment corresponds to the relatively intact stage of the strongly weathered mudstone structure, such as after 0 or 1 cycle, when mineral particles remain tightly connected and the skeletal stiffness is high, so that even a small increase in densification, reflected in higher wave velocity, yields a large improvement in resistance to deformation.

**Fig 36:**
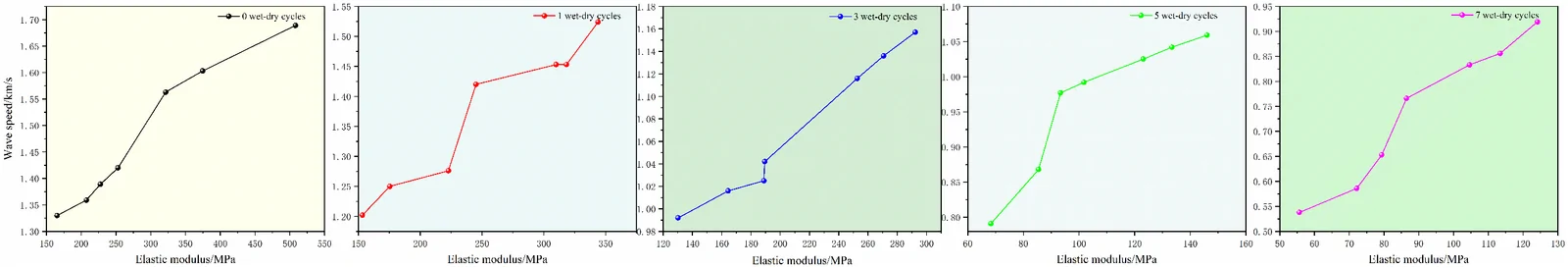
Elastic modulus versus wave velocity.

Taken together, these evolution trajectories reveal the physical essence of the stiffness-wave-velocity relationship: wave velocity is highly sensitive to the compactness of strongly weathered mudstone, whereas elastic modulus is the direct mechanical projection of that compactness. The differently coloured curves represent specific paths under different vibration conditions. Although their numerical values differ, their highly consistent overall trend indicates that, whether the damage is induced by wetting-drying cycling or vibration loading, the physical mechanism ultimately responsible for reducing wave velocity is synchronous.

### Weight analysis of the influence of vibration factors on strongly weathered mudstone

To guide the prioritisation of vibration-reduction strategies in engineering practice, achieve the best mitigation effect at the lowest economic cost and improve the cost-effectiveness of protective measures, it is necessary to clarify how vibration affects the internal structure of mudstone and to quantify the relative contributions of frequency and amplitude under coupled loading. We therefore establish a quantitative relationship between the vibration-disturbance coefficient and the damage factor to provide a basis for subsequent damage evaluation.

### Damage factor characterised by ultrasonic parameters (Spectral Centroid)

Because the spectral centroid represents the centre of gravity of spectral energy distribution, has a clear physical meaning, is highly sensitive to damage and is robust to noise in ultrasonic testing, we use the rate of change of spectral centroid as the damage factor for the weight analysis, thereby balancing physical interpretability and detection sensitivity, as shown in equation (5).


$$\begin{array}{c} D_{f}=\frac{F_{w0}-F_{wfA}}{F_{w0}} \end{array}$$ (5)

In this expression, D_f_ is the damage factor represented by spectral centroid. The value F_w0_ is the reference indicator for an unvibrated specimen at the same number of wetting-drying cycles, and F_wfA_ is the indicator for the corresponding specimen after vibration loading. This factor removes the background damage introduced by wetting-drying cycling and allows the analysis to focus on the independent contribution of vibration factors (Figs 37–41).

**Fig 37:**
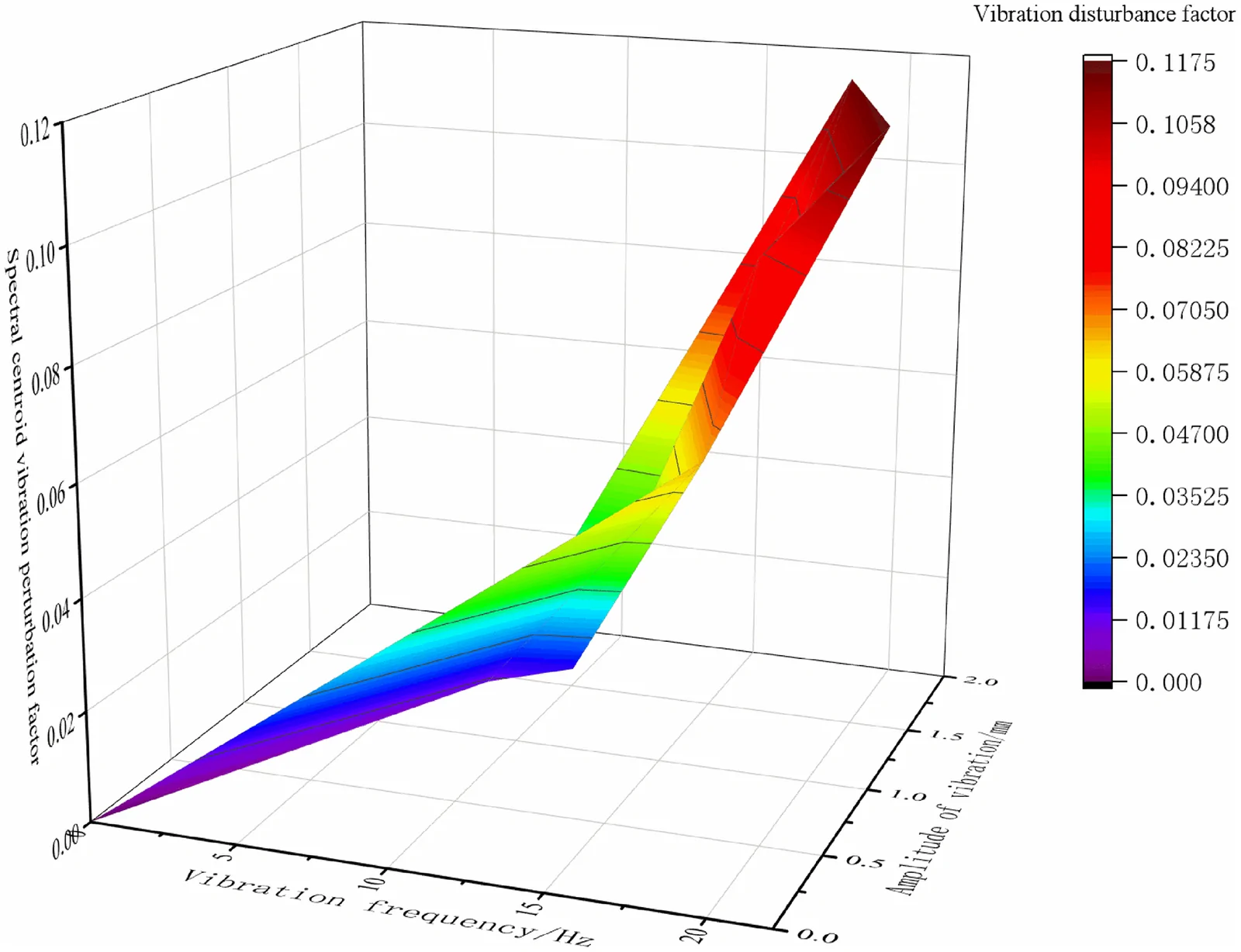
Changes in vibration frequency, vibration amplitude, and spectral centroid vibration disturbance factor under 0 wet-dry cycles.

**Fig 38:**
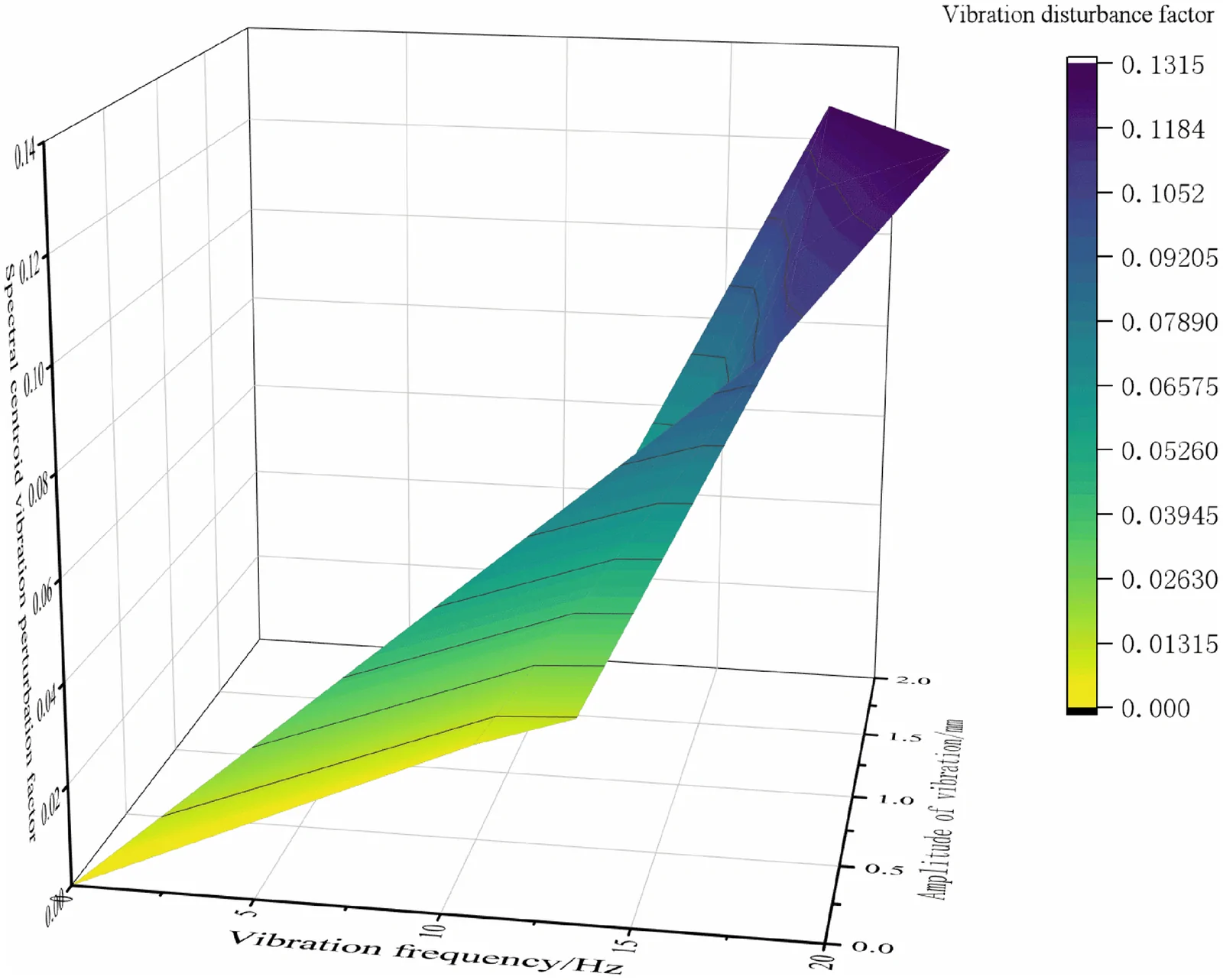
Changes in vibration frequency, vibration amplitude, and spectral centroid vibration disturbance factor under 1 wet-dry cycles.

**Fig 39:**
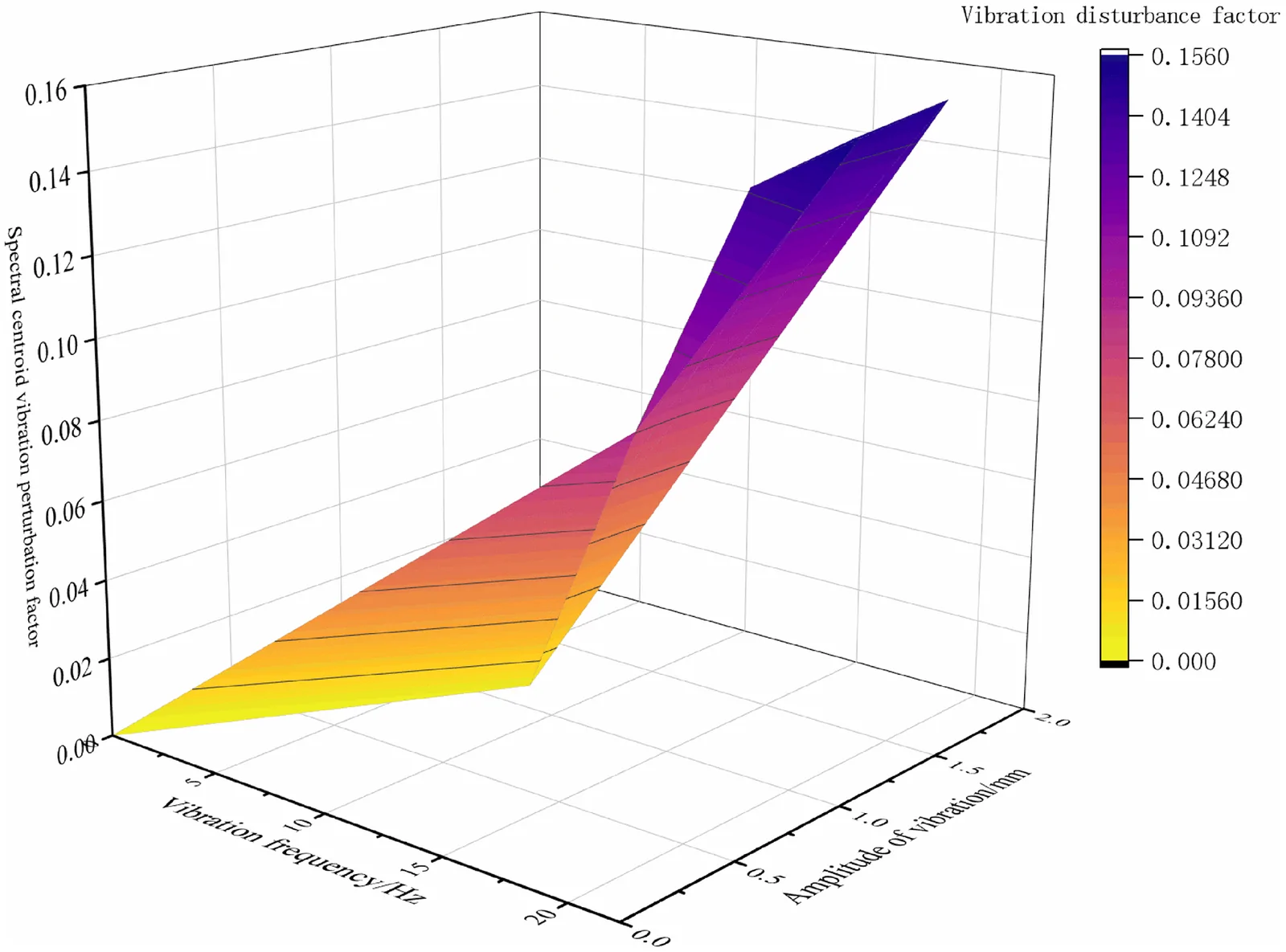
Changes in vibration frequency, vibration amplitude, and spectral centroid vibration disturbance factor under 3 wet-dry cycles.

**Fig 40:**
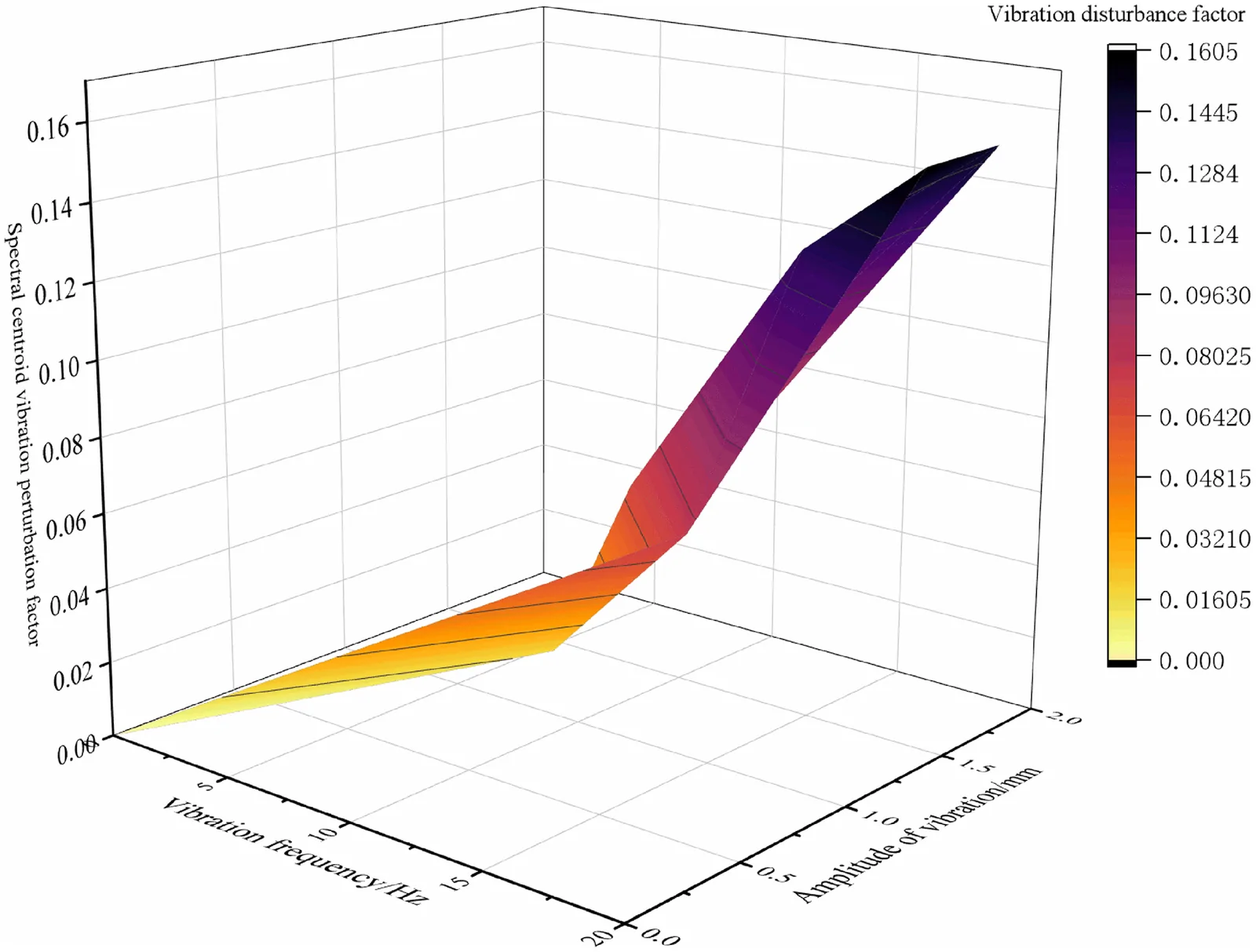
Changes in vibration frequency, vibration amplitude, and spectral centroid vibration disturbance factor under 5 wet-dry cycles.

**Fig 41:**
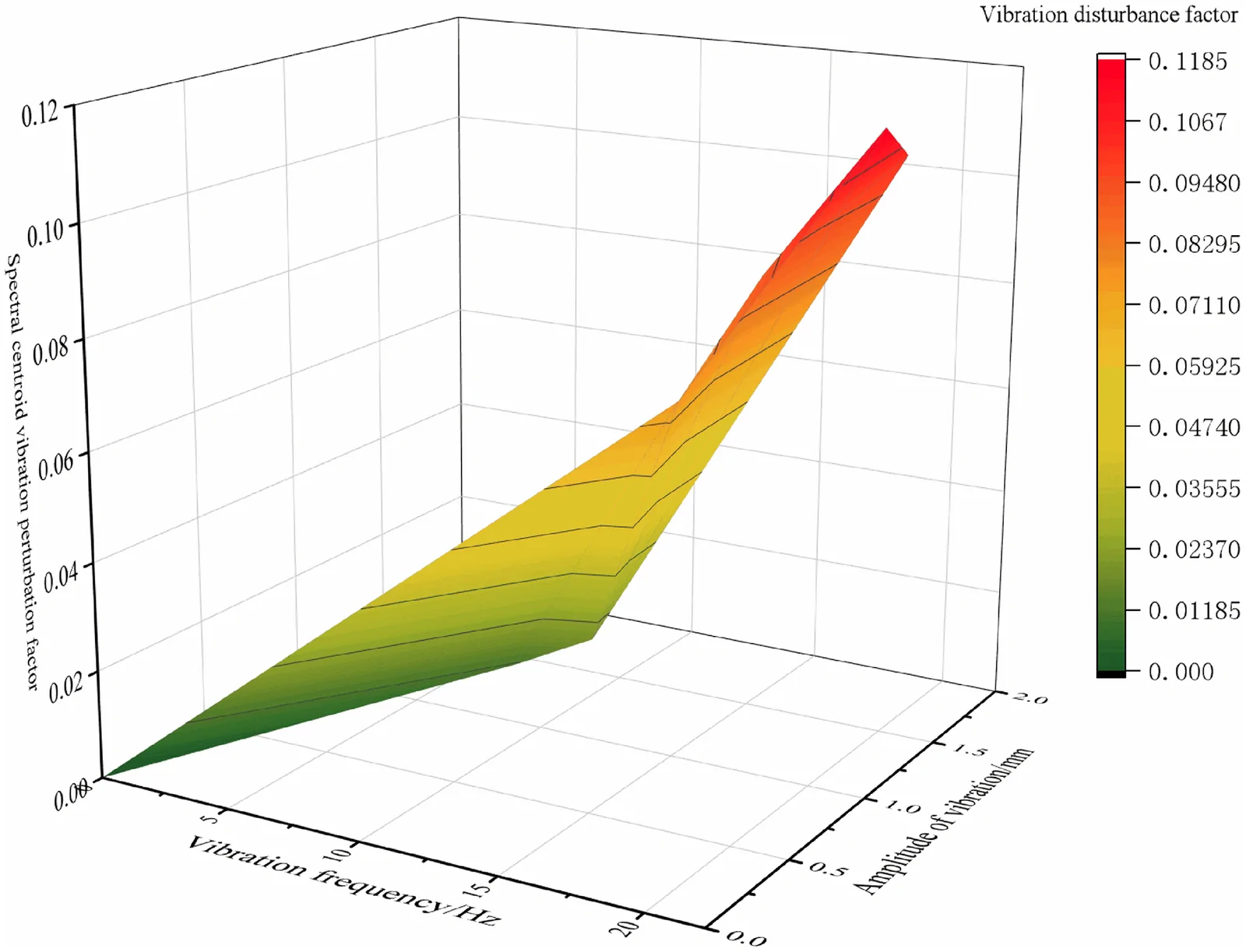
Changes in vibration frequency, vibration amplitude, and spectral centroid vibration disturbance factor under 7 wet-dry cycles.

The three-dimensional surfaces in Fig 37–41 intuitively show the nonlinear evolution of the spectral-centroid vibration-disturbance factor (Df) with vibration frequency and amplitude under different wetting-drying backgrounds. Overall, the damage factor increases markedly with increasing frequency and amplitude, indicating that vibration intensifies internal structural damage in the rock.

### Influence weights of vibration factors calculated by the entropy-weight method

The essence of the entropy-weight method is to determine weights objectively from the degree of data dispersion. In engineering evaluation, the larger the difference among indicator values, the smaller the information entropy, meaning that the indicator carries more effective information and should therefore be assigned a higher weight.

1. Data standardisation: let denote the indicator value of the ith experiment under the jth factor (frequency or amplitude), here taken as the spectral centroid. For the spectral-centroid disturbance factor, which is a negative indicator because smaller values indicate greater damage and stronger disturbance, standardisation is carried out as follows:

$$\begin{array}{c} {x^{\prime }}_{ij}=\frac{max\left(x_{j}\right)-x_{ij}}{max\left(x_{j}\right)-min\left(x_{j}\right)} \end{array}$$

2. Calculate the feature proportion:

$$\begin{array}{c} p_{ij}=\frac{{x^{\prime }}_{ij}}{\sum_{i=1}^{n}{x^{\prime }}_{ij}} \end{array}$$

3. Calculate the information entropy of the indicator:

$$\begin{array}{c} e_{j}=-k\sum_{i=1}^{n}p_{ij}ln\left(p_{ij}\right) \end{array}$$

K = ln(n) is a constant.

4. Calculate the entropy redundancy (difference coefficient):

$$\begin{array}{c} d_{j}=1-e_{j} \end{array}$$

5. Calculate the final weight:

$$\begin{array}{c} w_{j}=\frac{d_{j}}{\sum_{j=1}^{m}d_{j}} \end{array}$$

### Weight calculation of vibration factors based on the spectral-centroid factor

Taking the data for changes in vibration frequency and amplitude after 3 wetting-drying cycles as an example:

(1) For the frequency factor (j = 1): $\sum_{i=1}^{n}X_{i1}$ = 2.2619, P_11_ ≈ 0.0000 (a very small value is adopted to avoid evaluating ln(0)), P_21_ = 0.1573, P_31_ = 0.4005 and P_41_ = 0.4421. The entropy value is calculated as e_1_ = -(1/ln4) $\sum_{i=1}^{n}p_{i1}\text{\textasciitilde{}}ln{\text{\textasciitilde{}}p}_{i1}$≈0.734, and the difference coefficient is d_1_ = 1–0.734 = 0.266.
(2) For the amplitude factor (j = 2): $\sum_{i=1}^{n}X_{i2}$ = 2.7621, P_12_ ≈ 0.0000, P_22_ = 0.3017, P_32_ = 0.3363 and P_42_ = 0.3620. The entropy value is e_2_ = -(1/ln4) $\sum_{i=1}^{n}p_{i2}\text{\textasciitilde{}}ln\text{\textasciitilde{}}p_{i2}$≈0.790, and the difference coefficient is d_2_ = 1–0.790 = 0.210.

The final weight allocation is approximately: $w_{f}=\frac{d_{\text{1}}}{d_{\text{1}}+d_{\text{2}}}$ ≈0.559 and $w_{A}=\frac{d_{\text{1}}}{d_{\text{1}}+d_{\text{2}}}$≈0.441, respectively. The resulting frequency-amplitude weight fractions are shown in Fig 42.

**Fig 42:**
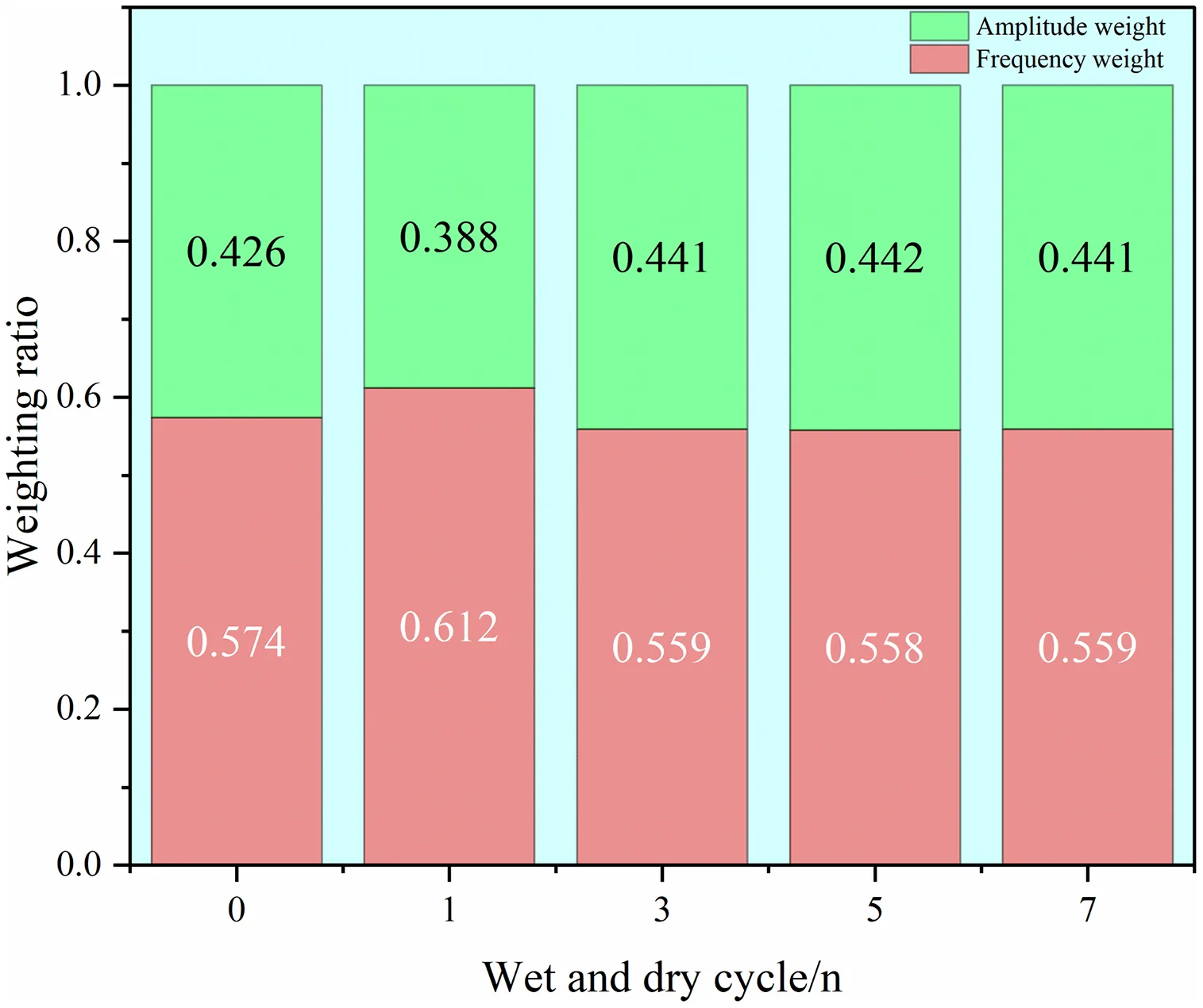
Weight fractions of frequency and amplitude under different numbers of wetting-drying cycles.

Based on the actual dispersion of the data, the influence weight of vibration frequency on the spectral-centroid damage factor, about 0.55–0.65, is greater than that of vibration amplitude, about 0.35–0.45. The mathematical results derived from the evolution of data variance not only satisfy the total probability constraint W_f_ + W_A_ = 1 at the macroscopic level, but also corroborate, in terms of micro-physical mechanisms, that high-frequency resonance and high-frequency displacement cycling are the core driving forces inducing microscopic contact interface scattering and fatigue degradation. The entropy-weight method therefore produces strong consistency between the internal logic of the data and the physical mechanism of fatigue-energy dissipation.

### Construction of the vibration-disturbance coefficient

Building upon the discussion in the previous section, high-frequency vibrations accelerate the initiation and propagation of micro-cracks within mudstone, a finding that challenges the traditional ‘amplitude-dominated’ paradigm. To quantify the specific degree of internal damage inflicted on slope materials by traffic load-induced vibrations, this study introduces the concept of the vibration disturbance coefficient, as expressed in Equation (6). This equation explicitly incorporates the core coupled factors governing vibration impacts on slopes—namely, vibration frequency characteristics and amplitude—to enable a more scientific and comprehensive assessment of the potential threats posed by vibration loads to slope stability.


$$\begin{array}{c} If_{A}={\left(\frac{f}{f_{max}}\right)}^{\omega_{f}}\times {\left(\frac{A}{A_{max}}\right)}^{\omega_{A}} \end{array}$$ (6)

In equation (6), I_fA_ denotes the vibration-disturbance coefficient. f_max_ and A_max_ are normalised using the upper limits of the experimental range, namely 22 Hz and 2.0 mm, respectively. W_f_ is the frequency weight, and W_A_ is the amplitude weight.It should be noted that the empirical parameters in I_fA_ are calibrated specifically within the traffic-induced vibration parameter envelope (f = 10–30 Hz, A = 0.5–2.0 mm, N = 0–10 cycles for strongly weathered mudstone. While the entropy weighting objectively captures wave attenuation physics, the quantitative values are bounded by these experimental conditions.

### Rationale for the formulation

1. Equation (6) combines information entropy and physical mechanism as dual constraints. The entropy-weight method automatically derives the weights from the intrinsic dispersion and variation information of the sample data, avoiding subjective weighting bias. It ensures that the weights Wf and WA frequency and amplitude strictly satisfy Wf+WA=1 the required constraints and reflects the physical rule that frequency-dominated resonance excitation governs vibration-energy transfer.
2. Normalization to eliminate dimensional barriers: Linear normalization is applied using the experimentally defined upper limits fmax=22Hz, Amax=22Hz as the baseline. This ensures that $\tilde{f}=f/22$ and $\tilde{A}=A/2.0$ both fall within the [0, 1] interval, thereby completely circumventing the numerical scale imbalance induced by the dimensional discrepancies between frequency (Hz) and amplitude (mm).
3. The functional structure of the exponent can capture accelerated deterioration. This power-law product structure naturally satisfies the required relation; for any $f_{\text{1}}<f_{\text{2}}\text{\textbackslash{}hspace\{1em}\}\frac{\partial \text{IFA}/\partial f}{\text{IFA}}=\frac{0.574}{f}$, taking the case of 0 wetting-drying cycles as an example, the increase in disturbance rate caused by a unit relative frequency increment decays as f increases. High-frequency vibration readily excites intrinsic structural modes, causing the number of stress cycles, and hence roadway service life, to vary exponentially with decreasing frequency ($N\propto f^{-k},\;k>1$) hereas the effect of vibration amplitude on strongly weathered mudstone specimens is approximately linear. The high exponential weighting in the parameters results in a sensitivity of the vibration disturbance value to f changes equal to $\frac{\partial \text{IFA}/\partial f}{\partial \text{IFA}/\partial A}=\frac{\omega_{f}}{\omega_{A}}\cdot \frac{A}{f}\approx 0.075$ times (calculated under typical operating conditions: f=18Hz, fmax=22Hz). Although the absolute partial-derivative ratio is moderated by the normalised variable range, the dominant role of frequency remains clear in terms of the dimensionless relative-change law embedded in the model.

### Relationship between damage factor and vibration-disturbance coefficient

In this section, measured acoustic data are introduced for cross-validation to test whether the vibration-disturbance coefficient formulated above can genuinely map the physical damage inside strongly weathered mudstone. By fitting both the spectral-centroid damage factor (Df), which characterises high-frequency energy dissipation, and the wave-velocity damage factor ($D_{v}=\frac{V_{w0}-V_{wfA}}{V_{w0}}$), which characterises macroscopic compactness, against the coordinate system defined by the vibration-disturbance coefficient, as shown in Fig 43–Fig 47, the scientific basis and applicability of the theoretical model can be established.

**Fig 43:**
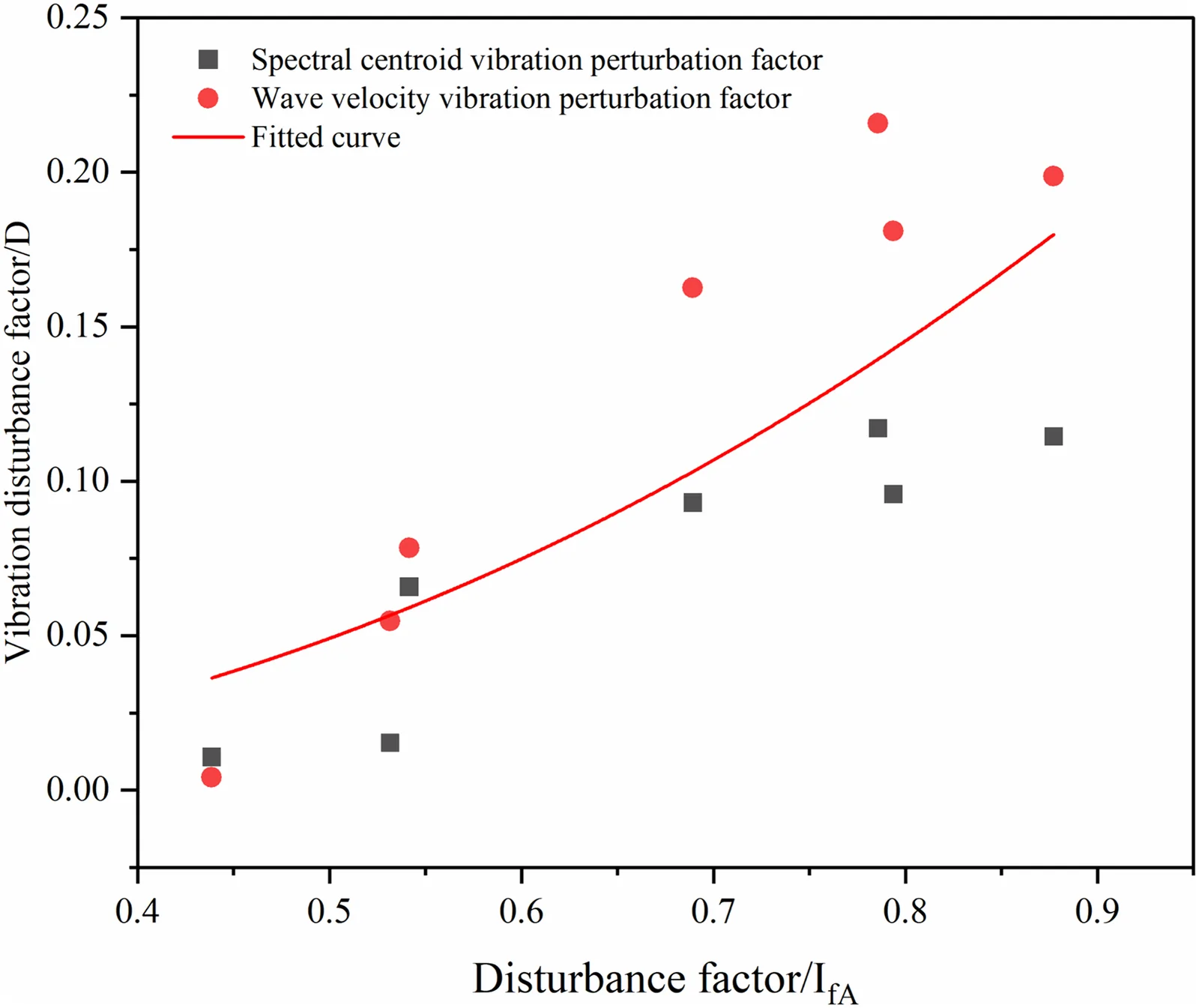
Relationship between multiple vibration disturbance factors and vibration disturbance coefficients under 0 wet-dry cycles.

**Fig 44:**
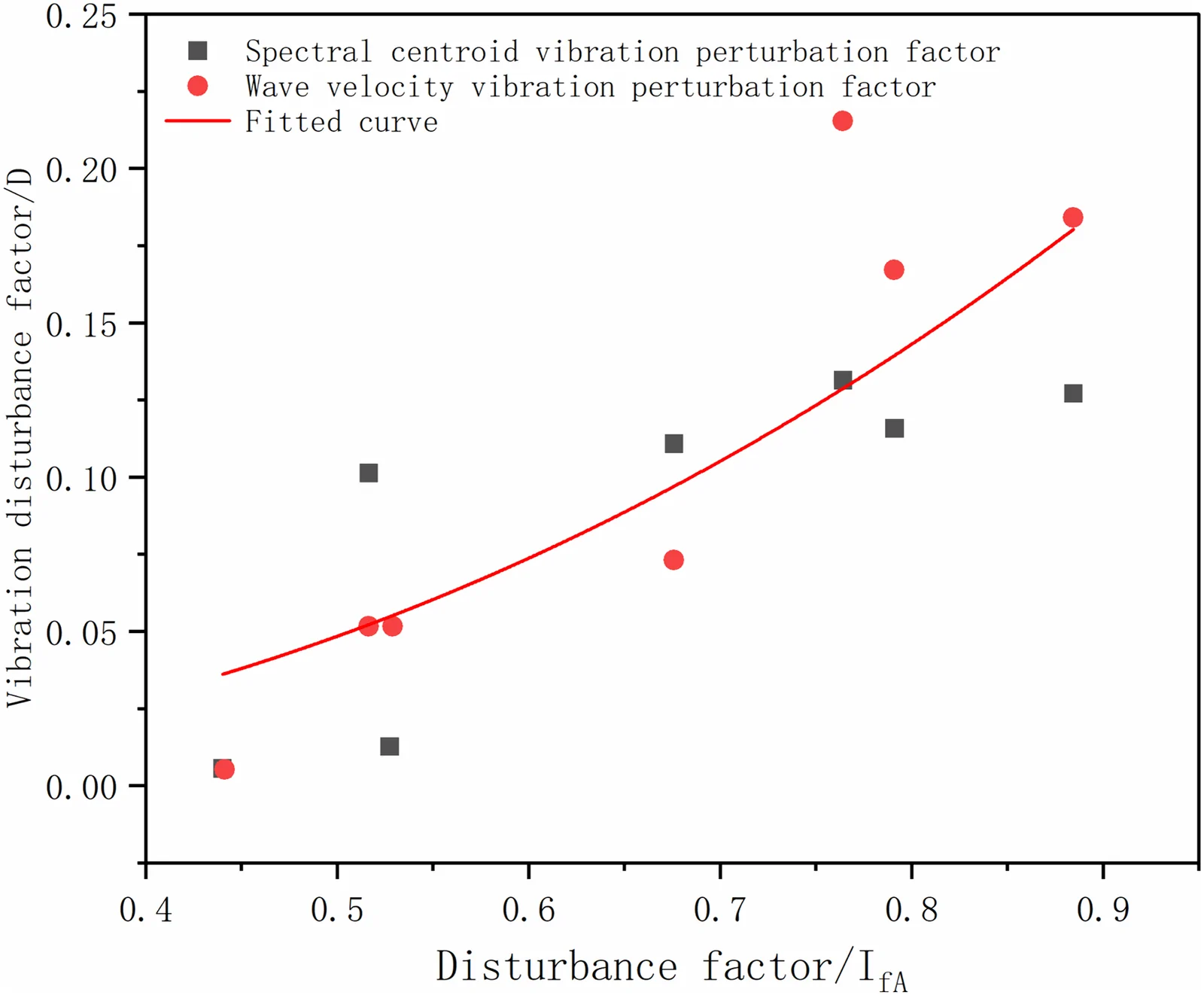
Relationship between multiple vibration disturbance factors and vibration disturbance coefficients under 1 wet-dry cycles.

**Fig 45:**
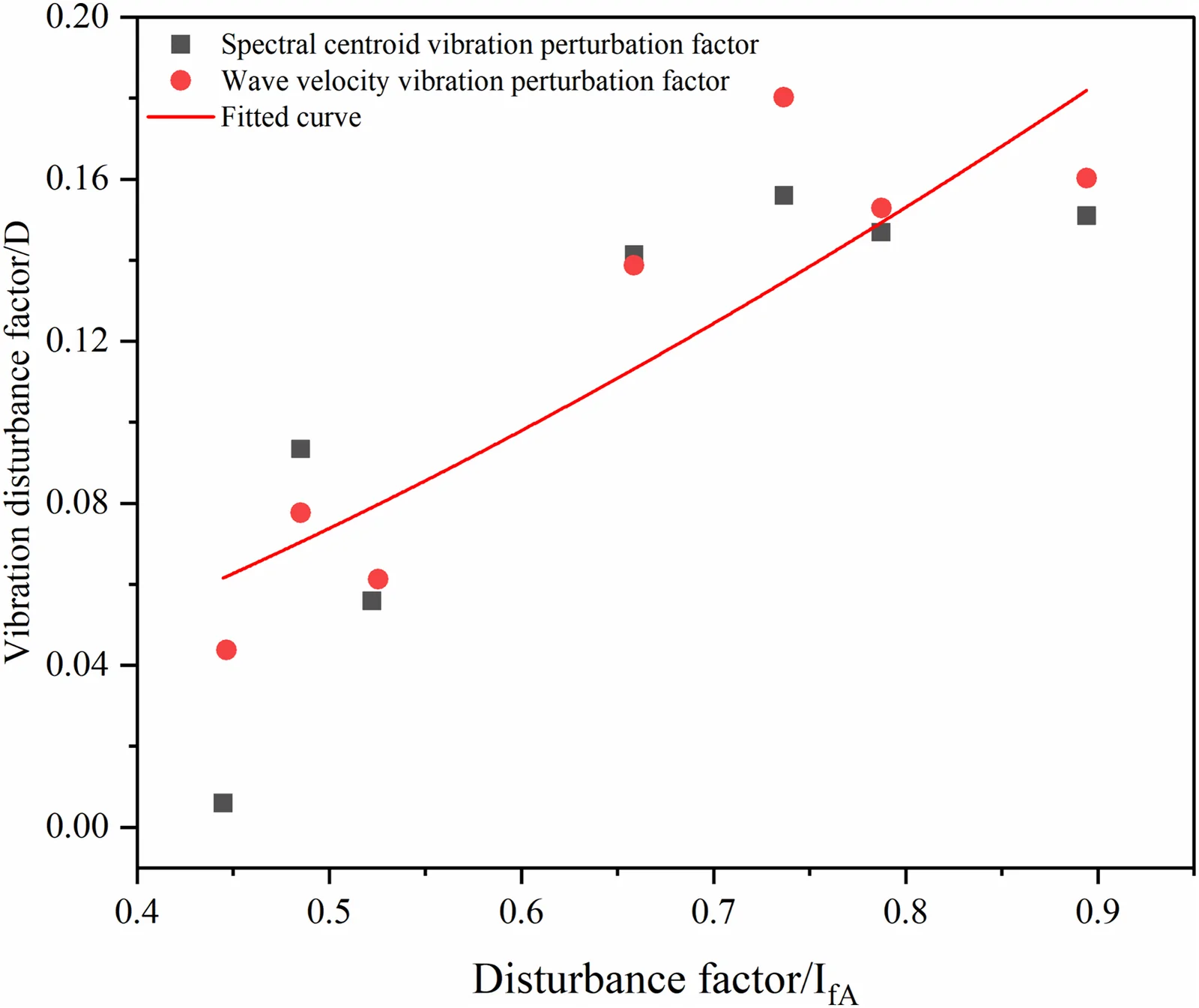
Relationship between multiple vibration disturbance factors and vibration disturbance coefficients under 3 wet-dry cycles.

**Fig 46:**
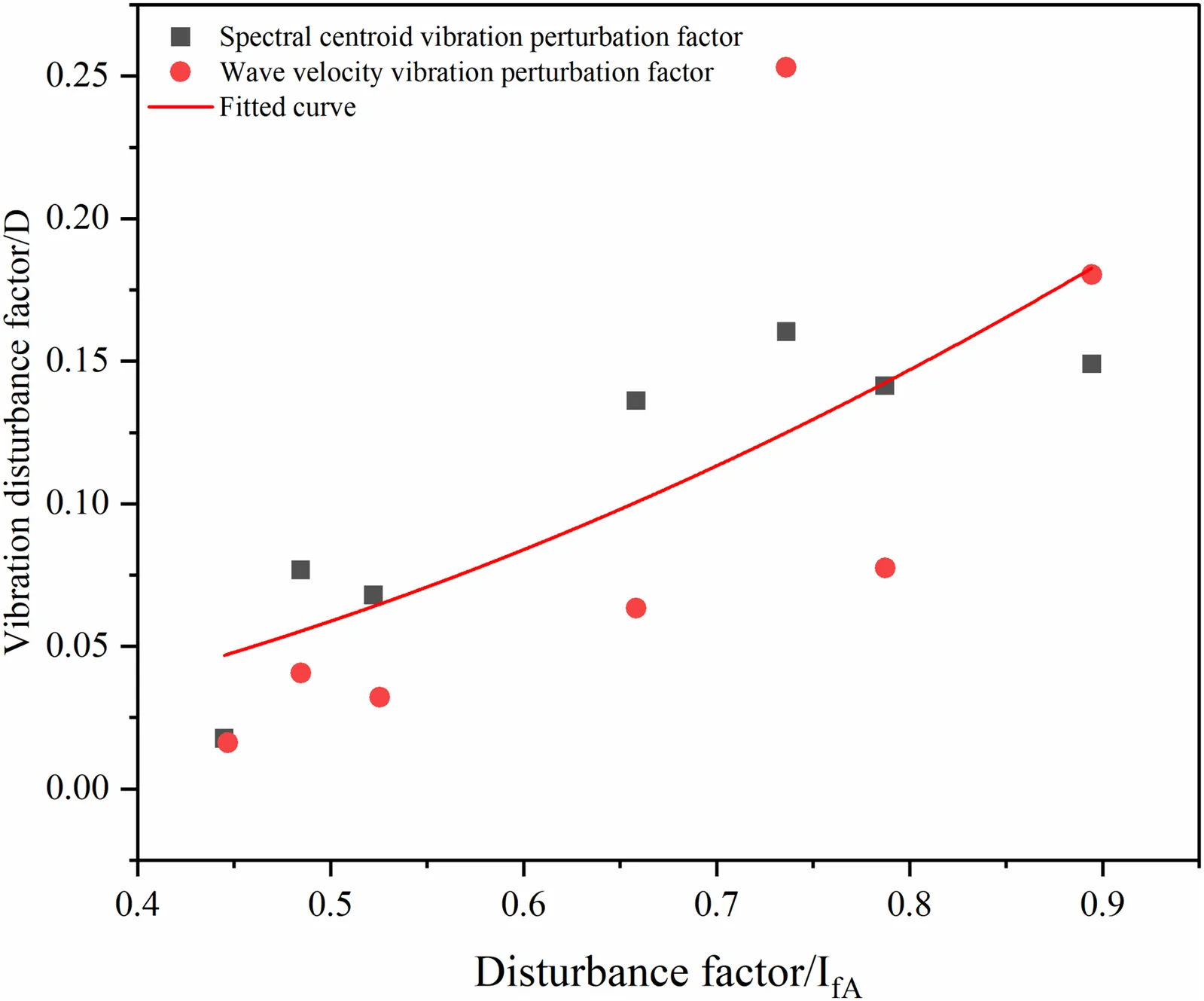
Relationship between multiple vibration disturbance factors and vibration disturbance coefficients under 5 wet-dry cycles.

**Fig 47:**
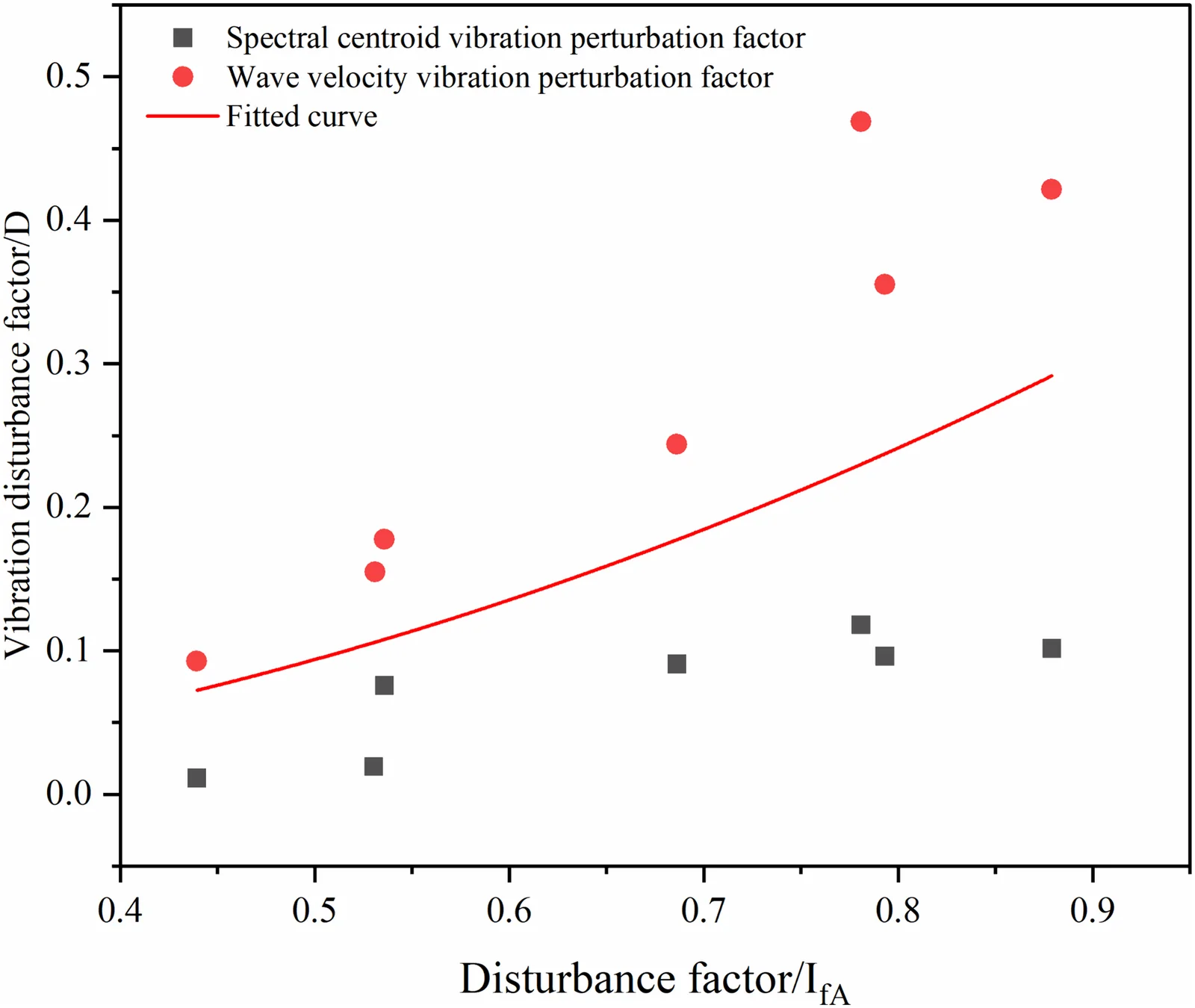
Relationship between multiple vibration disturbance factors and vibration disturbance coefficients under 7 wet-dry cycles.

The fitted results in Fig 43–Fig 47 show that when the vibration-disturbance coefficient (I_fA_) is used as the horizontal axis and the measured damage factor (D) as the vertical axis, the data for both indicators, namely the spectral-centroid disturbance damage factor D_f_ and the wave-velocity damage factor D_v_, cluster tightly around the fitted curves and display a pronounced positive correlation. This high degree of convergence of different physical descriptors, one representing high-frequency energy dissipation and the other macroscopic acoustic attenuation, on the same theoretical I_fA_ axis is especially important. It not only reveals the physical synchrony between micro-mechanisms and macro-response during fatigue damage in strongly weathered mudstone, but also fundamentally confirms the correctness and general applicability of the vibration-disturbance coefficient model through cross-comparison of multiple measured indicators.

### Strength analysis under coupled wetting-drying cycling and vibration disturbance

The preceding analyses have already cross-validated, using ultrasonic multi-band data, the scientific basis of the vibration-disturbance coefficient (I_fA_) for characterising the evolution of internal micro-fatigue damage in strongly weathered mudstone. In actual highway cut-slope engineering, however, instability hazards are ultimately governed by the decline in macroscopic load-bearing capacity. This section therefore integrates microscopic acoustic indicators with macroscopic mechanical test results to analyse the synergistic deterioration of peak strength caused by hydraulic action during wetting-drying cycling and by traffic-induced vibration disturbance, and to establish a strength-evolution model under multi-field coupling.

Table 5 lists the vibration-disturbance coefficient under different numbers of wetting-drying cycles. Combined with the measured uniaxial compressive strength data, these values were used to construct a three-dimensional evolution surface of wetting-drying cycle number, vibration-disturbance coefficient and peakstrength, as shown in Fig 48.

**Table 5:** Vibration-disturbance coefficient under different numbers of wetting-drying cycles.

Vibration frequency (Hz)	Vibration amplitude (mm)	I_fA_ at 0 wetting-drying cycles	I_fA_ at 1 wetting-drying cycle	I_fA_ at 3 wetting-drying cycles	I_fA_ at 5 wetting-drying cycles	I_fA_ at 7 wetting-drying cycles
0	0	0.0000	0.0000	0.0000	0.0000	0.0000
9	1.0	0.4386	0.4401	0.4447	0.4448	0.4394
12	1.0	0.5316	0.5274	0.5222	0.5222	0.5303
18	0.5	0.5415	0.5165	0.4851	0.4845	0.5358
18	1.0	0.6892	0.6759	0.6586	0.6583	0.6862
18	1.5	0.7937	0.7911	0.7871	0.7871	0.7931
18	2.0	0.8772	0.8845	0.8941	0.8943	0.8789
22	1.0	0.7857	0.7642	0.7366	0.7361	0.7808

**Fig 48:**
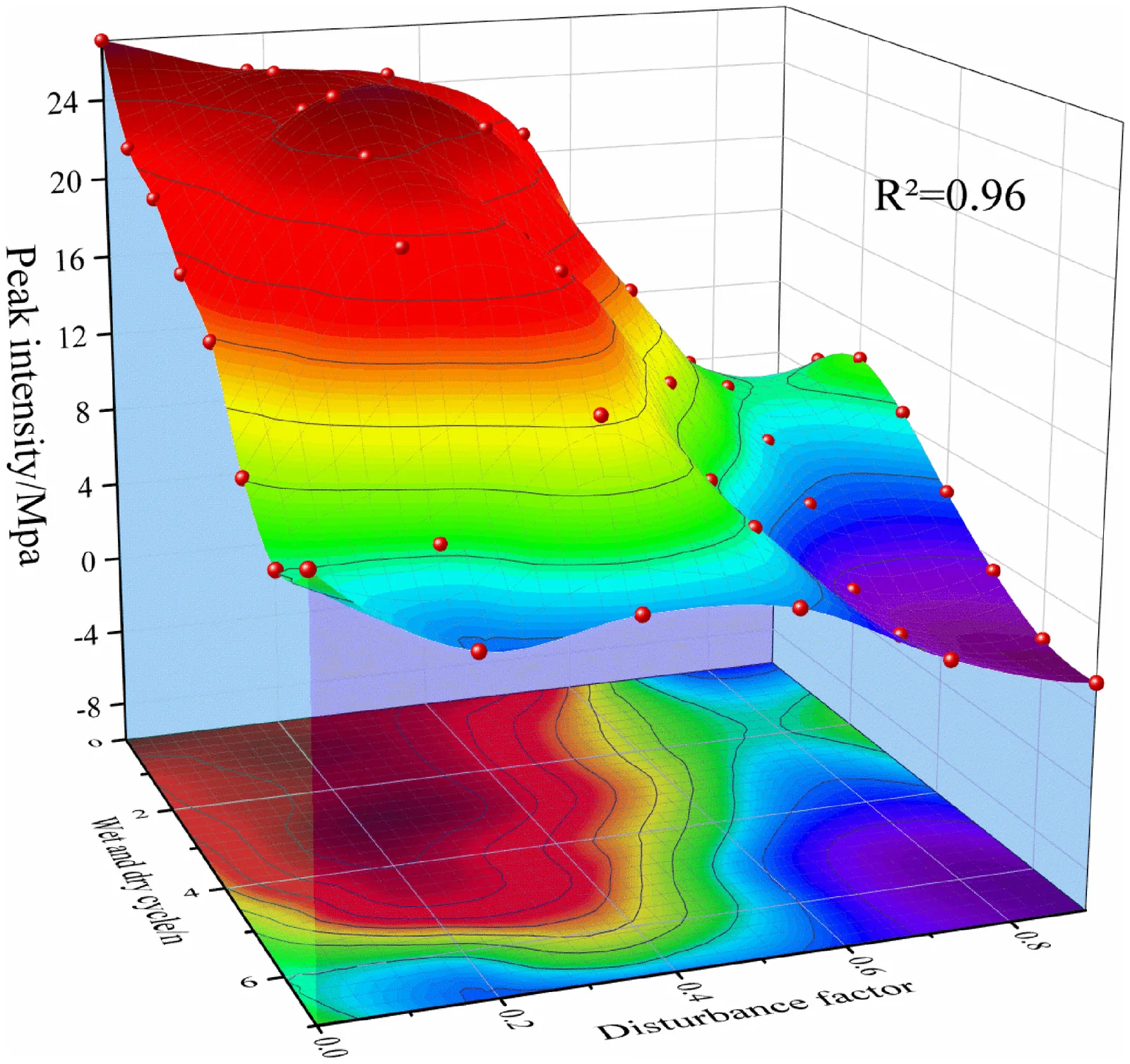
Wetting-drying cycle – vibration-disturbance coefficient – strength.

The macroscopic mechanical degradation of strongly weathered mudstone exhibits a clear nonlinear synergistic feature that can be described as vibration-disturbance-catalysed collapse. To characterise in a focused manner how dynamic loading weakens strength under different wetting-drying backgrounds, we constructed a parameterised three-dimensional surface equation that quantitatively captures vibration-energy absorption and physical deterioration in mathematical form, as shown in equation (7).


$$\begin{array}{c} q=q_{\text{0}}\left(1-c\cdot N^{d}\cdot IfA^{k}\right) \end{array}$$ (7)

In this model, the attenuation of peak strength (q) is attributed primarily to the vibration-energy absorption term$\left(1-c\cdot N^{d}\cdot IfA^{k}\right)$. This term quantitatively describes the sustained weakening effect of the vibration-disturbance coefficient (IfA) on mudstone strength in the context of the wetting-drying cycle number (N). Three-dimensional nonlinear surface fitting yields a coefficient of determination R2 of 0.96, indicating that the fitted surface captures and explains 96% of the variation in peak strength under coupled loading, and thus has high statistical reliability and predictive accuracy. The evolution of the three-dimensional strength surface further shows that the structural loosening produced by early wetting-drying alternation provides physical space for vibration-energy dissipation, enabling the parameters that represent vibration sensitivity, c, d and k, to amplify the deformation response rapidly. Once a high vibration-disturbance coefficient is superimposed, the surface develops a pronounced downward collapse, directly triggering the transition of microcracks from accumulation to pervasive through-going coalescence. This empirical expression therefore not only maps the physical essence of microscopic ultrasonic energy attenuation, but also provides a scientific mathematical representation of the complex multi-field acceleration mechanism governing strongly weathered mudstone under vibration-disturbance-dominated conditions.

## Conclusions

Mechanical testing and ultrasonic non-destructive testing were carried out on strongly weathered mudstone under coupled wetting-drying cycling and traffic-vibration loading to investigate the microstructural evolution and macroscopic deterioration mechanisms of this material in northwestern Hebei under multi-field coupling. The main conclusions are as follows:

(1) Wetting–drying cycles drive repeated swelling and shrinkage of hydrophilic minerals (illite and montmorillonite), causing uniaxial compressive strength (UCS) to decay from 27.00 MPa to 10.51 MPa (a 61.1% drop). Superimposed dynamic vibrations (22 Hz) drastically accelerate this structural breakdown, collapsing peak strength to a residual 2.18 MPa (a 91.9% total reduction) and driving elastic modulus from 510.13 MPa down to 55.67 MPa, indicating a clear softening transition from brittle to plastic behavior and significant non-linear synergistic damage.
(2) Active ultrasonic pulse parameters quantitatively capture micro-damage evolution. As internal defects accumulate, longitudinal wave velocity (Vp) decreases from 1.696 km/s to 0.538 km/s, material damping ratio increases from 0.15 to 2.94, and the spectral centroid shifts down from 50.29 kHz to 38.86 kHz. Changes in spectral centroid exhibit a tight linear correlation with peak strength and elastic modulus without saturating in low-strength states, validating its function as a highly sensitive non-destructive indicator.
(3) Persistent disturbance from traffic dynamic loading further propagates and connects internal microcracks, producing a synergistic acceleration effect with wetting–drying cycling. Entropy-weight analysis reveals that vibration frequency carries a higher influence weight (Wf = 0.55–0.65) than amplitude (WA = 0.35–0.45), confirming that high-frequency resonance and displacement cycling are the primary drivers of micro-fatigue deterioration.
(4) Using the entropy weight method, this study proposes a vibration factor weight calculation method based on the ultrasonic frequency spectrum centroid. The vibration disturbance coefficient I_fA_ was constructed to quantitatively characterize the vibration-induced damage effects on the rock mass, systematically analyzing its evolution laws under various wetting-drying cycles and vibration parameters. Furthermore, the mechanisms of micro-crack initiation, propagation, and coalescence within the mudstone were back-analyzed. By specifically incorporating I_fA_ into the parameterized three-dimensional strength-surface model (Equation 7), this approach quantitatively links non-destructive ultrasonic evaluation with macroscopic mechanical degradation.This approach enables the non-destructive and real-time evaluation of internal damage in highly weathered mudstone subjected to coupled wetting-drying and vibration effects, and subsequently allows for the estimation of peak strength. Overall, this method offers a convenient and cost-effective monitoring tool for the early identification of slope hazards during the operational period (e.g., early warning for strength degradation and risk assessment of slope failure). It overcomes the limitations of traditional single-indicator or destructive testing methods, holding practical guiding significance for the long-term stability evaluation and protection design of slopes under similar geological conditions.
(5) Beyond the experimental observations, this study advances the understanding of rock damage mechanics by quantifying the dynamic synergistic degradation of strongly weathered mudstone under coupled environmental and traffic-vibration loading, thereby addressing the inherent limitations of traditional static single-factor evaluation frameworks. From a practical engineering perspective, the quantitative linkage established between ultrasonic wave parameters (such as wave velocity decay and spectral centroid shift) and macro-mechanical fatigue provides a robust non-destructive evaluation protocol. These findings can be directly implemented into field health monitoring systems and non-destructive testing (NDT) practices to enable real-time stability assessment, early hazard warning, and targeted maintenance for mudstone subgrade slopes along transportation corridors subjected to seasonal weathering and dynamic traffic disturbances.

## Supporting information

S1 Data Minimal dataset underlying the findings. (XLSX)
